# Impact of employment and income support interventions on the health of young adults with episodic disability: Findings from a systematic review

**DOI:** 10.5271/sjweh.4133

**Published:** 2024-03-01

**Authors:** Arif Jetha, Lahmea Navaratnerajah, Sebastian Kondratowski, Meagan Parmassar, Lori B Tucker, Monique AM Gignac

**Affiliations:** 1 Institute for Work & Health, Toronto, ON, Canada.; 2 Dalla Lana School of Public Health University of Toronto, Toronto, ON, Canada.; 3 BC Children’s Hospital, Vancouver, BC, Canada.; 4 University of British Columbia, Vancouver, BC, Canada.

**Keywords:** social determinant of health, work intervention, young adulthood

## Abstract

**Objective:**

Young adults living with episodic disabilities face unpredictable disruptions to their employment and health. Our study aimed to examine the impact of employment and income support interventions on the health and well-being of young adults living with episodic disabilities.

**Methods:**

We conducted a systematic review of peer-reviewed intervention studies published in 2001–2021 in industrialized contexts. Two independent reviewers screened titles, abstracts and full-texts. We undertook a narrative synthesis of eligible articles.

**Results:**

Our search yielded 15,269 published articles, of which only five studies were eligible for evidence synthesis. All articles were appraised as being of medium quality. Four interventions focused on young adults living with mental health conditions. Two were based in clinical settings; three were based in community-based settings. Each employment intervention exhibited improvements in health outcomes. Three studies examined the impact of supported employment interventions that were particularly beneficial to improving work and health outcomes.

**Conclusion:**

Involvement in employment interventions could provide benefits for the health of young adults with episodic disability. Our systematic review highlights the need to for research to elaborate on the ways in which employment interventions can impact the health and well-being of young adults living with different episodic disabilities.

Young adults living with episodic disabilities can experience unpredictable disruptions to their employment as they enter and advance within the labor market. The most prevalent physical (e.g., juvenile arthritis, inflammatory bowel disease) and mental health (e.g., anxiety, depression) chronic conditions are often categorized as episodic, with periods of good health interrupted by flares of poor health (1). Some estimates suggest one out of six of working-aged adults with disabilities report fluctuating and unpredictable limitations (2).

The unpredictability of an episodic disability can be stressful and impact the health and quality of life of young adults. Finding and sustaining employment can also be challenging, especially managing fluctuations in symptoms while also navigating the changing demands of work (3–5). Many episodic conditions have few visible signs creating challenges communicating about work support needs (6, 7). Living and working with an episodic disability may have different impacts at various life stages; difficulties at the early career phase can have an economic scarring effect that impact both employment opportunities and health across the life course (8, 9).

To manage their health and well-being, many young adults with episodic disabilities rely on employment not only for income but also for resources (e.g., extended health benefits, drug coverage) that may improve or sustain good health (9). To examine this further, our study reviewed employment and income support interventions and their impact on the health and well-being of young adults living with episodic disabilities.

## Methods

A systematic review addressed the study aim (10). To inform the search strategy and synthesize key findings, consultations with knowledge users were held. The review was registered with PROSPERO (CRD42021268354) and met 2020 PRISMA Guidelines.

### Literature search

The search strategy captured employment or income support intervention studies for young adults [i.e., sample mean age 16–35 (range 16–45) years] living with any episodic disability (Table 1). Our search was restricted to intervention studies conducted in OECD countries, those that used quantitative methodologies, and were published in the last 20 years in English, French or Spanish. We examined the effect of employment or income support inteventions on any health outcome. Medline, Embase, PsycINFO, CINAHL, Sociological Abstracts, ASSIA, ABI Inform, and Econ lit were searched using database-specific controlled vocabulary terms and keywords (Supplementary material, www.sjweh.fi/article/4133, Table S1).

**Table 1:** Inclusion and exclusion criteria using PICO framework

PICO category	Inclusion criteria	Exclusion criteria
Population	Young adults (sample mean age 16-35 years and age range 16 and 45 years) Living with any episodic health condition Study sample located in an OECD labor market country	Study sample not classified as a young adult or with an episodic disability Study sample located in a non-OECD context
Intervention	Any intervention that targeted employment or income support	Intervention with no employment or income support component
Comparison	Any comparator group (population not taking part or exposed to the intervention)	No comparison group
Outcome	Any physical or mental health outcome, measure of health-related quality of life or assessment of well-being	Studies evaluating the effectiveness of an intervention which did not assess outcomes of interest

### Relevance screening

Two reviewers independently screened titles and abstracts and full-texts for relevancy using DistillerSR (11). Disagreements were discussed in meetings. Reference lists of included articles were checked to identify additional relevant articles.

### Quality appraisal and data extraction

A quality appraisal tool for studies in work and health was used to assess the internal, external, and statistical validity of eligible articles (10). The tool assessed risk of bias in study design and objectives, recruitment procedures, outcome and exposure measurement and analysis (Supplementary Table S2). Two reviewers appraised each relevant article. A final weighted sum score of the quality criteria was generated, converted to a percentage score, and categorized as high (≥85% of quality appraisal score), medium (50–84% of total quality appraisal score) or low quality (<50% of quality appraisal total score). Consensus on scores was reached in a team meeting.

### Evidence synthesis

The limited number of eligible studies identified by the review coupled with variability in the observation length, intervention type, sample characteristics, outcomes meant that we were unable to calculate pooled effect estimates. Instead, a narrative synthesis described the findings.

## Results

**Figure 1:**
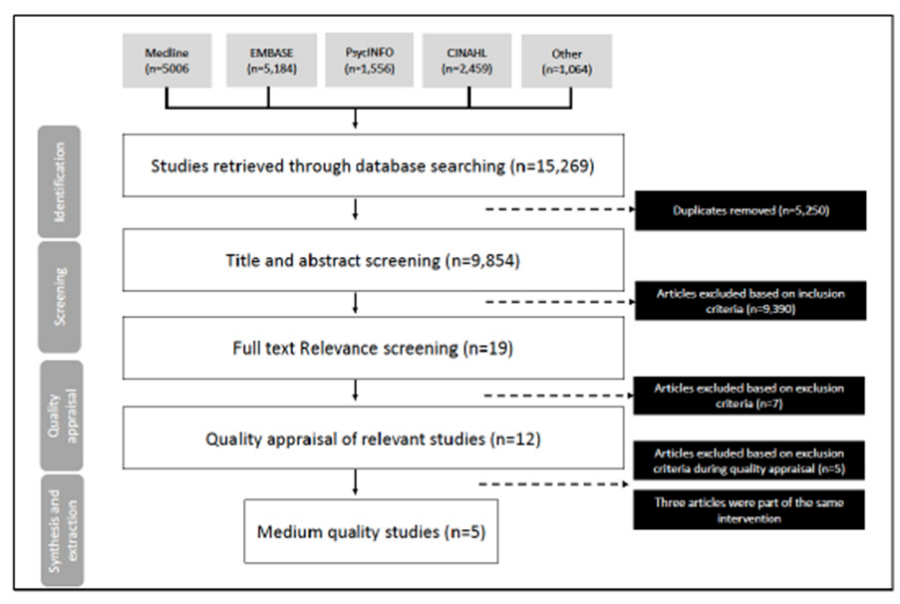
Systematic review flowchart.

Our search yielded 15,269 articles published between years 2001–2021. Only five eligible employment interventions focused on young adults with an episodic disability and assessed health impacts (Figure 1). All articles were appraised as medium quality. A summary of each study is provided in Table 2.

**Table 2:** Description of studies identified in systematic review examining income and employment interventions on health

Author, Year, Country	Study objectives	Study design, length of data collection, and setting	Sample size description	Episodic disability group	Employment and income outcomes (measures utilized)	Health and well-being outcomes (measures utilized)
Geenen et al, 2015, USA	To examine whether youth who participated in the Better Future’s project showed improved outcomes when compared to youth randomized to a control group who were offered standard services.	Randomized controlled trial 16 months Community setting	N=67 (36 intervention; 31 control) Mean age 16.8 ± 0.62 years 52.2% female	Youth with mental health challenges in foster care	Employment status Career decision self-efficacy	Improvements in mental health (Youth Efficacy/Empowerment Scale- Mental Health) Mental health recovery (Mental Health Recovery Measure) Hopelessness (modified Hopelessness Scale for Children) Quality of life (Quality of Life Questionnaire)
Liljeholm et al, 2020, Sweden	To determine how the Södertälje Supported Employment and Education model, an integrated mental health and vocational support intervention, can impact mental health among young adults with mood disorders.	Prospective Longitudinal pre-post intervention study 12 months Community setting	N=42 Mean age 21 (range 18–28) years 64.3% female	Young adults with major depressive (93%) or bipolar disorder (7%)	Engagement in everyday life (Profiles of Occupational Engagement in people with Severe mental illness [POES])	Mental health symptomology and recovery (Montgomery-Asberg Depression Rating Scale) Quality of life (Manchester Short Assessment of Quality of Life [MANSA])
Menrath et al, 2019 Germany	To study the efficacy and impact of the Modular Transition (ModuS-T) patient education program, designed to support young adults living with chronic conditions manage their disease and transition to adulthood, when compared to young adults in usual care.	Quasi-randomized control study 4 weeks Clinical setting	N=300 Mean age 17.6 ± 1.6 years 47% female	Young people with chronic conditions: asthma (5.3%), attention deficit disorder /hyperactivity disorder (18.7%), type 1 diabetes (19.7%), phenylketonuria (2.3%), inflammatory bowel disease (16.0%), cystic fibrosis (2.7%), chronic kidney disease (4.0%), epilepsy (7.7%), organ transplantation (7.3%), juvenile idiopathic arthritis (7.7%), esophagus atresia (12.0%), Ehlers-Danlos syndrome (2.7%)	Work preparedness (Transition Competency Scale)	Health-related quality of life (DISABKIDS Chronic Generic Measure; SF-8) Self-reported health Engagement in health care (German Patient Activation Measure for Adolescents) Health condition-related knowledge (Transition Competence Scale) Health care competence (Transition Competence Scale)
Kane et al, 2016 USA Rosenheck et al, 2017 USA Rosenheck et al, 2017 USA	To compare the NAVIGATE coordinated specialty care program on receipt of disability income support benefits, employment, treatment and first-episode psychosis outcomes when compared to usual community care.	Clustered randomized trial 24 months Clinical setting	N=404 (223 intervention; 181 community care) Mean age: (intervention) 23.18 ± 5.21 years; (community care) 23.08 ± 4.90 years 27% female	Individuals with first episode of psychosis	Work participation Number of days participating in work Receipt of disability income support	Schizophrenia symptom severity (Positive and Negative Syndrome Scale) Depression symptoms (Calgary Depression Scale for Schizophrenia) Mental health illness severity (Clinical Global Impressions Severity Scale) Quality of life (Heinrichs-Carpenter Quality of Life Scale)
Sveindottir et al, 2020 Norway	To study the role of the Individual placement and support (IPS) approach for young adults with mental and behavioural conditions, at risk of early work disability when compared to traditional vocational rehabilitation.	Two-armed randomized control trial 12 months Community setting	N=96 (50 intervention group; 46 control group) Mean age: (intervention) 23.96 ± 3.46 years (control) 23.85 ± 3.04 32% female	Individuals with mental and behavioral conditions	Paid competitive employment Proportion of participants working ≥20 hours per week Total number of hours worked	Disability (World Health Organization Disability Assessment Schedule [WHODAS] 2.0) Psychological distress (Hopkins Symptom Checklist) Severity of subjective health complaints (Subjective Complaints Inventory) Fatigue (Chalder Fatigue Questionnaire) Coping, helplessness, and hopelessness (Theoretically Originated Measure of the Cognitive Activation Theory of Stress [TOMCATS]) Alcohol consumption (Alcohol Use Disorders Identification Test consumption questions [AUDIT-C]) Drug use (Drug Use Disorders Identification consumption questions [DUDIT-C]) Global well-being (Cantril Ladder Scale)

The five employment interventions found improvements in health outcomes (Table 3). A randomized controlled trial (RCT) of a personalized coaching initiative in the United States offered mentorship to young adults with mental health conditions to meet career and educational goals and build career self-determination. Although the intervention did not contribute to a change in employment over the six months following the ten-month intervention period, it was associated with improved career self-efficacy. Intervention recipients reported greater mental health but no difference in quality of life when compared to the control group (12).

**Table 3:** Description of study interventions and their impact on income and employment outcomes and health outcomes

Study	Description of intervention	Intervention’s impact on income and employment outcomes	Intervention’s impact on health and well-being outcomes	Quality appraisal
Geenen et al, 2015	The Better Futures project is a personalized coaching intervention program that provides foster youth with mental health challenges to identify and work on their personal, education or career goals, build self-determination skills through mentorship workshops with advisory peers who have had similar experience in foster care and dealing with their mental health challenges.	No significant difference in the proportion of participants who were employed at follow-up when comparing the intervention group to the control group. Over the study period, the intervention group reported significantly greater career decision self-efficacy when compared to the control group F (3, 124)=6.06; P=0.0007.	Over the study period, the intervention group reported significantly greater efficacy in mental health care management when compared to the control group F(3, 118)=9.07; P<0.0001). While the intervention group reported greater mental health recovery over the study period when compared to the control group, the difference was non-significant F (3, 180)=2.55, P=0.06). There was no difference between intervention and control groups in quality of life over the study period F (3, 118)=2.29, P=0.82). There was a significant decline in hopelessness among the intervention group, when compared to the control group over the study period F (3, 62)=2.79, P=0.048).	Moderate
Lijeholm et al, 2020	The Södertälje Supported Employment and Education model is an integrated intervention targeted towards young adults with major depressive or bipolar disorders to improve their mental health and support employment. Intervention included integrated vocational services with mental health services, personalized benefit counselling and employer relationship building. All services were delivered by a case manager and aligned with the client’s preferences and needs.	Participants reported statistically significant improvements to engagement in everyday life activities at 12-months when compared to baseline (P=0.002).	Although depression symptomology decreased at follow-up when compared to baseline, the relationship was not statistically significant (P=0.241). Participants indicated greater quality of life at follow-up when compared to baseline (P=0.007). At 12 months, participants who reported engagement in everyday life activities were significantly less likely to report depression severity (r=-0.313, P<0.05) and reported higher quality of life (r=0.470, P<0.05).	Moderate
Menrath et al, 2019	The Modular transition (ModuS-T) education program aims at empowering patients with chronic conditions to take responsibility in managing their disease. Modules on insurance, the health care system, career, social networking, coping and stress management, health promotion behaviours, were delivered participants and their parents/caregivers. Group discussions, role playing, and case studies were utilized to deliver the training.	Young adults in the intervention group observed significantly greater scores for work preparedness (TC-a) over the duration of the study when compared to those in the control group F=27.3 (p < 0.001).	No significant difference existed when comparing the intervention group to the control group on health-related quality of life when using the DISABKIDS. Young adults in the intervention group showed significantly higher engagement in health care when compared to the control group over the study period F=8.1, P<0.001). Young adults in the intervention group showed significantly higher health condition-related knowledge when compared to the control group over the study period F=22.9, P<0.001). Young adults in the intervention group showed significantly higher health care competence when compared to the control group over the study period (F=56.9, P<0.001).	Moderate
Kane et al, 2016 Rosenheck et al, 2017 Rosenheck et al, 2017	NAVIGATE intervention is a four-component training approach that includes: 1) customized medication management training; 2) family psychoeducation 3) self-management training focused on building resilience and 4) supported employment and education.	Compared to community care, intervention participants were significantly more likely to report work participation. However, the main effect by time was most significant for those who participated in >3 supported employment/education sessions (t=0.16, P<0.05). No significant differences between intervention and community care when comparing number of days in work (t=0.097, P=0.16). No significant differences between intervention and community care when comparing receipt of disability income support (t=4.25, P=0.71).	Intervention participants experienced significantly greater improvement in quality of life over the study period than those in community care (t=2.45, P=0.015). Intervention participants reported a significant decrease in schizophrenia symptom severity when compared to those in community care (t=-2.41, P=0.02). Intervention participants indicated a significant decrease in depressive symptoms when compared to those in community care (t=-2.15, P=0.032). No significant difference in mental health illness severity between intervention participants when compared to those in community care over the study period (t=-1.52, P=0.13).	Moderate
Sveindottir et al, 2020	The individual placement and support (IPS) model of supported employment is an intervention approach that is targeted towards enhancing competitive employment outcomes for patients with severe mental health illness through focusing on patient goals and preferences, providing long-term personalized support, access to integrated services and counselling. The intervention was facilitated by a job specialist who matched the candidate with a job and provided ongoing support following a job through established IPS principles.	IPS participants were significantly more likely to hold competitive employment at 12-month follow-up compared to the traditional vocational rehabilitation group (OR=10.39, 95% CI 2.79-38.68). A significantly higher proportion of IPS participants reported working ≥20 hours per week at the 12-month follow-up compared to the traditional vocational rehabilitation group (OR=8.75, 95% CI 1.83-41.75). A significantly higher proportion of IPS participants reported working more hours at the 12-month follow-up compared to the traditional vocational rehabilitation group (Cohen’s D=0.70, p=0.002).	Participants in the intervention group reported significantly less health complaints (P=0.017), helplessness (P=0.017) and hopelessness (P=0.006), and drug use (P=0.036) when compared to the traditional vocational rehabilitation group at 12-months. Participants in the intervention group reported significantly less disability (P=0.038) and greater perception of future well-being (P=0.038) when compared to the traditional vocational rehabilitation group at 12-months.	Moderate

Three supported employment interventions in community and clinical settings for young adults with mental health conditions were identified (13–15). The interventions provided employment placement services, career counselling and health-related self-management assistance. Findings highlighted improved occupational engagement, participation in employment, and hours worked (13–15), as well as improvements in quality of life and well-being, declines in depressive symptom severity, fewer health complaints, and less disability. Supported employment embedded within a specialty health care program for individuals with psychosis and their families showed a dose–response relationship such that attending a greater number of sessions increased the likelihood of participating in employment (16). Intervention participants also reported greater quality of life and a decrease in mental health symptoms when compared to the control group.

Finally, a German patient education program within a clinical setting for young adults with diverse chronic health care conditions (a majority were episodic) included a specific career development module (17). Intervention participants were more likely to report greater work-preparedness as well as better health-related quality of life when compared to the control group.

## Discussion

The unpredictable and dynamic nature of an episodic disability in young adulthood can contribute to early and sustained exclusion from the labor market and intermittently disrupt the pathway between employment and health. Past research has highlighted the health-related benefits attributed to promoting employment (18). This systematic review examined whether employment or income support interventions could benefit the health and well-being of young adults with episodic disabilities.

We found an absence of high-quality evidence-based employment or income support interventions for young adults living with episodic disability that focuses on health-related impacts. Only five studies were identified which met our eligibility criteria, despite a large body of research highlighting the importance of employment as a critical social determinant of health in young adults with and without disabilities (19, 20). Results underscore the need to elaborate on the health and well-being implications of employment interventions for young adults with different episodic health conditions. Most interventions identified in our review focused on young adults with mental health conditions. Findings could reflect a growing acknowledgment of the relationship between poor mental health and difficulties participating in employment among young adults (21, 22). Episodic disabilities like juvenile arthritis or inflammatory bowel disease are among the most prevalent among young adults and are characterized by variable physical symptoms (e.g., pain, fatigue, and activity limitations) and can considerably impact employment (3). Moving forward, tailored interventions should be designed to account for the employment challenges of young people living with diverse episodic health conditions.

Although we uncovered a small body of evidence, our review suggests that involvement in employment interventions could provide benefits for the health of young adults with episodic disabilities, all interventions included employment and disability-specific support components which may have explained the health-related benefits. Interventions that promote employment for young adults with episodic disabilities may benefit from providing specific training on balancing health and work demands. Additional longer-term research is needed to better understand the elements of employment interventions that may be valuable to health, as well as the reciprocal relationship between work and health outcomes that can emerge over time. Participation in supported employment offers a promising practice which may foster employment engagement and improve health. Supported employment interventions aid with finding work and they offer regular counselling and job-related training which has been previously shown to be beneficial for the employment of young adults with disabilities (23). As our findings suggest, the benefits of supported employment interventions could extend to health and well-being of young adults with episodic disabilities at the early career phase.

The fluctuating nature of episodic disabilities represents a unique challenge to sustaining employment (6). Interventions in our systematic review did not explicitly address the varying activity limitations and employment restrictions that can emerge. There is a need to study the unpredictable employment challenges related to an episodic disability and develop relevant programing tailored to those at the early career phase. Additionally, most interventions focused on helping participants find employment. There was limited focus on employment conditions, including managing jobs of different quality, access to support and job accommodations, and health and safety which may shape health outcomes. For instance, young adults with an episodic health condition may experience challenges finding full-time permanent employment that offers income and resources that are beneficial to health (16). There is a need to expand employment interventions to ensure young adults living with episodic disabilities can navigate aspects of the work environment which could pose challenges to sustained employment and also adversely impact health.

A study strength includes a rigorous systematic review methodology. A limitation of our systematic review is that we did not include qualitative studies or grey literature. Capturing broader forms of evidence can be used to elaborate on the different employment intervention that can promote health.

### Concluding remarks

Our systematic review highlights the need to elaborate on the impact employment programs can have on the health and well-being of young adults living with diverse episodic disabilities. Additional insights are needed to understand how interventions can be designed to tailor and expand employment services to young adults living with episodic health conditions to fully optimize the pathways to better health.

## Supplementary material

Supplementary material
