# Supplementary material for: Impact of employment and income support interventions on the health of young adults with episodic disability: Findings from a systematic review
Source: Scand J Work Environ Health. 2024 Feb 28;50(2):122–8. doi: 10.5271/sjweh.4133 (PMC10928491; doi:10.5271/sjweh.4133)
Supplement: Supplementary material [file SJWEH-50-122-S001.pdf]

Impact of employment and income support interventions on the health of young adults with episodic disability: Findings from a systematic review[1]

by Arif Jetha PhD,[2] Lahmea Navaratnerajah, MPH, Sebastian Kondratowski, Meagan Parmassar, MPH, Lori B Tucker, MD, FRCPC, Monique AM Gignac, PhD

1. Supplementary material
2. Correspondence to: Arif Jetha PhD, Institute for Work & Health, Suite 1800, 400 University Avenue, Toronto, ON, Canada M5G 1S5. [E-mail: ajetha@iwh.on.ca]

Table S1: Data-base specific search terms

Database 1: MEDLINE (N=5006)

Database: Ovid MEDLINE: Epub Ahead of Print, In-Process & Other Non-Indexed Citations, Ovid MEDLINE® Daily and Ovid MEDLINE® <1946-Present>
Search Strategy:
----
1 young adult/
2 adolescent?.ti,ab.
3 (college adj1 age?).ti,ab.
4 "early adult?".ti,ab.
5 "emerging adult?".ti,ab.
6 "generation y".ti,ab.
7 ("Gen Z" or "Generation Z").ti,ab.
8 ("high school$" adj1 age?).ti,ab.
9 millennial?.ti,ab.
10 "new worker?".ti,ab.
11 teen?.ti,ab.
12 teenage*.ti,ab.
13 (universit$ adj1 age?).ti,ab.
14 "young adult?".ti,ab.
15 ("young people" or "young person?").ti,ab.
16 youth.ti,ab.
17 or/1-16
18 (episodic adj2 (disease* or disabilit* or symptom* or health or condition*)).ti,ab.
19 (fluctuating adj2 (disease* or disabilit* or symptom* or health or condition*)).ti,ab.
20 (unpredictable adj2 (disease* or disabilit* or symptom* or health or condition*)).ti,ab.
21 (dynamic adj2 (disease* or disabilit* or symptom* or health or condition*)).ti,ab.
22 (recurrent adj2 (disease* or disabilit* or symptom* or health or condition*)).ti,ab.
23 (hidden adj2 (disease* or disabilit* or symptom* or health or condition*)).ti,ab.
24 (invisible adj2 (disease* or disabilit* or symptom* or health or condition*)).ti,ab.
25 Inflammatory Bowel Diseases/
26 "bowel disease*".ti,ab.
27 cancer.ti,ab.
28 Neoplasms/
29 arthritis.ti,ab.
30 Arthritis/

31	"chronic pain".ti,ab.
32	Chronic Pain/
33	"chronic disease*".ti,ab.
34	Chronic Disease/
35	Fatigue Syndrome, Chronic/
36	"chronic fatigue".ti,ab.
37	Anxiety Disorders/
38	"anxiety disorder*".ti,ab.
39	(colitis adj2 ulcerative).ti,ab.
40	Colitis, Ulcerative/
41	"Crohn's disease".ti,ab.
42	Crohn Disease/)
43	depression.ti,ab.
44	Depression/
45	Depressive Disorder/
46	dysthymia.ti,ab.
47	diabetes.ti,ab.
48	Diabetes Mellitus, Type 1/ or Diabetes Mellitus, Type 2/
49	epilepsy.ti,ab.
50	Epilepsy/
51	disability.ti,ab.
52	Fibromyalgia/
53	fibromyalgia.ti,ab.
54	HIV.ti,ab.
55	HIV/
56	Arthritis, Juvenile/
57	lupus.ti,ab.
58	Lupus Nephritis/ or Lupus Erythematosus, Systemic/
59	"mental disorder*".ti,ab.
60	mental disorders/
61	"mental health".ti,ab.
62	"mental illness".ti,ab.
63	Migraine Disorders/
64	migraine*.ti,ab.
65	"mood disorder*".ti,ab.
66	Mood Disorders/
67	"multiple sclerosis".ti,ab.
68	Multiple Sclerosis/
69	musculoskeletal disorder?.ti,ab.
70	Musculoskeletal Diseases/
71	"progressive condition*".ti,ab.
72	"psychiatric disabilit*".ti,ab.
73	"rheumatic disease*".ti,ab.
74	Rheumatic Diseases/
75	Arthritis, Rheumatoid/
76	Bipolar.ti,ab. or Bipolar Disorder/
77	Breast Neoplasms/
78	Stress Disorders, Post-Traumatic/
79	stress disorders, traumatic/
80	stress disorders, traumatic, acute/
81	post-traumatic stress.ti,ab.

82	posttraumatic stress.ti,ab.
83	(PTSD or PTSS).ti,ab.
84	(post-Covid* adj2 symptom*).ti,ab.
85	(post-Covid* adj2 syndrome*).ti,ab.
86	Spondyloarthropathy.ti,ab.
87	Spondylarthropathies/
88	Psoriasis.ti,ab.
89	Psoriasis/
90	Scleroderma.ti,ab.
91	scleroderma, Localized/ or Scleroderma, Diffuse/ or Scleroderma, Systemic/
92	Morphea.ti,ab.
93	((autoimmune adj2 condition*) or (autoimmune adj2 disease*) or (autoimmune adj2 disorder*)).ti,ab.
94	Autoimmune Diseases/
95	"premenstrual symptom*".ti,ab.
96	Premenstrual Dysphoric Disorder/
97	or/18-96
98	17 and 97
99	"work-integrated learning".ti,ab.
100	(career adj2 advice).ti,ab.
101	(career adj2 (counseling or counselling)).ti,ab.
102	("co-operative education" or "cooperative education").ti,ab.
103	"duty to accommodat*".ti,ab.
104	(accommodate or accommodation?).ti,ab.
105	(employment adj3 (ready or readiness)).ti,ab.
106	(employment adj3 support$).ti,ab.
107	(employment adj3 transition$).ti,ab.
108	"employment polic*".ti,ab.
109	"employment program$".ti,ab.
110	Vocational Guidance/
111	Rehabilitation, Vocational/
112	"vocational rehabilitation".ti,ab.
113	("school to work" adj2 transition).ti,ab.
114	"job skilling".ti,ab.
115	reskilling.ti,ab.
116	upskilling.ti,ab.
117	(work$ adj2 accessibility).ti,ab.
118	(work$ adj2 inclusion).ti,ab.
119	(employment adj2 accessibility).ti,ab.
120	(employment adj2 inclusion).ti,ab.
121	("labo?r market" adj2 (policies or policy)).ti,ab.
122	("labo?r market" adj2 participat*).ti,ab.
123	("work disability" adj2 prevention).ti,ab.
124	"labo?r market integration".ti,ab.
125	"labo?r market engagement".ti,ab.
126	"job stability".ti,ab.
127	"job search$".ti,ab.
128	"labo?r market entry".ti,ab.
129	("labo?r market" adj2 advancement).ti,ab.
130	(career adj2 advancement).ti,ab.
131	"employee intensive program$".ti,ab.

132	"job subsid$".ti,ab.
133	practicum?.ti,ab.
134	internship?.ti,ab.
135	apprenticeship$.ti,ab.
136	"job readiness".ti,ab.
137	self-employment.ti,ab.
138	social enterprise$.ti,ab.
139	start-up?.ti,ab.
140	incubator?.ti,ab.
141	"seed money".ti,ab.
142	entrepreneurship.ti,ab.
143	"small business$".ti,ab.
144	or/99-143
145	(income adj2 support$).ti,ab.
146	(disability adj2 support$).ti,ab.
147	(disability adj2 benefit*).ti,ab.
148	"employment insurance".ti,ab.
149	Social Security/
150	"social insurance".ti,ab.
151	"worker$ compensation".ti,ab.
152	Workers' Compensation/
153	Insurance, Health/
154	"health insurance".ti,ab.
155	(unemployment adj2 benefit$).ti,ab.
156	"social protection".ti,ab.
157	"income security".ti,ab.
158	(disab* adj2 benefit*).ti,ab.
159	"basic income".ti,ab.
160	UBI.ti,ab.
161	"unemployment assistan*".ti,ab.
162	workfare.ti,ab.
163	flexicurity.ti,ab.
164	"guaranteed income support".ti,ab.
165	"guaranteed minimum income".ti,ab.
166	(state adj1 benefit*).ti,ab.
167	(uncompensated adj1 care).ti,ab.
168	welfare.ti,ab.
169	(disability adj2 grant*).ti,ab.
170	"disability support".ti,ab.
171	(disability adj2 "tax credit$").ti,ab.
172	"disability savings plan?".ti,ab.
173	"medical tax credit?".ti,ab.
174	(accessibility adj2 funding).ti,ab.
175	or/145-174
176	(training adj1 skill?).ti,ab.
177	"government plan$".ti,ab.
178	(government adj1 subsid$).ti,ab.
179	(interview$ adj1 support$).ti,ab.
180	(interview$ adj1 mock).ti,ab.
181	mentoring.ti,ab.
182	(occupation$ adj1 (ready or readiness)).ti,ab.

183	(peer? adj1 support$).ti,ab.
184	"vocational guidance".ti,ab.
185	"vocational readiness".ti,ab.
186	(work adj1 accommodat$).ti,ab.
187	(employment adj2 placement).ti,ab.
188	(workplace adj2 placement).ti,ab.
189	"higher education".ti,ab.
190	"graduate education".ti,ab.
191	"postgraduate training".ti,ab.
192	"undergraduate education".ti,ab.
193	Educational Status/
194	(education$ adj1 status).ti,ab.
195	(education$ adj1 attainment).ti,ab.
196	(education$ adj1 level).ti,ab.
197	(education$ adj1 difference$).ti,ab.
198	(education adj1 inequality).ti,ab.
199	"academic achievement".ti,ab.
200	diploma?.ti,ab.
201	school graduation.ti,ab.
202	dropout.ti,ab.
203	(school adj1 retention).ti,ab.
204	"education disruption*".ti,ab.
205	scholarship$.ti,ab.
206	"transition program$".ti,ab.
207	"foundation year".ti,ab.
208	"transition* year".ti,ab.
209	(pathways adj1 education*).ti,ab.
210	(university adj1 application$).ti,ab.
211	"mature student$".ti,ab.
212	"high school equivalency".ti,ab.
213	GED.ti,ab.
214	"adult education".ti,ab.
215	"adult learning".ti,ab.
216	"continuing education".ti,ab.
217	Education, Continuing/
218	"pitch competition".ti,ab.
219	hackathon?.ti,ab.
220	"pre-entry to practice".ti,ab.
221	pushout?.ti,ab.
222	or/176-221
223	144 or 175 or 222
224	(health adj2 difference*).ti,ab.
225	(health adj2 disparit*).ti,ab.
226	(health adj2 equit*).ti,ab.
227	(self-reported adj2 health).ti,ab.
228	(self-rated adj2 health).ti,ab.
229	well-being.ti,ab.
230	"Quality of Life"/
231	"quality of life".ti,ab.
232	(activity adj2 limitation$).ti,ab.
233	impairment.ti,ab.

234 (symptom$ adj2 severity).ti,ab.
235 (disability adj2 severity).ti,ab.
236 "disease activity".ti,ab.
237 "health status".ti,ab.
238 health status/
239 (injury or injuries).ti,ab.
240 "Wounds and Injuries"/
241 (disease adj2 progress*).ti,ab.
242 (disease adj2 impact$).ti,ab.
243 or/224-242
244 (social adj2 depriv*).ti,ab.
245 (social adj2 disadvantag$).ti,ab.
246 (social adj2 equity).ti,ab.
247 (social adj2 inequity).ti,ab.
248 (social adj2 marginali#ed).ti,ab.
249 (social adj1 capital).ti,ab.
250 (social adj1 class).ti,ab.
251 (social adj2 condition$).ti,ab.
252 (social adj2 depriv*).ti,ab.
253 (social adj2 difference$).ti,ab.
254 (socio adj1 economic adj1 position).ti,ab.
255 (socio adj1 economic adj1 status).ti,ab.
256 (socio adj1 economic adj1 variable$).ti,ab.
257 (socioeconomic adj2 attribut*).ti,ab.
258 (socioeconomic adj1 circumstance$).ti,ab.
259 (socioeconomic adj1 factor$).ti,ab.
260 (socioeconomic adj1 gradient$).ti,ab.
261 (socioeconomic adj1 health adj1 difference$).ti,ab.
262 (socioeconomic adj1 position).ti,ab.
263 (socioeconomic adj1 status).ti,ab.
264 (standard adj2 living).ti,ab.
265 Socioeconomic Factors/
266 social inclusion.ti,ab.
267 financial status.ti,ab.
268 income status.ti,ab.
269 cultural capital.ti,ab.
270 cultural currency.ti,ab.
271 low income.ti,ab.
272 poverty.ti,ab.
273 Poverty/
274 social mobility.ti,ab.
275 (social adj1 accessibility).ti,ab.
276 or/244-275
277 243 or 276
278 98 and 223 and 277

Database 2: Embase (N=5184)

Database: Embase Classic+Embase <1947 to 2023 May 10>
Search Strategy:
----
1 young adult/

2	adolescent?.ti,ab.
3	(college adj1 age?).ti,ab.
4	"early adult?".ti,ab.
5	"emerging adult?".ti,ab.
6	"generation y".ti,ab. (261)
7	("Gen Z" or "Generation Z").ti,ab.
8	("high school$" adj1 age?).ti,ab.
9	millennial?.ti,ab.
10	"new worker?".ti,ab.
11	teen?.ti,ab.
12	teenage*.ti,ab.
13	(universit$ adj1 age?).ti,ab.
14	"young adult?".ti,ab.
15	("young people" or "young person?").ti,ab.
16	youth.ti,ab.
17	or/1-16
18	(episodic adj2 (disease* or disabilit* or symptom* or health or condition*)).ti,ab.
19	(fluctuating adj2 (disease* or disabilit* or symptom* or health or condition*)).ti,ab.
20	(unpredictable adj2 (disease* or disabilit* or symptom* or health or condition*)).ti,ab.
21	(dynamic adj2 (disease* or disabilit* or symptom* or health or condition*)).ti,ab.
22	(recurrent adj2 (disease* or disabilit* or symptom* or health or condition*)).ti,ab.
23	(hidden adj2 (disease* or disabilit* or symptom* or health or condition*)).ti,ab.
24	(hidden adj2 (disease* or disabilit* or symptom* or health or condition*)).ti,ab.
25	(invisible adj2 (disease* or disabilit* or symptom* or health or condition*)).ti,ab.
26	inflammatory bowel disease/
27	"bowel disease*".ti,ab.
28	cancer.ti,ab.
29	malignant neoplasm/
30	arthritis.ti,ab.
31	arthritis/
32	"chronic pain".ti,ab.
33	chronic pain/
34	"chronic disease".ti,ab.
35	chronic disease/
36	"chronic fatigue".ti,ab.
37	chronic fatigue syndrome/
38	anxiety disorder/
39	"anxiety disorder*".ti,ab.
40	(colitis adj2 ulcerative).ti,ab.
41	ulcerative colitis/
42	"Crohn's disease".ti,ab.
43	Crohn disease/
44	depression.ti,ab.
45	depression/
46	dysthymia.ti,ab.
47	diabetes.ti,ab.
48	diabetes mellitus/
49	epilepsy.ti,ab.
50	epilepsy/
51	disability.ti,ab.
52	fibromyalgia/

53 fibromyalgia.ti,ab.
54 HIV.ti,ab.
55 HIV/
56 juvenile rheumatoid arthritis/
57 lupus.ti,ab.
58 lupus erythematosus nephritis/
59 "mental disorder*".ti,ab.
60 mental disease/
61 "mental health".ti,ab.
62 "mental illness".ti,ab.
63 migraine/
64 migraine*.ti,ab.
65 "mood disorder*".ti,ab.
66 mood disorder/
67 "multiple sclerosis".ti,ab.
68 multiple sclerosis/
69 musculoskeletal disorder?.ti,ab.
70 musculoskeletal disease/
71 "progressive condition*".ti,ab.
72 "psychiatric disabilit*".ti,ab.
73 "rheumatic disease*".ti,ab.
74 rheumatic disease/
75 rheumatoid arthritis/
76 bipolar.ti,ab.
77 bipolar disorder/
78 breast cancer/
79 posttraumatic stress disorder/
80 post-traumatic stress.ti,ab.
81 posttraumatic stress.ti,ab.
82 (PTSD or PTSS).ti,ab.
83 (post-Covid* adj2 symptom*).ti,ab.
84 (post-Covid* adj2 syndrome*).ti,ab.
85 Spondyloarthropathy.ti,ab.
86 spondyloarthropathy/
87 psoriasis.ti,ab.
88 psoriasis/
89 scleroderma.ti,ab.
90 scleroderma/
91 morphea.ti,ab.
92 ((autoimmune adj2 condition*) or (autoimmune adj2 disease*) or (autoimmune adj2 disorder*)).ti,ab.
93 autoimmune disease/
94 "premenstrual symptom*".ti,ab.
95 premenstrual dysphoric disorder/
96 or/18-95
97 17 and 96
98 "work-integrated learning".ti,ab.
99 (career adj2 advice).ti,ab.
100 (career adj2 (counseling or counselling)).ti,ab.
101 ("co-operative education" or "cooperative education").ti,ab.
102 "duty to accommodat*".ti,ab.

103	(accommodate or accommodation?).ti,ab.
104	(employment adj3 (ready or readiness)).ti,ab.
105	(employment adj3 support$).ti,ab.
106	(employment adj3 transition$).ti,ab.
107	"employment polic*".ti,ab.
108	"employment program$".ti,ab.
109	vocational guidance/
110	vocational rehabilitation/
111	"vocational rehabilitation".ti,ab.
112	("school to work" adj2 transition).ti,ab.
113	"job skilling".ti,ab.
114	reskilling.ti,ab.
115	upskilling.ti,ab.
116	(work$ adj2 accessibility).ti,ab.
117	(work$ adj2 inclusion).ti,ab.
118	(employment adj2 accessibility).ti,ab.
119	(employment adj2 inclusion).ti,ab.
120	("labo?r market" adj2 (policies or policy)).ti,ab.
121	("labo?r market" adj2 participat*).ti,ab.
122	("work disability" adj2 prevention).ti,ab.
123	"labo?r market integration".ti,ab.
124	"labo?r market engagement".ti,ab.
125	"job stability".ti,ab.
126	"job search$".ti,ab.
127	"labo?r market entry".ti,ab.
128	("labo?r market" adj2 advancement).ti,ab.
129	(career adj2 advancement).ti,ab.
130	"employee intensive program$".ti,ab.
131	"job subsid$".ti,ab.
132	practicum?.ti,ab.
133	internship?.ti,ab.
134	apprenticeship$.ti,ab.
135	"job readiness".ti,ab.
136	self-employment.ti,ab.
137	social enterprise$.ti,ab.
138	start-up?.ti,ab.
139	incubator?.ti,ab.
140	"seed money".ti,ab.
141	entrepreneurship.ti,ab.
142	"small business$".ti,ab.
143	or/98-142
144	(income adj2 support$).ti,ab.
145	(disability adj2 support$).ti,ab.
146	(disability adj2 benefit*).ti,ab.
147	"employment insurance".ti,ab.
148	social security/
149	"social insurance".ti,ab.
150	"worker$ compensation".ti,ab.
151	workman compensation/
152	health insurance/
153	"health insurance".ti,ab.

154	(unemployment adj2 benefit$).ti,ab.
155	"social protection".ti,ab.
156	"income security".ti,ab.
157	(disab* adj2 benefit*).ti,ab.
158	"basic income".ti,ab.
159	UBI.ti,ab.
160	"unemployment assistan*".ti,ab.
161	workfare.ti,ab.
162	flexicurity.ti,ab.
163	"guaranteed income support".ti,ab.
164	"guaranteed minimum income".ti,ab.
165	(state adj1 benefit*).ti,ab.
166	(uncompensated adj1 care).ti,ab.
167	welfare.ti,ab.
168	(disability adj2 grant*).ti,ab.
169	"disability support".ti,ab.
170	(disability adj2 "tax credit$").ti,ab.
171	"disability savings plan?".ti,ab.
172	"medical tax credit?".ti,ab.
173	(accessibility adj2 funding).ti,ab.
174	or/144-173
175	(training adj1 skill?).ti,ab.
176	"government plan$".ti,ab.
177	(government adj1 subsid$).ti,ab.
178	(interview$ adj1 support$).ti,ab.
179	(interview$ adj1 mock).ti,ab.
180	mentoring.ti,ab.
181	(occupation$ adj1 (ready or readiness)).ti,ab.
182	(peer? adj1 support$).ti,ab.
183	"vocational guidance".ti,ab.
184	"vocational readiness".ti,ab.
185	(work adj1 accommodat$).ti,ab.
186	(employment adj2 placement).ti,ab.
187	(workplace adj2 placement).ti,ab.
188	"higher education".ti,ab.
189	"graduate education".ti,ab.
190	"postgraduate training".ti,ab.
191	"undergraduate education".ti,ab.
192	educational status/
193	(education$ adj1 status).ti,ab.
194	(education$ adj1 attainment).ti,ab.
195	(education$ adj1 level).ti,ab.
196	(education$ adj1 difference$).ti,ab.
197	(education adj1 inequality).ti,ab.
198	"academic achievement".ti,ab.
199	diploma?.ti,ab.
200	school graduation.ti,ab.
201	dropout.ti,ab.
202	(school adj1 retention).ti,ab.
203	"education disruption*".ti,ab.
204	scholarship$.ti,ab.

205	"transition program$".ti,ab.
206	"foundation year".ti,ab.
207	"transition* year".ti,ab.
208	(pathways adj1 education*).ti,ab.
209	(university adj1 application$).ti,ab.
210	"mature student$".ti,ab.
211	"high school equivalency".ti,ab.
212	GED.ti,ab.
213	"adult education".ti,ab.
214	"adult learning".ti,ab.
215	"continuing education".ti,ab.
216	continuing education/
217	"pitch competition".ti,ab.
218	hackathon?.ti,ab.
219	"pre-entry to practice".ti,ab.
220	pushout?.ti,ab.
221	or/175-220
222	143 or 174 or 221
223	(health adj2 difference*).ti,ab.
224	(health adj2 disparit*).ti,ab.
225	(health adj2 equit*).ti,ab.
226	(self-reported adj2 health).ti,ab.
227	(self-rated adj2 health).ti,ab.
228	well-being.ti,ab.
229	"quality of life"/
230	"quality of life".ti,ab.
231	(activity adj2 limitation$).ti,ab.
232	impairment.ti,ab.
233	(symptom$ adj2 severity).ti,ab.
234	(disability adj2 severity).ti,ab.
235	"disease activity".ti,ab.
236	"health status".ti,ab.
237	health status/
238	(injury or injuries).ti,ab.
239	injury/
240	(injury or injuries).ti,ab.
241	(disease adj2 progress*).ti,ab.
242	(disease adj2 impact$).ti,ab.
243	or/223-242
244	(social adj2 depriv*).ti,ab.
245	(social adj2 disadvantag$).ti,ab.
246	(social adj2 equity).ti,ab.
247	(social adj2 inequity).ti,ab.
248	(social adj2 marginali#ed).ti,ab.
249	(social adj1 capital).ti,ab.
250	(social adj1 class).ti,ab.
251	(social adj2 condition$).ti,ab.
252	(social adj2 depriv*).ti,ab.
253	(social adj2 difference$).ti,ab.
254	(socio adj1 economic adj1 position).ti,ab.
255	(socio adj1 economic adj1 status).ti,ab.

256 (socio adj1 economic adj1 variable$).ti,ab.
257 (socioeconomic adj2 attribut*).ti,ab.
258 (socioeconomic adj1 circumstance$).ti,ab.
259 (socioeconomic adj1 factor$).ti,ab.
260 (socioeconomic adj1 gradient$).ti,ab.
261 (socioeconomic adj1 health adj1 difference$).ti,ab.
262 (socioeconomic adj1 position).ti,ab.
263 (socioeconomic adj1 status).ti,ab.
264 (standard adj2 living).ti,ab.
265 socioeconomics/
266 social inclusion.ti,ab.
267 financial status.ti,ab.
268 income status.ti,ab.
269 cultural capital.ti,ab.
270 cultural currency.ti,ab.
271 low income.ti,ab.
272 poverty.ti,ab.
273 poverty/
274 social mobility.ti,ab.
275 (social adj1 accessibility).ti,ab.
276 or/244-275
277 243 or 276
278 97 and 222 and 277

Database 3: PsycINFO (N=1556)

Database: APA PsycInfo <2002 to May Week 1 2023>
Search Strategy:

----

1 Emerging Adulthood/
2 adolescent?.ti,ab.
3 (college adj1 age?).ti,ab.
4 "early adult?".ti,ab.
5 "emerging adult?".ti,ab.
6 "generation y".ti,ab.
7 ("Gen Z" or "Generation Z").ti,ab.
8 ("high school$" adj1 age?).ti,ab.
9 millennial?.ti,ab.
10 "new worker?".ti,ab.
11 teen?.ti,ab.
12 teenage*.ti,ab.
13 (universit$ adj1 age?).ti,ab.
14 "young adult?".ti,ab.
15 ("young people" or "young person?").ti,ab.
16 youth.ti,ab.
17 or/1-16
18 (episodic adj2 (disease* or disabilit* or symptom* or health or condition*)).ti,ab.
19 (fluctuating adj2 (disease* or disabilit* or symptom* or health or condition*)).ti,ab.
20 (unpredictable adj2 (disease* or disabilit* or symptom* or health or condition*)).ti,ab.
21 (dynamic adj2 (disease* or disabilit* or symptom* or health or condition*)).ti,ab.
22 (recurrent adj2 (disease* or disabilit* or symptom* or health or condition*)).ti,ab.

23	(hidden adj2 (disease* or disabilit* or symptom* or health or condition*)).ti,ab.
24	(invisible adj2 (disease* or disabilit* or symptom* or health or condition*)).ti,ab.
25	Irritable Bowel Syndrome/
26	"bowel disease*".ti,ab.
27	cancer.ti,ab.
28	Neoplasms/
29	arthritis.ti,ab.
30	Arthritis/
31	"chronic pain".ti,ab.
32	Chronic Pain/
33	"chronic disease*".ti,ab.
34	Chronic Illness/
35	Chronic Fatigue Syndrome/
36	"chronic fatigue".ti,ab.
37	Anxiety Disorders/
38	"anxiety disorder*".ti,ab.
39	(colitis adj2 ulcerative).ti,ab.
40	Ulcerative Colitis/
41	"Crohn's disease".ti,ab.
42	depression.ti,ab.
43	Major Depression/ or Recurrent Depression/
44	dysthymia.ti,ab.
45	diabetes.ti,ab.
46	Diabetes/
47	epilepsy.ti,ab.
48	Epilepsy/
49	disability.ti,ab.
50	fibromyalgia.ti,ab.
51	Fibromyalgia/
52	HIV.ti,ab.
53	HIV/
54	lupus.ti,ab.
55	Lupus/
56	"mental disorder*".ti,ab.
57	Mental Disorders/
58	"mental health".ti,ab.
59	"mental illness".ti,ab.
60	migraine*.ti,ab.
61	Migraine Headache/
62	"mood disorder*".ti,ab.
63	Affective Disorders/
64	"multiple sclerosis".ti,ab.
65	Multiple Sclerosis/
66	musculoskeletal disorder?.ti,ab.
67	Musculoskeletal Disorders/
68	"progressive condition*".ti,ab.
69	"psychiatric disabilit*".ti,ab.
70	"rheumatic disease*".ti,ab.
71	Rheumatoid Arthritis/
72	bipolar.ti,ab.
73	Bipolar Disorder/

74 Breast Neoplasms/
75 Breast Neoplasms/
76 Posttraumatic Stress Disorder/
77 posttraumatic stress.ti,ab.
78 post-traumatic stress.ti,ab.
79 (PTSD or PTSS).ti,ab.
80 (post-Covid* adj2 symptom*).ti,ab.
81 (post-Covid* adj2 syndrome*).ti,ab.
82 Spondyloarthropathy.ti,ab.
83 psoriasis.ti,ab.
84 Skin Disorders/
85 Scleroderma.ti,ab.
86 morphea.ti,ab.
87 ((autoimmune adj2 condition*) or (autoimmune adj2 disease*) or (autoimmune adj2 disorder*)).ti,ab.
88 Immunologic Disorders/
89 "premenstrual symptom*".ti,ab.
90 Premenstrual Dysphoric Disorder/
91 or/18-90
92 17 and 91
93 "work-integrated learning".ti,ab.
94 (career adj2 advice).ti,ab.
95 (career adj2 (counseling or counselling)).ti,ab.
96 ("co-operative education" or "cooperative education").ti,ab.
97 "duty to accommodat*".ti,ab.
98 (accommodate or accommodation?).ti,ab.
99 (employment adj3 (ready or readiness)).ti,ab.
100 (employment adj3 support$).ti,ab.
101 (employment adj3 transition$).ti,ab.
102 "employment polic*".ti,ab.
103 "employment program$".ti,ab.
104 Occupational Guidance/
105 Vocational Rehabilitation/
106 "vocational rehabilitation".ti,ab.
107 ("school to work" adj2 transition).ti,ab.
108 "job skilling".ti,ab.
109 reskilling.ti,ab.
110 upskilling.ti,ab.
111 (work$ adj2 accessibility).ti,ab.
112 (work$ adj2 inclusion).ti,ab.
113 (employment adj2 accessibility).ti,ab.
114 (employment adj2 inclusion).ti,ab.
115 ("labo?r market" adj2 (policies or policy)).ti,ab.
116 ("labo?r market" adj2 participat*).ti,ab.
117 ("work disability" adj2 prevention).ti,ab.
118 "labo?r market integration".ti,ab.
119 "labo?r market engagement".ti,ab.
120 "job stability".ti,ab.
121 "job search$".ti,ab.
122 "labo?r market entry".ti,ab.
123 ("labo?r market" adj2 advancement).ti,ab.

124	(career adj2 advancement).ti,ab.
125	"employee intensive program$".ti,ab.
126	"job subsid$".ti,ab.
127	practicum?.ti,ab.
128	internship?.ti,ab.
129	apprenticeship$.ti,ab.
130	"job readiness".ti,ab.
131	self-employment.ti,ab.
132	social enterprise$.ti,ab.
133	start-up?.ti,ab.
134	incubator?.ti,ab.
135	"seed money".ti,ab.
136	entrepreneurship.ti,ab.
137	"small business$".ti,ab.
138	or/93-137
139	(income adj2 support$).ti,ab.
140	(disability adj2 support$).ti,ab.
141	(disability adj2 benefit*).ti,ab.
142	"employment insurance".ti,ab.
143	Social Security/
144	"social insurance".ti,ab.
145	"worker$ compensation".ti,ab.
146	Workers' Compensation Insurance/
147	Health Insurance/
148	"health insurance".ti,ab.
149	(unemployment adj2 benefit$).ti,ab.
150	"social protection".ti,ab.
151	"income security".ti,ab.
152	(disab* adj2 benefit*).ti,ab.
153	"basic income".ti,ab.
154	UBI.ti,ab.
155	"unemployment assistan*".ti,ab.
156	workfare.ti,ab.
157	flexicurity.ti,ab.
158	"guaranteed income support".ti,ab.
159	"guaranteed minimum income".ti,ab.
160	(state adj1 benefit*).ti,ab.
161	(uncompensated adj1 care).ti,ab.
162	welfare.ti,ab.
163	(disability adj2 grant*).ti,ab.
164	"disability support".ti,ab.
165	(disability adj2 "tax credit$").ti,ab.
166	"disability savings plan?".ti,ab.
167	"medical tax credit?".ti,ab.
168	(accessibility adj2 funding).ti,ab.
169	or/139-168
170	(training adj1 skill?).ti,ab.
171	"government plan$".ti,ab.
172	(government adj1 subsid$).ti,ab.
173	(interview$ adj1 support$).ti,ab.
174	(interview$ adj1 mock).ti,ab.

175	mentoring.ti,ab.
176	(occupation$ adj1 (ready or readiness)).ti,ab.
177	(peer? adj1 support$).ti,ab.
178	"vocational guidance".ti,ab.
179	"vocational readiness".ti,ab.
180	(work adj1 accommodat$).ti,ab.
181	(employment adj2 placement).ti,ab.
182	(workplace adj2 placement).ti,ab.
183	"higher education".ti,ab.
184	"graduate education".ti,ab.
185	"postgraduate training".ti,ab.
186	"undergraduate education".ti,ab.
187	Educational Attainment Level/
188	(education$ adj1 status).ti,ab.
189	(education$ adj1 attainment).ti,ab.
190	(education$ adj1 level).ti,ab.
191	(education$ adj1 difference$).ti,ab.
192	(education adj1 inequality).ti,ab.
193	"academic achievement".ti,ab.
194	diploma?.ti,ab.
195	school graduation.ti,ab.
196	dropout.ti,ab.
197	(school adj1 retention).ti,ab.
198	"education disruption*".ti,ab.
199	scholarship$.ti,ab.
200	"transition program$".ti,ab.
201	"foundation year".ti,ab.
202	"transition* year".ti,ab.
203	(pathways adj1 education*).ti,ab.
204	(university adj1 application$).ti,ab.
205	"mature student$".ti,ab.
206	"high school equivalency".ti,ab.
207	GED.ti,ab.
208	"adult education".ti,ab.
209	"adult learning".ti,ab.
210	"continuing education".ti,ab.
211	Continuing Education/
212	"pitch competition".ti,ab.
213	hackathon?.ti,ab.
214	"pre-entry to practice".ti,ab.
215	pushout?.ti,ab.
216	or/170-215
217	138 or 169 or 216
218	(health adj2 difference*).ti,ab.
219	(health adj2 disparit*).ti,ab.
220	(health adj2 equit*).ti,ab.
221	(self-reported adj2 health).ti,ab.
222	(self-rated adj2 health).ti,ab.
223	well-being.ti,ab.
224	"Quality of Life"/
225	"quality of life".ti,ab.

226	(activity adj2 limitation$).ti,ab.
227	impairment.ti,ab.
228	(symptom$ adj2 severity).ti,ab.
229	(disability adj2 severity).ti,ab.
230	"disease activity".ti,ab.
231	"health status".ti,ab.
232	Health Status/
233	(injury or injuries).ti,ab.
234	Injuries/
235	(disease adj2 progress*).ti,ab.
236	(disease adj2 impact$).ti,ab.
237	or/218-236
238	(social adj2 depriv*).ti,ab.
239	(social adj2 disadvantag$).ti,ab.
240	(social adj2 equity).ti,ab.
241	(social adj2 inequity).ti,ab.
242	(social adj2 marginali#ed).ti,ab.
243	(social adj1 capital).ti,ab.
244	(social adj1 class).ti,ab.
245	(social adj2 condition$).ti,ab.
246	(social adj2 depriv*).ti,ab.
247	(social adj2 difference$).ti,ab.
248	(socio adj1 economic adj1 position).ti,ab.
249	(socio adj1 economic adj1 status).ti,ab.
250	(socio adj1 economic adj1 variable$).ti,ab.
251	(socioeconomic adj2 attribut*).ti,ab.
252	(socioeconomic adj1 circumstance$).ti,ab.
253	(socioeconomic adj1 factor$).ti,ab.
254	(socioeconomic adj1 gradient$).ti,ab.
255	(socioeconomic adj1 health adj1 difference$).ti,ab.
256	(socioeconomic adj1 position).ti,ab.
257	(socioeconomic adj1 status).ti,ab.
258	(standard adj2 living).ti,ab.
259	Socioeconomic Factors/
260	social inclusion.ti,ab.
261	financial status.ti,ab.
262	income status.ti,ab.
263	cultural capital.ti,ab.
264	cultural currency.ti,ab.
265	low income.ti,ab.
266	poverty.ti,ab.
267	Poverty/
268	social mobility.ti,ab.
269	(social adj1 accessibility).ti,ab.
270	or/238-269
271	237 or 270
272	92 and 217 and 271

Database 4: CINAHL (N=2459)

S259 S92 AND S205 AND S258 Search modes - Boolean/Phrase Interface - EBSCOhost Research Databases
Search Screen - Advanced Search
Database - CINAHL Plus with Full Text Display
S258 S226 OR S257 Search modes - Boolean/Phrase Interface - EBSCOhost Research Databases
Search Screen - Advanced Search
Database - CINAHL Plus with Full Text Display
S257 S227 OR S228 OR S229 OR S230 OR S231 OR S232 OR S233 OR S234 OR S235 OR S236 OR S237 OR S238 OR S239 OR S240 OR S241 OR S242 OR S243 OR S244 OR S245 OR S246 OR S247 OR S248 OR S249 OR S250 OR S251 OR S252 OR S253 OR S254 OR S255 OR S256 Search modes - Boolean/Phrase Interface - EBSCOhost Research Databases
Search Screen - Advanced Search
Database - CINAHL Plus with Full Text Display
S256 TI (social N1 accessibility) AND AB (social N1 accessibility) Search modes - Boolean/Phrase Interface - EBSCOhost Research Databases
Search Screen - Advanced Search
Database - CINAHL Plus with Full Text Display
S255 TI "social mobility" AND AB "social mobility" Search modes - Boolean/Phrase Interface - EBSCOhost Research Databases
Search Screen - Advanced Search
Database - CINAHL Plus with Full Text Display
S254 TI "low income" OR AB "low income" Search modes - Boolean/Phrase Interface - EBSCOhost Research Databases
Search Screen - Advanced Search
Database - CINAHL Plus with Full Text Display
S253 TI "cultural currency" OR AB "cultural currency" Search modes - Boolean/Phrase Interface - EBSCOhost Research Databases
Search Screen - Advanced Search
Database - CINAHL Plus with Full Text Display
S252 TI "cultural capital" OR AB "cultural capital" Search modes - Boolean/Phrase Interface - EBSCOhost Research Databases
Search Screen - Advanced Search
Database - CINAHL Plus with Full Text Display
S251 TI "financial status" OR AB "financial status" Search modes - Boolean/Phrase Interface - EBSCOhost Research Databases
Search Screen - Advanced Search
Database - CINAHL Plus with Full Text Display
S250 TI "social inclusion" OR AB "social inclusion" Search modes - Boolean/Phrase Interface - EBSCOhost Research Databases
Search Screen - Advanced Search
Database - CINAHL Plus with Full Text Display
S249 (MH "Socioeconomic Factors") Search modes - Boolean/Phrase Interface - EBSCOhost Research Databases
Search Screen - Advanced Search
Database - CINAHL Plus with Full Text Display
S248 TI (standard N2 living) OR AB (standard N2 living) Search modes - Boolean/Phrase Interface - EBSCOhost Research Databases
Search Screen - Advanced Search
Database - CINAHL Plus with Full Text Display
S247 TI (socioeconomic N1 status) OR AB (socioeconomic N1 status) Search modes - Boolean/Phrase Interface - EBSCOhost Research Databases

Search Screen - Advanced Search
Database - CINAHL Plus with Full Text Display
S246 TI (socioeconomic N1 position#) OR AB (socioeconomic N1 position#) Search modes - Boolean/Phrase Interface - EBSCOhost Research Databases
Search Screen - Advanced Search
Database - CINAHL Plus with Full Text Display
S245 TI (socioeconomic N1 health N1 difference#) OR AB (socioeconomic N1 health N1 difference#) Search modes - Boolean/Phrase Interface - EBSCOhost Research Databases
Search Screen - Advanced Search
Database - CINAHL Plus with Full Text Display
S244 TI (socioeconomic N1 gradient#) OR AB (socioeconomic N1 gradient#) Search modes - Boolean/Phrase Interface - EBSCOhost Research Databases
Search Screen - Advanced Search
Database - CINAHL Plus with Full Text Display
S243 TI (socioeconomic N1 factor*) OR AB (socioeconomic N1 factor*) Search modes - Boolean/Phrase Interface - EBSCOhost Research Databases
Search Screen - Advanced Search
Database - CINAHL Plus with Full Text Display
S242 TI (socioeconomic N1 circumstance*) OR AB (socioeconomic N1 circumstance*) Search modes - Boolean/Phrase Interface - EBSCOhost Research Databases
Search Screen - Advanced Search
Database - CINAHL Plus with Full Text Display
S241 TI (socioeconomic N2 circumstance*) OR AB (socioeconomic N1 circumstance*) Search modes - Boolean/Phrase Interface - EBSCOhost Research Databases
Search Screen - Advanced Search
Database - CINAHL Plus with Full Text Display
S240 TI (socioeconomic N2 attribut*) OR AB (socioeconomic N2 attribut*) Search modes - Boolean/Phrase Interface - EBSCOhost Research Databases
Search Screen - Advanced Search
Database - CINAHL Plus with Full Text Display
S239 TI (socio N1 economic N1 variable#) OR AB (socio N1 economic N1 variable#) Search modes - Boolean/Phrase Interface - EBSCOhost Research Databases
Search Screen - Advanced Search
Database - CINAHL Plus with Full Text Display
S238 TI (socio N1 economic N1 status) OR AB (socio N1 economic N1 status) Search modes - Boolean/Phrase Interface - EBSCOhost Research Databases
Search Screen - Advanced Search
Database - CINAHL Plus with Full Text Display
S237 TI (socio N1 economic N1 position) OR AB (socio N1 economic N1 position) Search modes - Boolean/Phrase Interface - EBSCOhost Research Databases
Search Screen - Advanced Search
Database - CINAHL Plus with Full Text Display
S236 TI (social n2 difference*) OR AB (social n2 difference*) Search modes - Boolean/Phrase Interface - EBSCOhost Research Databases
Search Screen - Advanced Search
Database - CINAHL Plus with Full Text Display
S235 TI (social n2 depriv*) OR AB (social n2 depriv*) Search modes - Boolean/Phrase Interface - EBSCOhost Research Databases
Search Screen - Advanced Search
Database - CINAHL Plus with Full Text Display

S234 TI (social n2 condition#) OR AB (social n2 condition#) Search modes - Boolean/Phrase Interface - EBSCOhost Research Databases
Search Screen - Advanced Search
Database - CINAHL Plus with Full Text Display
S233 TI (social n2 class) OR AB (social n2 class) Search modes - Boolean/Phrase Interface - EBSCOhost Research Databases
Search Screen - Advanced Search
Database - CINAHL Plus with Full Text Display
S232 TI (social n2 capital) OR AB (social n2 capital) Search modes - Boolean/Phrase Interface - EBSCOhost Research Databases
Search Screen - Advanced Search
Database - CINAHL Plus with Full Text Display
S231 TI (social n2 marginali#ed) OR AB (social n2 marginali#ed) Search modes - Boolean/Phrase Interface - EBSCOhost Research Databases
Search Screen - Advanced Search
Database - CINAHL Plus with Full Text Display
S230 TI (social n2 inequity) OR AB (social n2 inequity) Search modes - Boolean/Phrase Interface - EBSCOhost Research Databases
Search Screen - Advanced Search
Database - CINAHL Plus with Full Text Display
S229 TI (social n2 equity) OR AB (social n2 equity) Search modes - Boolean/Phrase Interface - EBSCOhost Research Databases
Search Screen - Advanced Search
Database - CINAHL Plus with Full Text Display
S228 TI (social n2 disadvantag*) OR AB (social n2 disadvantag*) Search modes - Boolean/Phrase Interface - EBSCOhost Research Databases
Search Screen - Advanced Search
Database - CINAHL Plus with Full Text Display
S227 TI (social n2 depriv*) OR AB (social n2 depriv*) Search modes - Boolean/Phrase Interface - EBSCOhost Research Databases
Search Screen - Advanced Search
Database - CINAHL Plus with Full Text Display
S226 S206 OR S207 OR S208 OR S209 OR S210 OR S211 OR S212 OR S213 OR S214 OR S215 OR S216 OR S217 OR S218 OR S219 OR S220 OR S221 OR S222 OR S223 OR S224 OR S225 Search modes - Boolean/Phrase Interface - EBSCOhost Research Databases
Search Screen - Advanced Search
Database - CINAHL Plus with Full Text Display
S225 TI (disease N2 impact*) OR AB (disease N2 impact*) Search modes - Boolean/Phrase Interface - EBSCOhost Research Databases
Search Screen - Advanced Search
Database - CINAHL Plus with Full Text Display
S224 TI (disease N2 progress*) OR AB (disease N2 progress*) Search modes - Boolean/Phrase Interface - EBSCOhost Research Databases
Search Screen - Advanced Search
Database - CINAHL Plus with Full Text Display
S223 (MH "Wounds and Injuries") Search modes - Boolean/Phrase Interface - EBSCOhost Research Databases
Search Screen - Advanced Search
Database - CINAHL Plus with Full Text Display
S222 (TI (injury or injuries)) OR (AB (injury or injuries)) Search modes - Boolean/Phrase Interface - EBSCOhost Research Databases

Search Screen - Advanced Search
Database - CINAHL Plus with Full Text Display
S221 (MH "Health Status") Search modes - Boolean/Phrase Interface - EBSCOhost Research Databases
Search Screen - Advanced Search
Database - CINAHL Plus with Full Text Display
S220 TI "health status" OR AB "health status" Search modes - Boolean/Phrase Interface - EBSCOhost Research Databases
Search Screen - Advanced Search
Database - CINAHL Plus with Full Text Display
S219 TI "disease activity" OR AB "disease activity" Search modes - Boolean/Phrase Interface - EBSCOhost Research Databases
Search Screen - Advanced Search
Database - CINAHL Plus with Full Text Display
S218 TI (disability N2 severity) OR AB (disability N2 severity) Search modes - Boolean/Phrase Interface - EBSCOhost Research Databases
Search Screen - Advanced Search
Database - CINAHL Plus with Full Text Display
S217 TI (symptom# N2 severity) OR AB (symptom# N2 severity) Search modes - Boolean/Phrase Interface - EBSCOhost Research Databases
Search Screen - Advanced Search
Database - CINAHL Plus with Full Text Display
S216 TI impairment OR AB impairment Search modes - Boolean/Phrase Interface - EBSCOhost Research Databases
Search Screen - Advanced Search
Database - CINAHL Plus with Full Text Display
S215 TI (activity N2 limitation#) OR AB (activity N2 limitation#) Search modes - Boolean/Phrase Interface - EBSCOhost Research Databases
Search Screen - Advanced Search
Database - CINAHL Plus with Full Text Display
S214 TI (activity N2 limitation#) OR AB (activity N2 limitation#) Search modes - Boolean/Phrase Interface - EBSCOhost Research Databases
Search Screen - Advanced Search
Database - CINAHL Plus with Full Text Display
S213 (MH "Quality of Life") Search modes - Boolean/Phrase Interface - EBSCOhost Research Databases
Search Screen - Advanced Search
Database - CINAHL Plus with Full Text Display
S212 TI "quality of life" OR AB "quality of life" Search modes - Boolean/Phrase Interface - EBSCOhost Research Databases
Search Screen - Advanced Search
Database - CINAHL Plus with Full Text Display
S211 TI well-being OR AB well-being Search modes - Boolean/Phrase Interface - EBSCOhost Research Databases
Search Screen - Advanced Search
Database - CINAHL Plus with Full Text Display
S210 TI (self-rated N2 health) OR AB (self-rated N2 health) Search modes - Boolean/Phrase Interface - EBSCOhost Research Databases
Search Screen - Advanced Search
Database - CINAHL Plus with Full Text Display

S209 TI (self-reported N2 health) OR AB (self-reported N2 health) Search modes - Boolean/Phrase Interface - EBSCOhost Research Databases
Search Screen - Advanced Search
Database - CINAHL Plus with Full Text Display
S208 TI (self-reported N2 health) OR AB (self-reported N2 health) Search modes - Boolean/Phrase Interface - EBSCOhost Research Databases
Search Screen - Advanced Search
Database - CINAHL Plus with Full Text Display
S207 TI (health N2 equit*) OR AB (health N2 equit*) Search modes - Boolean/Phrase Interface - EBSCOhost Research Databases
Search Screen - Advanced Search
Database - CINAHL Plus with Full Text Display
S206 TI (health N2 difference*) OR AB (health N2 difference*) Search modes - Boolean/Phrase Interface - EBSCOhost Research Databases
Search Screen - Advanced Search
Database - CINAHL Plus with Full Text Display
S205 S132 OR S161 OR S204 Search modes - Boolean/Phrase Interface - EBSCOhost Research Databases
Search Screen - Advanced Search
Database - CINAHL Plus with Full Text Display
S204 S162 OR S163 OR S164 OR S165 OR S166 OR S167 OR S168 OR S169 OR S170 OR S171 OR S172 OR S173 OR S174 OR S175 OR S176 OR S177 OR S178 OR S179 OR S180 OR S181 OR S182 OR S183 OR S184 OR S185 OR S186 OR S187 OR S188 OR S189 OR S190 OR S191 OR S192 OR S193 OR S194 OR S195 OR S196 OR S197 OR S198 OR S199 OR S200 OR S201 OR S202 OR S203 Search modes - Boolean/Phrase Interface - EBSCOhost Research Databases
Search Screen - Advanced Search
Database - CINAHL Plus with Full Text Display
S203 TI pushout# OR AB pushout# Search modes - Boolean/Phrase Interface - EBSCOhost Research Databases
Search Screen - Advanced Search
Database - CINAHL Plus with Full Text Display
S202 TI "pre-entry to practice" OR AB "pre-entry to practice" Search modes - Boolean/Phrase Interface - EBSCOhost Research Databases
Search Screen - Advanced Search
Database - CINAHL Plus with Full Text Display
S201 TI hackathon# OR AB hackathon# Search modes - Boolean/Phrase Interface - EBSCOhost Research Databases
Search Screen - Advanced Search
Database - CINAHL Plus with Full Text Display
S200 TI "pitch competition" OR AB "pitch competition" Search modes - Boolean/Phrase Interface - EBSCOhost Research Databases
Search Screen - Advanced Search
Database - CINAHL Plus with Full Text Display
S199 TI "continuing education" OR AB "continuing education" Search modes - Boolean/Phrase Interface - EBSCOhost Research Databases
Search Screen - Advanced Search
Database - CINAHL Plus with Full Text Display
S198 TI "adult learning" OR AB "adult learning" Search modes - Boolean/Phrase Interface - EBSCOhost Research Databases
Search Screen - Advanced Search
Database - CINAHL Plus with Full Text Display

S197 TI "adult education" OR AB "adult education" Search modes - Boolean/Phrase Interface - EBSCOhost Research Databases
Search Screen - Advanced Search
Database - CINAHL Plus with Full Text Display
S196 TI GED OR AB GED Search modes - Boolean/Phrase Interface - EBSCOhost Research Databases
Search Screen - Advanced Search
Database - CINAHL Plus with Full Text Display
S195 TI "high school equivalency" OR AB "high school equivalency" Search modes - Boolean/Phrase Interface - EBSCOhost Research Databases
Search Screen - Advanced Search
Database - CINAHL Plus with Full Text Display
S194 TI "mature student#" OR AB "mature student#" Search modes - Boolean/Phrase Interface - EBSCOhost Research Databases
Search Screen - Advanced Search
Database - CINAHL Plus with Full Text Display
S193 TI (university N1 application*) OR AB (university N1 application*) Search modes - Boolean/Phrase Interface - EBSCOhost Research Databases
Search Screen - Advanced Search
Database - CINAHL Plus with Full Text Display
S192 TI (pathways N1 education*) OR AB (pathways N1 education*) Search modes - Boolean/Phrase Interface - EBSCOhost Research Databases
Search Screen - Advanced Search
Database - CINAHL Plus with Full Text Display
S191 TI "transition* year" OR AB "transition* year" Search modes - Boolean/Phrase Interface - EBSCOhost Research Databases
Search Screen - Advanced Search
Database - CINAHL Plus with Full Text Display
S190 TI "foundation year" OR AB "foundation year" Search modes - Boolean/Phrase Interface - EBSCOhost Research Databases
Search Screen - Advanced Search
Database - CINAHL Plus with Full Text Display
S189 TI "transition program#" OR AB "transition program#" Search modes - Boolean/Phrase Interface - EBSCOhost Research Databases
Search Screen - Advanced Search
Database - CINAHL Plus with Full Text Display
S188 TI scholarship# OR AB scholarship# Search modes - Boolean/Phrase Interface - EBSCOhost Research Databases
Search Screen - Advanced Search
Database - CINAHL Plus with Full Text Display
S187 TI (school N1 retention) OR AB (school N1 retention) Search modes - Boolean/Phrase Interface - EBSCOhost Research Databases
Search Screen - Advanced Search
Database - CINAHL Plus with Full Text Display
S186 TI "education disruption*" OR AB "education disruption*" Search modes - Boolean/Phrase Interface - EBSCOhost Research Databases
Search Screen - Advanced Search
Database - CINAHL Plus with Full Text Display
S185 TI dropout OR AB dropout Search modes - Boolean/Phrase Interface - EBSCOhost Research Databases
Search Screen - Advanced Search

Database - CINAHL Plus with Full Text Display
S184 TI diploma# OR AB diploma# Search modes - Boolean/Phrase Interface - EBSCOhost Research Databases
Search Screen - Advanced Search
Database - CINAHL Plus with Full Text Display
S183 TI "academic achievement" OR AB "academic achievement" Search modes - Boolean/Phrase Interface - EBSCOhost Research Databases
Search Screen - Advanced Search
Database - CINAHL Plus with Full Text Display
S182 TI (education* N1 inequality) OR AB (education* N1 inequality) Search modes - Boolean/Phrase Interface - EBSCOhost Research Databases
Search Screen - Advanced Search
Database - CINAHL Plus with Full Text Display
S181 TI (education* N1 difference#) OR AB (education* N1 difference#) Search modes - Boolean/Phrase Interface - EBSCOhost Research Databases
Search Screen - Advanced Search
Database - CINAHL Plus with Full Text Display
S180 TI (education* N1 difference#) OR AB (education* N1 difference#) Search modes - Boolean/Phrase Interface - EBSCOhost Research Databases
Search Screen - Advanced Search
Database - CINAHL Plus with Full Text Display
S179 TI (education* N1 level) OR AB (education* N1 level) Search modes - Boolean/Phrase Interface - EBSCOhost Research Databases
Search Screen - Advanced Search
Database - CINAHL Plus with Full Text Display
S178 TI (education* N1 attainment) OR AB (education* N1 attainment) Search modes - Boolean/Phrase Interface - EBSCOhost Research Databases
Search Screen - Advanced Search
Database - CINAHL Plus with Full Text Display
S177 TI (education* N1 status) OR AB (education* N1 status) Search modes - Boolean/Phrase Interface - EBSCOhost Research Databases
Search Screen - Advanced Search
Database - CINAHL Plus with Full Text Display
S176 TI "undergraduate education" OR AB "undergraduate education" Search modes - Boolean/Phrase Interface - EBSCOhost Research Databases
Search Screen - Advanced Search
Database - CINAHL Plus with Full Text Display
S175 TI "postgraduate education" OR AB "postgraduate education" Search modes - Boolean/Phrase Interface - EBSCOhost Research Databases
Search Screen - Advanced Search
Database - CINAHL Plus with Full Text Display
S174 TI "higher education" OR AB "higher education" Search modes - Boolean/Phrase Interface - EBSCOhost Research Databases
Search Screen - Advanced Search
Database - CINAHL Plus with Full Text Display
S173 TI (workplace N2 placement) OR AB (workplace N2 placement) Search modes - Boolean/Phrase Interface - EBSCOhost Research Databases
Search Screen - Advanced Search
Database - CINAHL Plus with Full Text Display
S172 TI (employment N2 placement) OR AB (employment N2 placement) Search modes - Boolean/Phrase Interface - EBSCOhost Research Databases

Search Screen - Advanced Search
Database - CINAHL Plus with Full Text Display
S171 TI (work N1 accommodat*) OR AB (work N1 accommodat*) Search modes - Boolean/Phrase Interface - EBSCOhost Research Databases
Search Screen - Advanced Search
Database - CINAHL Plus with Full Text Display
S170 TI "vocational readiness" OR AB "vocational readiness" Search modes - Boolean/Phrase Interface - EBSCOhost Research Databases
Search Screen - Advanced Search
Database - CINAHL Plus with Full Text Display
S169 TI "vocational guidance" OR AB "vocational guidance" Search modes - Boolean/Phrase Interface - EBSCOhost Research Databases
Search Screen - Advanced Search
Database - CINAHL Plus with Full Text Display
S168 TI (peer# N1 support*) OR AB (peer# N1 support*) Search modes - Boolean/Phrase Interface - EBSCOhost Research Databases
Search Screen - Advanced Search
Database - CINAHL Plus with Full Text Display
S167 (TI (occupation* N1 (ready or readiness))) OR (AB (occupation* N1 (ready or readiness))) Search modes - Boolean/Phrase Interface - EBSCOhost Research Databases
Search Screen - Advanced Search
Database - CINAHL Plus with Full Text Display
S166 TI mentoring OR AB mentoring Search modes - Boolean/Phrase Interface - EBSCOhost Research Databases
Search Screen - Advanced Search
Database - CINAHL Plus with Full Text Display
S165 TI (interview# N1 mock) OR AB (interview# N1 mock) Search modes - Boolean/Phrase Interface - EBSCOhost Research Databases
Search Screen - Advanced Search
Database - CINAHL Plus with Full Text Display
S164 TI (interview# N1 support*) OR AB (interview# N1 support*) Search modes - Boolean/Phrase Interface - EBSCOhost Research Databases
Search Screen - Advanced Search
Database - CINAHL Plus with Full Text Display
S163 TI "government plan#" OR AB "government plan#" Search modes - Boolean/Phrase Interface - EBSCOhost Research Databases
Search Screen - Advanced Search
Database - CINAHL Plus with Full Text Display
S162 TI (training N1 skill#) OR AB (training N1 skill#) Search modes - Boolean/Phrase Interface - EBSCOhost Research Databases
Search Screen - Advanced Search
Database - CINAHL Plus with Full Text Display
S161 S133 OR S134 OR S135 OR S136 OR S137 OR S138 OR S139 OR S140 OR S141 OR S142 OR S143 OR S144 OR S145 OR S146 OR S147 OR S148 OR S149 OR S150 OR S151 OR S152 OR S153 OR S154 OR S155 OR S156 OR S157 OR S158 OR S159 OR S160 Search modes - Boolean/Phrase Interface - EBSCOhost Research Databases
Search Screen - Advanced Search
Database - CINAHL Plus with Full Text Display
S160 TI (accessibility N2 funding) OR AB (accessibility N2 funding) Search modes - Boolean/Phrase Interface - EBSCOhost Research Databases
Search Screen - Advanced Search

Database - CINAHL Plus with Full Text Display
S159 TI "medical tax credit#" OR AB "medical tax credit#" Search modes - Boolean/Phrase Interface - EBSCOhost Research Databases
Search Screen - Advanced Search
Database - CINAHL Plus with Full Text Display
S158 TI "disability savings plan#" OR AB "disability savings plan#" Search modes - Boolean/Phrase Interface - EBSCOhost Research Databases
Search Screen - Advanced Search
Database - CINAHL Plus with Full Text Display
S157 TI (disability N2 "tax credit#") OR AB (disability N2 "tax credit#") Search modes - Boolean/Phrase Interface - EBSCOhost Research Databases
Search Screen - Advanced Search
Database - CINAHL Plus with Full Text Display
S156 TI "disability support" OR AB "disability support" Search modes - Boolean/Phrase Interface - EBSCOhost Research Databases
Search Screen - Advanced Search
Database - CINAHL Plus with Full Text Display
S155 TI (disability N2 grant*) OR AB (disability N2 grant*) Search modes - Boolean/Phrase Interface - EBSCOhost Research Databases
Search Screen - Advanced Search
Database - CINAHL Plus with Full Text Display
S154 TI welfare OR AB welfare Search modes - Boolean/Phrase Interface - EBSCOhost Research Databases
Search Screen - Advanced Search
Database - CINAHL Plus with Full Text Display
S153 TI (state N1 benefit#) OR AB (state N1 benefit#) Search modes - Boolean/Phrase Interface - EBSCOhost Research Databases
Search Screen - Advanced Search
Database - CINAHL Plus with Full Text Display
S152 TI "guaranteed minimum income" OR AB "guaranteed minimum income" Search modes - Boolean/Phrase Interface - EBSCOhost Research Databases
Search Screen - Advanced Search
Database - CINAHL Plus with Full Text Display
S151 TI "guaranteed income support" OR AB "guaranteed income support" Search modes - Boolean/Phrase Interface - EBSCOhost Research Databases
Search Screen - Advanced Search
Database - CINAHL Plus with Full Text Display
S150 TI flexicurity OR AB flexicurity Search modes - Boolean/Phrase Interface - EBSCOhost Research Databases
Search Screen - Advanced Search
Database - CINAHL Plus with Full Text Display
S149 TI workfare OR AB workfare Search modes - Boolean/Phrase Interface - EBSCOhost Research Databases
Search Screen - Advanced Search
Database - CINAHL Plus with Full Text Display
S148 TI "unemployment assistan*" OR AB "unemployment assistan*" Search modes - Boolean/Phrase Interface - EBSCOhost Research Databases
Search Screen - Advanced Search
Database - CINAHL Plus with Full Text Display
S147 TI UBI OR AB UBI Search modes - Boolean/Phrase Interface - EBSCOhost Research Databases

Search Screen - Advanced Search
Database - CINAHL Plus with Full Text Display
S146 TI "basic income" OR AB "basic income" Search modes - Boolean/Phrase Interface - EBSCOhost Research Databases
Search Screen - Advanced Search
Database - CINAHL Plus with Full Text Display
S145 TI (disab* N2 benefit#) OR AB (disab* N2 benefit#) Search modes - Boolean/Phrase Interface - EBSCOhost Research Databases
Search Screen - Advanced Search
Database - CINAHL Plus with Full Text Display
S144 TI "income security" OR AB "income security" Search modes - Boolean/Phrase Interface - EBSCOhost Research Databases
Search Screen - Advanced Search
Database - CINAHL Plus with Full Text Display
S143 TI "social protection" OR AB "social protection"Search modes - Boolean/Phrase Interface - EBSCOhost Research Databases
Search Screen - Advanced Search
Database - CINAHL Plus with Full Text Display
S142 TI (unemployment N2 benefit#) OR AB (unemployment N2 benefit#) Search modes - Boolean/Phrase Interface - EBSCOhost Research Databases
Search Screen - Advanced Search
Database - CINAHL Plus with Full Text Display
S141 (MH "Insurance, Health") Search modes - Boolean/Phrase Interface - EBSCOhost Research Databases
Search Screen - Advanced Search
Database - CINAHL Plus with Full Text Display
S140 TI "health insurance" OR AB "health insurance" Search modes - Boolean/Phrase Interface - EBSCOhost Research Databases
Search Screen - Advanced Search
Database - CINAHL Plus with Full Text Display
S139 (MH "Worker's Compensation")Search modes - Boolean/Phrase Interface - EBSCOhost Research Databases
Search Screen - Advanced Search
Database - CINAHL Plus with Full Text Display
S138 TI "worker* compensation" OR AB "worker* compensation" Search modes - Boolean/Phrase Interface - EBSCOhost Research Databases
Search Screen - Advanced Search
Database - CINAHL Plus with Full Text Display
S137 (MH "Economic and Social Security") Search modes - Boolean/Phrase Interface - EBSCOhost Research Databases
Search Screen - Advanced Search
Database - CINAHL Plus with Full Text Display
S136 TI "employment insurance" OR AB "employment insurance" Search modes - Boolean/Phrase Interface - EBSCOhost Research Databases
Search Screen - Advanced Search
Database - CINAHL Plus with Full Text Display
S135 TI (disability N2 benefit*) OR AB (disability N2 benefit*) Search modes - Boolean/Phrase Interface - EBSCOhost Research Databases
Search Screen - Advanced Search
Database - CINAHL Plus with Full Text Display

S134 TI (disability N2 support*) OR AB (disability N2 support*) Search modes - Boolean/Phrase Interface - EBSCOhost Research Databases
Search Screen - Advanced Search
Database - CINAHL Plus with Full Text Display
S133 TI (income N2 support*) OR AB (income N2 support*) Search modes - Boolean/Phrase Interface - EBSCOhost Research Databases
Search Screen - Advanced Search
Database - CINAHL Plus with Full Text Display
S132 S93 OR S94 OR S95 OR S96 OR S97 OR S98 OR S99 OR S100 OR S101 OR S102 OR S103 OR S104 OR S105 OR S106 OR S107 OR S108 OR S109 OR S110 OR S111 OR S112 OR S113 OR S114 OR S115 OR S116 OR S117 OR S118 OR S119 OR S120 OR S121 OR S122 OR S123 OR S124 OR S125 OR S126 OR S127 OR S128 OR S129 OR S130 OR S131 Search modes - Boolean/Phrase Interface - EBSCOhost Research Databases
Search Screen - Advanced Search
Database - CINAHL Plus with Full Text Display
S131 TI "small business*" OR AB "small business*" Search modes - Boolean/Phrase Interface - EBSCOhost Research Databases
Search Screen - Advanced Search
Database - CINAHL Plus with Full Text Display
S130 TI entrepreneurship OR AB entrepreneurship Search modes - Boolean/Phrase Interface - EBSCOhost Research Databases
Search Screen - Advanced Search
Database - CINAHL Plus with Full Text Display
S129 TI "seed money" OR AB "seed money" Search modes - Boolean/Phrase Interface - EBSCOhost Research Databases
Search Screen - Advanced Search
Database - CINAHL Plus with Full Text Display
S128 TI incubator# OR AB incubator# Search modes - Boolean/Phrase Interface - EBSCOhost Research Databases
Search Screen - Advanced Search
Database - CINAHL Plus with Full Text Display
S127 TI start-up# OR AB start-up# Search modes - Boolean/Phrase Interface - EBSCOhost Research Databases
Search Screen - Advanced Search
Database - CINAHL Plus with Full Text Display
S126 TI "social enterprise#" OR AB "social enterprise#" Search modes - Boolean/Phrase Interface - EBSCOhost Research Databases
Search Screen - Advanced Search
Database - CINAHL Plus with Full Text Display
S125 TI self-employment OR AB self-employment Search modes - Boolean/Phrase Interface - EBSCOhost Research Databases
Search Screen - Advanced Search
Database - CINAHL Plus with Full Text Display
S124 TI "job readiness" OR AB "job readiness" Search modes - Boolean/Phrase Interface - EBSCOhost Research Databases
Search Screen - Advanced Search
Database - CINAHL Plus with Full Text Display
S123 TI internship# OR AB internship# Search modes - Boolean/Phrase Interface - EBSCOhost Research Databases
Search Screen - Advanced Search
Database - CINAHL Plus with Full Text Display

S122 TI practicum# OR AB practicum# Search modes - Boolean/Phrase Interface - EBSCOhost Research Databases
Search Screen - Advanced Search
Database - CINAHL Plus with Full Text Display
S121 TI "job subsid*" OR AB "job subsid*" Search modes - Boolean/Phrase Interface - EBSCOhost Research Databases
Search Screen - Advanced Search
Database - CINAHL Plus with Full Text Display
S120 TI "employee intensive program#" OR AB "employee intensive program#" Search modes - Boolean/Phrase Interface - EBSCOhost Research Databases
Search Screen - Advanced Search
Database - CINAHL Plus with Full Text Display
S119 TI (career N2 advancement) OR AB (career N2 advancement) Search modes - Boolean/Phrase Interface - EBSCOhost Research Databases
Search Screen - Advanced Search
Database - CINAHL Plus with Full Text Display
S118 TI ("labo#r market" N2 advancement) OR AB ("labo#r market" N2 advancement) Search modes - Boolean/Phrase Interface - EBSCOhost Research Databases
Search Screen - Advanced Search
Database - CINAHL Plus with Full Text Display
S117 TI "labo#r market entry" OR AB "labo#r market entry" Search modes - Boolean/Phrase Interface - EBSCOhost Research Databases
Search Screen - Advanced Search
Database - CINAHL Plus with Full Text Display
S116 TI "job search*" OR AB "job search*" Search modes - Boolean/Phrase Interface - EBSCOhost Research Databases
Search Screen - Advanced Search
Database - CINAHL Plus with Full Text Display
S115 TI "job stability" OR AB "job stability" Search modes - Boolean/Phrase Interface - EBSCOhost Research Databases
Search Screen - Advanced Search
Database - CINAHL Plus with Full Text Display
S114 TI "labo#r market engagement" OR AB "labo#r market engagement" Search modes - Boolean/Phrase Interface - EBSCOhost Research Databases
Search Screen - Advanced Search
Database - CINAHL Plus with Full Text Display
S113 TI "labo#r market integration" OR AB "labo#r market integration" Search modes - Boolean/Phrase Interface - EBSCOhost Research Databases
Search Screen - Advanced Search
Database - CINAHL Plus with Full Text Display
S112 TI ("work disability" N2 prevention) OR AB ("work disability" N2 prevention) Search modes - Boolean/Phrase Interface - EBSCOhost Research Databases
Search Screen - Advanced Search
Database - CINAHL Plus with Full Text Display
S111 TI ("labo#r market" N2 participat*) OR AB ("labo#r market" N2 participat*) Search modes - Boolean/Phrase Interface - EBSCOhost Research Databases
Search Screen - Advanced Search
Database - CINAHL Plus with Full Text Display
S110 (TI ("labo#r market" N2 (policies or policy))) OR (AB ("labo#r market" N2 (policies or policy))) Search modes - Boolean/Phrase Interface - EBSCOhost Research Databases
Search Screen - Advanced Search

Database - CINAHL Plus with Full Text Display
S109 TI (work* N2 inclusion) OR AB (work* N2 inclusion) Search modes - Boolean/Phrase Interface - EBSCOhost Research Databases
Search Screen - Advanced Search
Database - CINAHL Plus with Full Text Display
S108 TI (work* N2 accessibility) OR AB (work* N2 accessibility) Search modes - Boolean/Phrase Interface - EBSCOhost Research Databases
Search Screen - Advanced Search
Database - CINAHL Plus with Full Text Display
S107 TI upskilling OR AB upskilling Search modes - Boolean/Phrase Interface - EBSCOhost Research Databases
Search Screen - Advanced Search
Database - CINAHL Plus with Full Text Display
S106 TI reskilling OR AB reskilling Search modes - Boolean/Phrase Interface - EBSCOhost Research Databases
Search Screen - Advanced Search
Database - CINAHL Plus with Full Text Display
S105 TI "job skilling" OR AB "job skilling" Search modes - Boolean/Phrase Interface - EBSCOhost Research Databases
Search Screen - Advanced Search
Database - CINAHL Plus with Full Text Display
S104 TI ("school to work" N2 transition) OR AB ("school to work" N2 transition) Search modes - Boolean/Phrase Interface - EBSCOhost Research Databases
Search Screen - Advanced Search
Database - CINAHL Plus with Full Text Display
S103 TI "vocational rehabilitation" OR AB "vocational rehabilitation" Search modes - Boolean/Phrase Interface - EBSCOhost Research Databases
Search Screen - Advanced Search
Database - CINAHL Plus with Full Text Display
S102 TI "employment program#" OR AB "employment program#" Search modes - Boolean/Phrase Interface - EBSCOhost Research Databases
Search Screen - Advanced Search
Database - CINAHL Plus with Full Text Display
S101 TI (employment N3 transition*) OR AB (employment N3 transition*) Search modes - Boolean/Phrase Interface - EBSCOhost Research Databases
Search Screen - Advanced Search
Database - CINAHL Plus with Full Text Display
S100 TI (employment N3 support*) OR AB (employment N3 support*) Search modes - Boolean/Phrase Interface - EBSCOhost Research Databases
Search Screen - Advanced Search
Database - CINAHL Plus with Full Text Display
S99 (TI (employment N3 (ready or readiness))) OR (AB (employment N3 (ready or readiness))) Search modes - Boolean/Phrase Interface - EBSCOhost Research Databases
Search Screen - Advanced Search
Database - CINAHL Plus with Full Text Display
S98 (TI (accommodate or accommodation#)) OR (AB (accommodate or accommodation#)) Search modes - Boolean/Phrase Interface - EBSCOhost Research Databases
Search Screen - Advanced Search
Database - CINAHL Plus with Full Text Display
S97 TI "duty to accommodat*" OR AB "duty to accommodat*" Search modes - Boolean/Phrase Interface - EBSCOhost Research Databases

Search Screen - Advanced Search<br>Database - CINAHL Plus with Full Text Display<br>S96 (TI ("co-operative education" or "cooperative education")) OR (AB ("co-operative education" or "cooperative education")) Search modes - Boolean/Phrase Interface - EBSCOhost Research Databases<br>Search Screen - Advanced Search<br>Database - CINAHL Plus with Full Text Display<br>S95 (TI (career N2 (counseling or counselling))) OR (AB (career N2 (counseling or counselling))) Search modes - Boolean/Phrase Interface - EBSCOhost Research Databases<br>Search Screen - Advanced Search<br>Database - CINAHL Plus with Full Text Display<br>S94 TI (career N2 advice) OR AB (career N2 advice)Search modes - Boolean/Phrase Interface - EBSCOhost Research Databases<br>Search Screen - Advanced Search<br>Database - CINAHL Plus with Full Text Display<br>S93 TI "work-integrated learning" OR AB "work-integrated learning"Search modes - Boolean/Phrase Interface - EBSCOhost Research Databases<br>Search Screen - Advanced Search<br>Database - CINAHL Plus with Full Text Display<br>S92 S17 AND S91 Search modes - Boolean/Phrase Interface - EBSCOhost Research Databases<br>Search Screen - Advanced Search
Database - CINAHL Plus with Full Text Display<br>S91 S18 OR S19 OR S20 OR S21 OR S22 OR S23 OR S24 OR S25 OR S26 OR S27 OR S28 OR S29 OR S30 OR S31 OR S32 OR S33 OR S34 OR S35 OR S36 OR S37 OR S38 OR S39 OR S40 OR S41 OR S42 OR S43 OR S44 OR S45 OR S46 OR S47 OR S48 OR S49 OR S50 OR S51 OR S52 OR S53 OR S54 OR S55 OR S56 OR S57 OR S58 OR S59 OR S60 OR S61 OR S62 OR S63 OR S64 OR S65 OR S66 OR S67 OR S68 OR S69 OR S70 OR S71 OR S72 OR S73 OR S74 OR S75 OR S76 OR S77 OR S78 OR S79 OR S80 OR S81 OR S82 OR S83 OR S84 OR S85 OR S86 OR S87 OR S88 OR S89 OR S90 Search modes - Boolean/Phrase Interface - EBSCOhost Research Databases<br>Search Screen - Advanced Search<br>Database - CINAHL Plus with Full Text Display<br>S90 (MH "Premenstrual Dysphoric Disorder") Search modes - Boolean/Phrase Interface - EBSCOhost Research Databases<br>Search Screen - Advanced Search<br>Database - CINAHL Plus with Full Text Display<br>S89 TI "premenstrual symptom#" OR AB "premenstrual symptom#" Search modes - Boolean/Phrase Interface - EBSCOhost Research Databases<br>Search Screen - Advanced Search<br>Database - CINAHL Plus with Full Text Display<br>S88 (MH "Autoimmune Diseases") Search modes - Boolean/Phrase Interface - EBSCOhost Research Databases<br>Search Screen - Advanced Search<br>Database - CINAHL Plus with Full Text Display<br>S87 (TI ((autoimmune N2 condition*) OR (autoimmune N2 disease*) OR (autoimmune N2 disorder*))) OR (AB ((autoimmune N2 condition*) OR (autoimmune N2 disease*) OR (autoimmune N2 disorder*)))Search modes - Boolean/Phrase Interface - EBSCOhost Research Databases<br>Search Screen - Advanced Search<br>Database - CINAHL Plus with Full Text Display<br>S86 TI morphea OR AB morphea Search modes - Boolean/Phrase Interface - EBSCOhost Research Databases

Search Screen - Advanced Search
Database - CINAHL Plus with Full Text Display
S85 (MH "Scleroderma, Systemic") Search modes - Boolean/Phrase Interface - EBSCOhost Research Databases
Search Screen - Advanced Search
Database - CINAHL Plus with Full Text Display
S84 TI scleroderma OR AB scleroderma Search modes - Boolean/Phrase Interface - EBSCOhost Research Databases
Search Screen - Advanced Search
Database - CINAHL Plus with Full Text Display
S83 (MH "Psoriasis") Search modes - Boolean/Phrase Interface - EBSCOhost Research Databases
Search Screen - Advanced Search
Database - CINAHL Plus with Full Text Display
S82 TI psoriasis OR AB psoriasis Search modes - Boolean/Phrase Interface - EBSCOhost Research Databases
Search Screen - Advanced Search
Database - CINAHL Plus with Full Text Display
S81 TI Spondyloarthropathy OR AB Spondyloarthropathy Search modes - Boolean/Phrase Interface - EBSCOhost Research Databases
Search Screen - Advanced Search
Database - CINAHL Plus with Full Text Display
S80 TI (post-Covid* N2 syndrome#) OR AB (post-Covid* N2 syndrome#) Search modes - Boolean/Phrase Interface - EBSCOhost Research Databases
Search Screen - Advanced Search
Database - CINAHL Plus with Full Text Display
S79 TI (post-Covid* N2 symptom#) OR AB (post-Covid* N2 symptom#) Search modes - Boolean/Phrase Interface - EBSCOhost Research Databases
Search Screen - Advanced Search
Database - CINAHL Plus with Full Text Display
S78 (MH "Stress Disorders, Post-Traumatic") Search modes - Boolean/Phrase Interface - EBSCOhost Research Databases
Search Screen - Advanced Search
Database - CINAHL Plus with Full Text Display
S77 (TI (PTSD OR PTSS)) OR (AB (PTSD OR PTSS)) Search modes - Boolean/Phrase Interface - EBSCOhost Research Databases
Search Screen - Advanced Search
Database - CINAHL Plus with Full Text Display
S76 TI "posttraumatic stress" OR AB "posttraumatic stress" Search modes - Boolean/Phrase Interface - EBSCOhost Research Databases
Search Screen - Advanced Search
Database - CINAHL Plus with Full Text Display
S75 TI "post-traumatic stress" OR AB "post-traumatic stress"Search modes - Boolean/Phrase Interface - EBSCOhost Research Databases
Search Screen - Advanced Search
Database - CINAHL Plus with Full Text Display
S74 (MH "Bipolar Disorder") Search modes - Boolean/Phrase Interface - EBSCOhost Research Databases
Search Screen - Advanced Search
Database - CINAHL Plus with Full Text Display

S73 TI bipolar OR AB bipolar Search modes - Boolean/Phrase Interface - EBSCOhost Research Databases
Search Screen - Advanced Search
Database - CINAHL Plus with Full Text Display
S72 MH Rheumatic Diseases Search modes - Boolean/Phrase Interface - EBSCOhost Research Databases
Search Screen - Advanced Search
Database - CINAHL Plus with Full Text Display
S71 TI "rheumatic disease#" OR AB "rheumatic disease#" Search modes - Boolean/Phrase Interface - EBSCOhost Research Databases
Search Screen - Advanced Search
Database - CINAHL Plus with Full Text Display
S70 TI "psychiatric disabilit*" OR AB "psychiatric disabilit*" Search modes - Boolean/Phrase Interface - EBSCOhost Research Databases
Search Screen - Advanced Search
Database - CINAHL Plus with Full Text Display
S69 TI "progressive condition#" OR AB "progressive condition#" Search modes - Boolean/Phrase Interface - EBSCOhost Research Databases
Search Screen - Advanced Search
Database - CINAHL Plus with Full Text Display
S68 (MH "Musculoskeletal Diseases") Search modes - Boolean/Phrase Interface - EBSCOhost Research Databases
Search Screen - Advanced Search
Database - CINAHL Plus with Full Text Display
S67 TI "musculoskeletal disorder#" OR AB "musculoskeletal disorder#" Search modes - Boolean/Phrase Interface - EBSCOhost Research Databases
Search Screen - Advanced Search
Database - CINAHL Plus with Full Text Display
S66 (MH "Multiple Sclerosis") Search modes - Boolean/Phrase Interface - EBSCOhost Research Databases
Search Screen - Advanced Search
Database - CINAHL Plus with Full Text Display
S65 TI "multiple sclerosis" OR AB "multiple sclerosis" Search modes - Boolean/Phrase Interface - EBSCOhost Research Databases
Search Screen - Advanced Search
Database - CINAHL Plus with Full Text Display
S64 (MH "Affective Disorders") Search modes - Boolean/Phrase Interface - EBSCOhost Research Databases
Search Screen - Advanced Search
Database - CINAHL Plus with Full Text Display
S63 TI "mood disorder#" OR AB "mood disorder#" Search modes - Boolean/Phrase Interface - EBSCOhost Research Databases
Search Screen - Advanced Search
Database - CINAHL Plus with Full Text Display
S62 (MH "Migraine") Search modes - Boolean/Phrase Interface - EBSCOhost Research Databases
Search Screen - Advanced Search
Database - CINAHL Plus with Full Text Display
S61 TI migraine# OR AB migraine# Search modes - Boolean/Phrase Interface - EBSCOhost Research Databases
Search Screen - Advanced Search

Database - CINAHL Plus with Full Text Display
S60 TI "mental illness" OR AB "mental illness" Search modes - Boolean/Phrase Interface - EBSCOhost Research Databases
Search Screen - Advanced Search
Database - CINAHL Plus with Full Text Display
S59 TI "mental health" OR AB "mental health" Search modes - Boolean/Phrase Interface - EBSCOhost Research Databases
Search Screen - Advanced Search
Database - CINAHL Plus with Full Text Display
S58 (MH "Mental Disorders") Search modes - Boolean/Phrase Interface - EBSCOhost Research Databases
Search Screen - Advanced Search
Database - CINAHL Plus with Full Text Display
S57 TI "mental disorder#" OR AB "mental disorder#" Search modes - Boolean/Phrase Interface - EBSCOhost Research Databases
Search Screen - Advanced Search
Database - CINAHL Plus with Full Text Display
S56 (MH "Lupus Erythematosus, Systemic") OR (MH "Lupus Nephritis") Search modes - Boolean/Phrase Interface - EBSCOhost Research Databases
Search Screen - Advanced Search
Database - CINAHL Plus with Full Text Display
S55 TI lupus OR AB lupus Search modes - Boolean/Phrase Interface - EBSCOhost Research Databases
Search Screen - Advanced Search
Database - CINAHL Plus with Full Text Display
S54 (MH "Human Immunodeficiency Virus") Search modes - Boolean/Phrase Interface - EBSCOhost Research Databases
Search Screen - Advanced Search
Database - CINAHL Plus with Full Text Display
S53 TI HIV OR AB HIV Search modes - Boolean/Phrase Interface - EBSCOhost Research Databases
Search Screen - Advanced Search
Database - CINAHL Plus with Full Text Display
S52 (MH "Fibromyalgia") Search modes - Boolean/Phrase Interface - EBSCOhost Research Databases
Search Screen - Advanced Search
Database - CINAHL Plus with Full Text Display
S51 TI fibromyalgia OR AB fibromyalgia Search modes - Boolean/Phrase Interface - EBSCOhost Research Databases
Search Screen - Advanced Search
Database - CINAHL Plus with Full Text Display
S50 TI disability OR AB disability Search modes - Boolean/Phrase Interface - EBSCOhost Research Databases
Search Screen - Advanced Search
Database - CINAHL Plus with Full Text Display
S49 (MH "Epilepsy") Search modes - Boolean/Phrase Interface - EBSCOhost Research Databases
Search Screen - Advanced Search
Database - CINAHL Plus with Full Text Display
S48 TI epilepsy OR AB epilepsy Search modes - Boolean/Phrase Interface - EBSCOhost Research Databases

Search Screen - Advanced Search
Database - CINAHL Plus with Full Text Display
S47 (MH "Diabetes Mellitus, Type 1") OR (MH "Diabetes Mellitus, Type 2") Search modes - Boolean/Phrase Interface - EBSCOhost Research Databases
Search Screen - Advanced Search
Database - CINAHL Plus with Full Text Display
S46 TI diabetes OR AB diabetes Search modes - Boolean/Phrase Interface - EBSCOhost Research Databases
Search Screen - Advanced Search
Database - CINAHL Plus with Full Text Display
S45 TI dysthymia OR AB dysthymia Search modes - Boolean/Phrase Interface - EBSCOhost Research Databases
Search Screen - Advanced Search
Database - CINAHL Plus with Full Text Display
S44 (MH "Depression") Search modes - Boolean/Phrase Interface - EBSCOhost Research Databases
Search Screen - Advanced Search
Database - CINAHL Plus with Full Text Display
S43 TI depression OR AB depression Search modes - Boolean/Phrase Interface - EBSCOhost Research Databases
Search Screen - Advanced Search
Database - CINAHL Plus with Full Text Display
S42 (MH "Crohn Disease") Search modes - Boolean/Phrase Interface - EBSCOhost Research Databases
Search Screen - Advanced Search
Database - CINAHL Plus with Full Text Display
S41 TI "Crohn's disease" OR AB "Crohn's disease" Search modes - Boolean/Phrase Interface - EBSCOhost Research Databases
Search Screen - Advanced Search
Database - CINAHL Plus with Full Text Display
S40 (MH "Colitis, Ulcerative") Search modes - Boolean/Phrase Interface - EBSCOhost Research Databases
Search Screen - Advanced Search
Database - CINAHL Plus with Full Text Display
S39 TI (colitis N2 ulcerative) OR AB (colitis N2 ulcerative) Search modes - Boolean/Phrase Interface - EBSCOhost Research Databases
Search Screen - Advanced Search
Database - CINAHL Plus with Full Text Display
S38 (MH "Anxiety Disorders") Search modes - Boolean/Phrase Interface - EBSCOhost Research Databases
Search Screen - Advanced Search
Database - CINAHL Plus with Full Text Display
S37 TI "anxiety disorder#" OR AB "anxiety disorder#" Search modes - Boolean/Phrase Interface - EBSCOhost Research Databases
Search Screen - Advanced Search
Database - CINAHL Plus with Full Text Display
S36 (MH "Fatigue Syndrome, Chronic") Search modes - Boolean/Phrase Interface - EBSCOhost Research Databases
Search Screen - Advanced Search
Database - CINAHL Plus with Full Text Display

S35 TI "chronic fatigue" OR AB "chronic fatigue" Search modes - Boolean/Phrase Interface - EBSCOhost Research Databases
Search Screen - Advanced Search
Database - CINAHL Plus with Full Text Display
S34 (MH "Chronic Disease") Search modes - Boolean/Phrase Interface - EBSCOhost Research Databases
Search Screen - Advanced Search
Database - CINAHL Plus with Full Text Display
S33 TI "chronic disease#" OR AB "chronic disease#" Search modes - Boolean/Phrase Interface - EBSCOhost Research Databases
Search Screen - Advanced Search
Database - CINAHL Plus with Full Text Display
S32 (MH "Chronic Pain") Search modes - Boolean/Phrase Interface - EBSCOhost Research Databases
Search Screen - Advanced Search
Database - CINAHL Plus with Full Text Display
S31 TI "chronic pain" OR AB "chronic pain" Search modes - Boolean/Phrase Interface - EBSCOhost Research Databases
Search Screen - Advanced Search
Database - CINAHL Plus with Full Text Display
S30 (MH "Arthritis") Search modes - Boolean/Phrase Interface - EBSCOhost Research Databases
Search Screen - Advanced Search
Database - CINAHL Plus with Full Text Display
S29 TI arthritis OR AB arthritis Search modes - Boolean/Phrase Interface - EBSCOhost Research Databases
Search Screen - Advanced Search
Database - CINAHL Plus with Full Text Display
S28 (MH "Neoplasms") Search modes - Boolean/Phrase Interface - EBSCOhost Research Databases
Search Screen - Advanced Search
Database - CINAHL Plus with Full Text Display
S27 TI cancer OR AB cancer Search modes - Boolean/Phrase Interface - EBSCOhost Research Databases
Search Screen - Advanced Search
Database - CINAHL Plus with Full Text Display
S26 (MH "Inflammatory Bowel Diseases+") Search modes - Boolean/Phrase Interface - EBSCOhost Research Databases
Search Screen - Advanced Search
Database - CINAHL Plus with Full Text Display
S25 TI "bowel disease#" OR AB "bowel disease#" Search modes - Boolean/Phrase Interface - EBSCOhost Research Databases
Search Screen - Advanced Search
Database - CINAHL Plus with Full Text Display
S24 (TI (invisible N2 (disease* OR disabilit* OR symptom* OR health OR condition*))) OR (AB (invisible N2 (disease* OR disabilit* OR symptom* OR health OR condition*))) Search modes - Boolean/Phrase Interface - EBSCOhost Research Databases
Search Screen - Advanced Search
Database - CINAHL Plus with Full Text Display

S23 (TI (hidden N2 (disease* OR disabilit* OR symptom* OR health OR condition*))) OR (AB (hidden N2 (disease* OR disabilit* OR symptom* OR health OR condition*))) Search modes - Boolean/Phrase Interface - EBSCOhost Research Databases
Search Screen - Advanced Search
Database - CINAHL Plus with Full Text Display
S22 (TI (recurrent N2 (disease* OR disabilit* OR symptom* OR health OR condition*))) OR (AB (recurrent N2 (disease* OR disabilit* OR symptom* OR health OR condition*))) Search modes - Boolean/Phrase Interface - EBSCOhost Research Databases
Search Screen - Advanced Search
Database - CINAHL Plus with Full Text Display
S21 (TI (dynamic N2 (disease* OR disabilit* OR symptom* OR health OR condition*))) OR (AB (dynamic N2 (disease* OR disabilit* OR symptom* OR health OR condition*))) Search modes - Boolean/Phrase Interface - EBSCOhost Research Databases
Search Screen - Advanced Search
Database - CINAHL Plus with Full Text Display
S20 (TI (unpredictable N2 (disease* OR disabilit* OR symptom* OR health OR condition*))) OR (AB (unpredictable N2 (disease* OR disabilit* OR symptom* OR health OR condition*))) Search modes - Boolean/Phrase Interface - EBSCOhost Research Databases
Search Screen - Advanced Search
Database - CINAHL Plus with Full Text Display
S19 (TI (fluctuating N2 (disease* OR disabilit* OR symptom* OR health OR condition*))) OR (AB (fluctuating N2 (disease* OR disabilit* OR symptom* OR health OR condition*))) Search modes - Boolean/Phrase Interface - EBSCOhost Research Databases
Search Screen - Advanced Search
Database - CINAHL Plus with Full Text Display
S18 (TI (episodic N2 (disease* OR disabilit* OR symptom* OR health OR condition*))) OR (AB (episodic N2 (disease* OR disabilit* OR symptom* OR health OR condition*))) Search modes - Boolean/Phrase Interface - EBSCOhost Research Databases
Search Screen - Advanced Search
Database - CINAHL Plus with Full Text Display
S17 S1 OR S2 OR S3 OR S4 OR S5 OR S6 OR S7 OR S8 OR S9 OR S10 OR S11 OR S12 OR S13 OR S14 OR S15 OR S16 Search modes - Boolean/Phrase Interface - EBSCOhost Research Databases
Search Screen - Advanced Search
Database - CINAHL Plus with Full Text Display
S16 TI youth OR AB youth Search modes - Boolean/Phrase Interface - EBSCOhost Research Databases
Search Screen - Advanced Search
Database - CINAHL Plus with Full Text Display
S15 TI ("young people" OR "young person#") OR AB ("young people" OR "young person#") Search modes - Boolean/Phrase Interface - EBSCOhost Research Databases
Search Screen - Advanced Search
Database - CINAHL Plus with Full Text Display
S14 TI "young adult#" OR AB "young adult# Search modes - Boolean/Phrase Interface - EBSCOhost Research Databases
Search Screen - Advanced Search
Database - CINAHL Plus with Full Text Display
S13 TI (universit* N1 age#) OR AB (universit* N1 age#) Search modes - Boolean/Phrase Interface - EBSCOhost Research Databases
Search Screen - Advanced Search
Database - CINAHL Plus with Full Text Display

S12 TI teenage* OR AB teenage* Search modes - Boolean/Phrase Interface - EBSCOhost Research Databases<br>Search Screen - Advanced Search<br>Database - CINAHL Plus with Full Text Display<br>S11 TI teen# OR AB teen# Search modes - Boolean/Phrase Interface - EBSCOhost Research Databases<br>Search Screen - Advanced Search<br>Database - CINAHL Plus with Full Text Display<br>S10 TI "new worker#" OR AB "new worker#" Search modes - Boolean/Phrase Interface - EBSCOhost Research Databases<br>Search Screen - Advanced Search<br>Database - CINAHL Plus with Full Text Display<br>S9 TI millennial# OR AB millennial# Search modes - Boolean/Phrase Interface - EBSCOhost Research Databases<br>Search Screen - Advanced Search<br>Database - CINAHL Plus with Full Text Display<br>S8 TI ("high school*" N1 age#) OR AB ("high school*" N1 age#) Search modes - Boolean/Phrase Interface - EBSCOhost Research Databases<br>Search Screen - Advanced Search<br>Database - CINAHL Plus with Full Text Display<br>S7 TI ("Gen Z" or "Generation Z") OR AB ("Gen Z" or "Generation Z") Search modes - Boolean/Phrase Interface - EBSCOhost Research Databases<br>Search Screen - Advanced Search<br>Database - CINAHL Plus with Full Text Display<br>S6 TI "generation Y" OR AB "generation Y" Search modes - Boolean/Phrase Interface - EBSCOhost Research Databases<br>Search Screen - Advanced Search<br>Database - CINAHL Plus with Full Text Display<br>S5 TI "emerging adult*" OR AB "emerging adult*" Search modes - Boolean/Phrase Interface - EBSCOhost Research Databases<br>Search Screen - Advanced Search<br>Database - CINAHL Plus with Full Text Display<br>S4 TI "early adult*" OR AB "early adult*" Search modes - Boolean/Phrase Interface - EBSCOhost Research Databases<br>Search Screen - Advanced Search<br>Database - CINAHL Plus with Full Text Display<br>S3 TI (college N1 age#) OR AB (college N1 age#) Search modes - Boolean/Phrase Interface - EBSCOhost Research Databases<br>Search Screen - Advanced Search<br>Database - CINAHL Plus with Full Text Display<br>S2 TI adolescent# OR AB adolescent# Search modes - Boolean/Phrase Interface - EBSCOhost Research Databases<br>Search Screen - Advanced Search<br>Database - CINAHL Plus with Full Text Display<br>S1 (MH "Young Adult") Search modes - Boolean/Phrase Interface - EBSCOhost Research Databases<br>Search Screen - Advanced Search<br>Database - CINAHL Plus with Full Text Display
Database 5: Econlit (N=39)

S232 S64 AND S182 AND S231 Expanders - Apply equivalent subjects
Search modes - Boolean/Phrase Interface - EBSCOhost Research Databases
Search Screen - Advanced Search
Database - EconLit Display
S231 S199 OR S230 Expanders - Apply equivalent subjects
Search modes - Boolean/Phrase Interface - EBSCOhost Research Databases
Search Screen - Advanced Search
Database - EconLit Display
S230 S200 OR S201 OR S202 OR S203 OR S204 OR S205 OR S206 OR S207 OR S208 OR S209 OR S210 OR S211 OR S212 OR S213 OR S214 OR S215 OR S216 OR S217 OR S218 OR S219 OR S220 OR S221 OR S222 OR S223 OR S224 OR S225 OR S226 OR S227 OR S228 OR S229
Expanders - Apply equivalent subjects
Search modes - Boolean/Phrase Interface - EBSCOhost Research Databases
Search Screen - Advanced Search
Database - EconLit Display
S229 TI (social N1 accessibility) AND AB (social N1 accessibility) Expanders - Apply equivalent subjects
Search modes - Boolean/Phrase Interface - EBSCOhost Research Databases
Search Screen - Advanced Search
Database - EconLit Display
S228 TI "social mobility" AND AB "social mobility" Expanders - Apply equivalent subjects
Search modes - Boolean/Phrase Interface - EBSCOhost Research Databases
Search Screen - Advanced Search
Database - EconLit Display
S227 TI poverty OR AB poverty Expanders - Apply equivalent subjects
Search modes - Boolean/Phrase Interface - EBSCOhost Research Databases
Search Screen - Advanced Search
Database - EconLit Display
S226 TI "low income" OR AB "low income" Expanders - Apply equivalent subjects
Search modes - Boolean/Phrase Interface - EBSCOhost Research Databases
Search Screen - Advanced Search
Database - EconLit Display
S225 TI "cultural currency" OR AB "cultural currency" Expanders - Apply equivalent subjects
Search modes - Boolean/Phrase Interface - EBSCOhost Research Databases
Search Screen - Advanced Search
Database - EconLit Display
S224 TI "cultural capital" OR AB "cultural capital" Expanders - Apply equivalent subjects
Search modes - Boolean/Phrase Interface - EBSCOhost Research Databases
Search Screen - Advanced Search
Database - EconLit Display
S223 TI "financial status" OR AB "financial status" Expanders - Apply equivalent subjects
Search modes - Boolean/Phrase Interface - EBSCOhost Research Databases
Search Screen - Advanced Search
Database - EconLit Display
S222 TI "social inclusion" OR AB "social inclusion" Expanders - Apply equivalent subjects
Search modes - Boolean/Phrase Interface - EBSCOhost Research Databases
Search Screen - Advanced Search
Database - EconLit Display
S221 TI (standard N2 living) OR AB (standard N2 living) Expanders - Apply equivalent subjects

Search modes - Boolean/Phrase Interface - EBSCOhost Research Databases
Search Screen - Advanced Search
Database - EconLit Display
S220 TI (socioeconomic N1 status) OR AB (socioeconomic N1 status) Expanders - Apply equivalent subjects
Search modes - Boolean/Phrase Interface - EBSCOhost Research Databases
Search Screen - Advanced Search
Database - EconLit Display
S219 TI (socioeconomic N1 position#) OR AB (socioeconomic N1 position#) Expanders - Apply equivalent subjects
Search modes - Boolean/Phrase Interface - EBSCOhost Research Databases
Search Screen - Advanced Search
Database - EconLit Display
S218 TI (socioeconomic N1 health N1 difference#) OR AB (socioeconomic N1 health N1 difference#) Expanders - Apply equivalent subjects
Search modes - Boolean/Phrase Interface - EBSCOhost Research Databases
Search Screen - Advanced Search
Database - EconLit Display
S217 TI (socioeconomic N2 gradient#) OR AB (socioeconomic N2 gradient#) Expanders - Apply equivalent subjects
Search modes - Boolean/Phrase Interface - EBSCOhost Research Databases
Search Screen - Advanced Search
Database - EconLit Display
S216 TI (socioeconomic N2 factor*) OR AB (socioeconomic N2 factor*) Expanders - Apply equivalent subjects
Search modes - Boolean/Phrase Interface - EBSCOhost Research Databases
Search Screen - Advanced Search
Database - EconLit Display
S215 TI (socioeconomic N2 circumstance*) OR AB (socioeconomic N2 circumstance*) Expanders - Apply equivalent subjects
Search modes - Boolean/Phrase Interface - EBSCOhost Research Databases
Search Screen - Advanced Search
Database - EconLit Display
S214 TI (socioeconomic N2 attribut*) OR AB (socioeconomic N2 attribut*) Expanders - Apply equivalent subjects
Search modes - Boolean/Phrase Interface - EBSCOhost Research Databases
Search Screen - Advanced Search
Database - EconLit Display
S213 TI (socio N1 economic N1 variable#) OR AB (socio N1 economic N1 variable#) Expanders - Apply equivalent subjects
Search modes - Boolean/Phrase Interface - EBSCOhost Research Databases
Search Screen - Advanced Search
Database - EconLit Display
S212 TI (socio N1 economic N1 status) OR AB (socio N1 economic N1 status) Expanders - Apply equivalent subjects
Search modes - Boolean/Phrase Interface - EBSCOhost Research Databases
Search Screen - Advanced Search
Database - EconLit Display
S211 TI (socio N1 economic N1 position) OR AB (socio N1 economic N1 position) Expanders - Apply equivalent subjects
Search modes - Boolean/Phrase Interface - EBSCOhost Research Databases

Search Screen - Advanced Search
Database - EconLit Display
S210 TI (social n2 difference*) OR AB (social n2 difference*) Expanders - Apply equivalent subjects
Search modes - Boolean/Phrase Interface - EBSCOhost Research Databases
Search Screen - Advanced Search
Database - EconLit Display
S209 TI (social n2 depriv*) OR AB (social n2 depriv*) Expanders - Apply equivalent subjects
Search modes - Boolean/Phrase Interface - EBSCOhost Research Databases
Search Screen - Advanced Search
Database - EconLit Display
S208 TI (social n2 condition#) OR AB (social n2 condition#) Expanders - Apply equivalent subjects
Search modes - Boolean/Phrase Interface - EBSCOhost Research Databases
Search Screen - Advanced Search
Database - EconLit Display
S207 TI (social n2 class) OR AB (social n2 class) Expanders - Apply equivalent subjects
Search modes - Boolean/Phrase Interface - EBSCOhost Research Databases
Search Screen - Advanced Search
Database - EconLit Display
S206 TI (social n2 class) OR AB (social n2 class) Expanders - Apply equivalent subjects
Search modes - Boolean/Phrase Interface - EBSCOhost Research Databases
Search Screen - Advanced Search
Database - EconLit Display
S205 TI (social n2 capital) OR AB (social n2 capital) Expanders - Apply equivalent subjects
Search modes - Boolean/Phrase Interface - EBSCOhost Research Databases
Search Screen - Advanced Search
Database - EconLit Display
S204 TI (social n2 marginali#ed) OR AB (social n2 marginali#ed) Expanders - Apply equivalent subjects
Search modes - Boolean/Phrase Interface - EBSCOhost Research Databases
Search Screen - Advanced Search
Database - EconLit Display
S203 TI (social n2 inequity) OR AB (social n2 inequity) Expanders - Apply equivalent subjects
Search modes - Boolean/Phrase Interface - EBSCOhost Research Databases
Search Screen - Advanced Search
Database - EconLit Display
S202 TI (social n2 equity) OR AB (social n2 equity) Expanders - Apply equivalent subjects
Search modes - Boolean/Phrase Interface - EBSCOhost Research Databases
Search Screen - Advanced Search
Database - EconLit Display
S201 TI (social n2 disadvantag*) OR AB (social n2 disadvantag*) Expanders - Apply equivalent subjects
Search modes - Boolean/Phrase Interface - EBSCOhost Research Databases
Search Screen - Advanced Search
Database - EconLit Display
S200 TI (social n2 depriv*) OR AB (social n2 depriv*) Expanders - Apply equivalent subjects
Search modes - Boolean/Phrase Interface - EBSCOhost Research Databases

Search Screen - Advanced Search
Database - EconLit Display
S199 S183 OR S184 OR S185 OR S186 OR S187 OR S188 OR S189 OR S190 OR S191 OR S192 OR S193 OR S194 OR S195 OR S196 OR S197 OR S198 Expanders - Apply equivalent subjects
Search modes - Boolean/Phrase Interface - EBSCOhost Research Databases
Search Screen - Advanced Search
Database - EconLit Display
S198 TI (disease N2 impact*) OR AB (disease N2 impact*) Expanders - Apply equivalent subjects
Search modes - Boolean/Phrase Interface - EBSCOhost Research Databases
Search Screen - Advanced Search
Database - EconLit Display
S197 TI (disease N2 progress*) OR AB (disease N2 progress*) Expanders - Apply equivalent subjects
Search modes - Boolean/Phrase Interface - EBSCOhost Research Databases
Search Screen - Advanced Search
Database - EconLit Display
S196 (TI (injury or injuries)) OR (AB (injury or injuries)) Expanders - Apply equivalent subjects
Search modes - Boolean/Phrase Interface - EBSCOhost Research Databases
Search Screen - Advanced Search
Database - EconLit Display
S195 TI "health status" OR AB "health status"Expanders - Apply equivalent subjects
Search modes - Boolean/Phrase Interface - EBSCOhost Research Databases
Search Screen - Advanced Search
Database - EconLit Display
S194 TI "disease activity" OR AB "disease activity" Expanders - Apply equivalent subjects
Search modes - Boolean/Phrase Interface - EBSCOhost Research Databases
Search Screen - Advanced Search
Database - EconLit Display
S193 TI (disability N2 severity) OR AB (disability N2 severity) Expanders - Apply equivalent subjects
Search modes - Boolean/Phrase Interface - EBSCOhost Research Databases
Search Screen - Advanced Search
Database - EconLit Display
S192 TI (symptom# N2 severity) OR AB (symptom# N2 severity) Expanders - Apply equivalent subjects
Search modes - Boolean/Phrase Interface - EBSCOhost Research Databases
Search Screen - Advanced Search
Database - EconLit Display
S191 TI impairment OR AB impairment Expanders - Apply equivalent subjects
Search modes - Boolean/Phrase Interface - EBSCOhost Research Databases
Search Screen - Advanced Search
Database - EconLit Display
S190 TI (activity N2 limitation#) OR AB (activity N2 limitation#) Expanders - Apply equivalent subjects
Search modes - Boolean/Phrase Interface - EBSCOhost Research Databases
Search Screen - Advanced Search
Database - EconLit Display
S189 TI "quality of life" OR AB "quality of life" Expanders - Apply equivalent subjects

Search modes - Boolean/Phrase Interface - EBSCOhost Research Databases
Search Screen - Advanced Search
Database - EconLit Display
S188 TI well-being OR AB well-being Expanders - Apply equivalent subjects
Search modes - Boolean/Phrase Interface - EBSCOhost Research Databases
Search Screen - Advanced Search
Database - EconLit Display
S187 TI (self-rated N2 health) OR AB (self-rated N2 health) Expanders - Apply equivalent subjects
Search modes - Boolean/Phrase Interface - EBSCOhost Research Databases
Search Screen - Advanced Search
Database - EconLit Display
S186 TI (self-reported N2 health) OR AB (self-reported N2 health) Expanders - Apply equivalent subjects
Search modes - Boolean/Phrase Interface - EBSCOhost Research Databases
Search Screen - Advanced Search
Database - EconLit Display
S185 TI (health N2 equit*) OR AB (health N2 equit*) Expanders - Apply equivalent subjects
Search modes - Boolean/Phrase Interface - EBSCOhost Research Databases
Search Screen - Advanced Search
Database - EconLit Display
S184 TI (health N2 disparit*) OR AB (health N2 disparit*) Expanders - Apply equivalent subjects
Search modes - Boolean/Phrase Interface - EBSCOhost Research Databases
Search Screen - Advanced Search
Database - EconLit Display
S183 TI (health N2 difference*) OR AB (health N2 difference*) Expanders - Apply equivalent subjects
Search modes - Boolean/Phrase Interface - EBSCOhost Research Databases
Search Screen - Advanced Search
Database - EconLit Display
S182 S108 OR S136 OR S181 Expanders - Apply equivalent subjects
Search modes - Boolean/Phrase Interface - EBSCOhost Research Databases
Search Screen - Advanced Search
Database - EconLit Display
S181 S137 OR S138 OR S139 OR S140 OR S141 OR S142 OR S143 OR S144 OR S145 OR S146 OR S147 OR S148 OR S149 OR S150 OR S151 OR S152 OR S153 OR S154 OR S155 OR S156 OR S157 OR S158 OR S159 OR S160 OR S161 OR S162 OR S163 OR S164 OR S165 OR S166 OR S167 OR S168 OR S169 OR S170 OR S171 OR S172 OR S173 OR S174 OR S175 OR S176 OR S177 OR S178 OR S179 OR S180 Expanders - Apply equivalent subjects
Search modes - Boolean/Phrase Interface - EBSCOhost Research Databases
Search Screen - Advanced Search
Database - EconLit Display
S180 TI pushout# OR AB pushout# Expanders - Apply equivalent subjects
Search modes - Boolean/Phrase Interface - EBSCOhost Research Databases
Search Screen - Advanced Search
Database - EconLit Display
S179 TI "pre-entry to practice" OR AB "pre-entry to practice" Expanders - Apply equivalent subjects
Search modes - Boolean/Phrase Interface - EBSCOhost Research Databases
Search Screen - Advanced Search

Database - EconLit Display
S178 TI hackathon# OR AB hackathon# Expanders - Apply equivalent subjects
Search modes - Boolean/Phrase Interface - EBSCOhost Research Databases
Search Screen - Advanced Search
Database - EconLit Display
S177 TI "pitch competition" OR AB "pitch competition" Expanders - Apply equivalent subjects
Search modes - Boolean/Phrase Interface - EBSCOhost Research Databases
Search Screen - Advanced Search
Database - EconLit Display
S176 TI "continuing education" OR AB "continuing education" Expanders - Apply equivalent subjects
Search modes - Boolean/Phrase Interface - EBSCOhost Research Databases
Search Screen - Advanced Search
Database - EconLit Display
S175 TI "adult learning" OR AB "adult learning" Expanders - Apply equivalent subjects
Search modes - Boolean/Phrase Interface - EBSCOhost Research Databases
Search Screen - Advanced Search
Database - EconLit Display
S174 TI "adult education" OR AB "adult education" Expanders - Apply equivalent subjects
Search modes - Boolean/Phrase Interface - EBSCOhost Research Databases
Search Screen - Advanced Search
Database - EconLit Display
S173 TI GED OR AB GED Expanders - Apply equivalent subjects
Search modes - Boolean/Phrase Interface - EBSCOhost Research Databases
Search Screen - Advanced Search
Database - EconLit Display
S172 TI "high school equivalency" OR AB "high school equivalency" Expanders - Apply equivalent subjects
Search modes - Boolean/Phrase Interface - EBSCOhost Research Databases
Search Screen - Advanced Search
Database - EconLit Display
S171 TI "mature student#" OR AB "mature student#" Expanders - Apply equivalent subjects
Search modes - Boolean/Phrase Interface - EBSCOhost Research Databases
Search Screen - Advanced Search
Database - EconLit Display
S170 TI (university N1 application*) OR AB (university N1 application*) Expanders - Apply equivalent subjects
Search modes - Boolean/Phrase Interface - EBSCOhost Research Databases
Search Screen - Advanced Search
Database - EconLit Display
S169 TI (pathways N1 education*) OR AB (pathways N1 education*) Expanders - Apply equivalent subjects
Search modes - Boolean/Phrase Interface - EBSCOhost Research Databases
Search Screen - Advanced Search
Database - EconLit Display
S168 TI "transition* year" OR AB "transition* year" Expanders - Apply equivalent subjects
Search modes - Boolean/Phrase Interface - EBSCOhost Research Databases
Search Screen - Advanced Search
Database - EconLit Display
S167 TI "foundation year" OR AB "foundation year" Expanders - Apply equivalent subjects

Search modes - Boolean/Phrase Interface - EBSCOhost Research Databases
Search Screen - Advanced Search
Database - EconLit Display
S166 TI "transition program#" OR AB "transition program#" Expanders - Apply equivalent subjects
Search modes - Boolean/Phrase Interface - EBSCOhost Research Databases
Search Screen - Advanced Search
Database - EconLit Display
S165 TI scholarship# OR AB scholarship# Expanders - Apply equivalent subjects
Search modes - Boolean/Phrase Interface - EBSCOhost Research Databases
Search Screen - Advanced Search
Database - EconLit Display
S164 TI (school N1 retention) OR AB (school N1 retention) Expanders - Apply equivalent subjects
Search modes - Boolean/Phrase Interface - EBSCOhost Research Databases
Search Screen - Advanced Search
Database - EconLit Display
S163 TI "education disruption*" OR AB "education disruption*" Expanders - Apply equivalent subjects
Search modes - Boolean/Phrase Interface - EBSCOhost Research Databases
Search Screen - Advanced Search
Database - EconLit Display
S162 TI dropout OR AB dropout Expanders - Apply equivalent subjects
Search modes - Boolean/Phrase Interface - EBSCOhost Research Databases
Search Screen - Advanced Search
Database - EconLit Display
S161 TI "school graduation" OR AB "school graduation" Expanders - Apply equivalent subjects
Search modes - Boolean/Phrase Interface - EBSCOhost Research Databases
Search Screen - Advanced Search
Database - EconLit Display
S160 TI diploma# OR AB diploma# Expanders - Apply equivalent subjects
Search modes - Boolean/Phrase Interface - EBSCOhost Research Databases
Search Screen - Advanced Search
Database - EconLit Display
S159 TI "academic achievement" OR AB "academic achievement" Expanders - Apply equivalent subjects
Search modes - Boolean/Phrase Interface - EBSCOhost Research Databases
Search Screen - Advanced Search
Database - EconLit Display
S158 TI (education* N1 inequality) OR AB (education* N1 inequality) Expanders - Apply equivalent subjects
Search modes - Boolean/Phrase Interface - EBSCOhost Research Databases
Search Screen - Advanced Search
Database - EconLit Display
S157 TI (education* N1 difference#) OR AB (education* N1 difference#) Expanders - Apply equivalent subjects
Search modes - Boolean/Phrase Interface - EBSCOhost Research Databases
Search Screen - Advanced Search
Database - EconLit Display

S156 TI (education* N1 level) OR AB (education* N1 level) Expanders - Apply equivalent subjects
Search modes - Boolean/Phrase Interface - EBSCOhost Research Databases
Search Screen - Advanced Search
Database - EconLit Display
S155 TI (education* N1 attainment) OR AB (education* N1 attainment) Expanders - Apply equivalent subjects
Search modes - Boolean/Phrase Interface - EBSCOhost Research Databases
Search Screen - Advanced Search
Database - EconLit Display
S154 TI (education* N1 status) OR AB (education* N1 status) Expanders - Apply equivalent subjects
Search modes - Boolean/Phrase Interface - EBSCOhost Research Databases
Search Screen - Advanced Search
Database - EconLit Display
S153 TI "undergraduate education" OR AB "undergraduate education" Expanders - Apply equivalent subjects
Search modes - Boolean/Phrase Interface - EBSCOhost Research Databases
Search Screen - Advanced Search
Database - EconLit Display
S152 TI "postgraduate education" OR AB "postgraduate education" Expanders - Apply equivalent subjects
Search modes - Boolean/Phrase Interface - EBSCOhost Research Databases
Search Screen - Advanced Search
Database - EconLit Display
S151 TI "graduate education" OR AB "graduate education" Expanders - Apply equivalent subjects
Search modes - Boolean/Phrase Interface - EBSCOhost Research Databases
Search Screen - Advanced Search
Database - EconLit Display
S150 TI "higher education" OR AB "higher education" Expanders - Apply equivalent subjects
Search modes - Boolean/Phrase Interface - EBSCOhost Research Databases
Search Screen - Advanced Search
Database - EconLit Display
S149 TI (workplace N2 placement) OR AB (workplace N2 placement) Expanders - Apply equivalent subjects
Search modes - Boolean/Phrase Interface - EBSCOhost Research Databases
Search Screen - Advanced Search
Database - EconLit Display
S148 TI (employment N2 placement) OR AB (employment N2 placement) Expanders - Apply equivalent subjects
Search modes - Boolean/Phrase Interface - EBSCOhost Research Databases
Search Screen - Advanced Search
Database - EconLit Display
S147 TI (work N1 accommodat*) OR AB (work N1 accommodat*) Expanders - Apply equivalent subjects
Search modes - Boolean/Phrase Interface - EBSCOhost Research Databases
Search Screen - Advanced Search
Database - EconLit Display

S146 TI "vocational readiness" OR AB "vocational readiness" Expanders - Apply equivalent subjects
Search modes - Boolean/Phrase Interface - EBSCOhost Research Databases
Search Screen - Advanced Search
Database - EconLit Display
S145 TI "vocational guidance" OR AB "vocational guidance" Expanders - Apply equivalent subjects
Search modes - Boolean/Phrase Interface - EBSCOhost Research Databases
Search Screen - Advanced Search
Database - EconLit Display
S144 TI (peer# N1 support*) OR AB (peer# N1 support*) Expanders - Apply equivalent subjects
Search modes - Boolean/Phrase Interface - EBSCOhost Research Databases
Search Screen - Advanced Search
Database - EconLit Display
S143 (TI (occupation* N1 (ready or readiness))) OR (AB (occupation* N1 (ready or readiness))) Expanders - Apply equivalent subjects
Search modes - Boolean/Phrase Interface - EBSCOhost Research Databases
Search Screen - Advanced Search
Database - EconLit Display
S142 TI mentoring OR AB mentoring Expanders - Apply equivalent subjects
Search modes - Boolean/Phrase Interface - EBSCOhost Research Databases
Search Screen - Advanced Search
Database - EconLit Display
S141 TI (interview# N1 mock) OR AB (interview# N1 mock) Expanders - Apply equivalent subjects
Search modes - Boolean/Phrase Interface - EBSCOhost Research Databases
Search Screen - Advanced Search
Database - EconLit Display
S140 TI (interview# N1 support*) OR AB (interview# N1 support*) Expanders - Apply equivalent subjects
Search modes - Boolean/Phrase Interface - EBSCOhost Research Databases
Search Screen - Advanced Search
Database - EconLit Display
S139 TI (government N1 subsid*) OR AB (government N1 subsid*) Expanders - Apply equivalent subjects
Search modes - Boolean/Phrase Interface - EBSCOhost Research Databases
Search Screen - Advanced Search
Database - EconLit Display
S138 TI "government plan#" OR AB "government plan#" Expanders - Apply equivalent subjects
Search modes - Boolean/Phrase Interface - EBSCOhost Research Databases
Search Screen - Advanced Search
Database - EconLit Display
S137 TI (training N1 skill#) OR AB (training N1 skill#) Expanders - Apply equivalent subjects
Search modes - Boolean/Phrase Interface - EBSCOhost Research Databases
Search Screen - Advanced Search
Database - EconLit Display
S136 S109 OR S110 OR S111 OR S112 OR S113 OR S114 OR S115 OR S116 OR S117 OR S118 OR S119 OR S120 OR S121 OR S122 OR S123 OR S124 OR S125 OR S126 OR S127 OR S128 OR

S129 OR S130 OR S131 OR S132 OR S133 OR S134 OR S135 Expanders - Apply equivalent subjects
Search modes - Boolean/Phrase Interface - EBSCOhost Research Databases
Search Screen - Advanced Search
Database - EconLit Display
S135 TI (accessibility N2 funding) OR AB (accessibility N2 funding) Expanders - Apply equivalent subjects
Search modes - Boolean/Phrase Interface - EBSCOhost Research Databases
Search Screen - Advanced Search
Database - EconLit Display
S134 TI "medical tax credit#" OR AB "medical tax credit#" Expanders - Apply equivalent subjects
Search modes - Boolean/Phrase Interface - EBSCOhost Research Databases
Search Screen - Advanced Search
Database - EconLit Display
S133 TI "disability savings plan#" OR AB "disability savings plan#" Expanders - Apply equivalent subjects
Search modes - Boolean/Phrase Interface - EBSCOhost Research Databases
Search Screen - Advanced Search
Database - EconLit Display
S132 TI (disability N2 "tax credit#") OR AB (disability N2 "tax credit#") Expanders - Apply equivalent subjects
Search modes - Boolean/Phrase Interface - EBSCOhost Research Databases
Search Screen - Advanced Search
Database - EconLit Display
S131 TI "disability support" OR AB "disability support" Expanders - Apply equivalent subjects
Search modes - Boolean/Phrase Interface - EBSCOhost Research Databases
Search Screen - Advanced Search
Database - EconLit Display
S130 TI (disability N2 grant*) OR AB (disability N2 grant*) Expanders - Apply equivalent subjects
Search modes - Boolean/Phrase Interface - EBSCOhost Research Databases
Search Screen - Advanced Search
Database - EconLit Display
S129 TI welfare OR AB welfare Expanders - Apply equivalent subjects
Search modes - Boolean/Phrase Interface - EBSCOhost Research Databases
Search Screen - Advanced Search
Database - EconLit Display
S128 TI (uncompensated N1 care) OR AB (uncompensated N1 care) Expanders - Apply equivalent subjects
Search modes - Boolean/Phrase Interface - EBSCOhost Research Databases
Search Screen - Advanced Search
Database - EconLit Display
S127 TI (state N1 benefit#) OR AB (state N1 benefit#) Expanders - Apply equivalent subjects
Search modes - Boolean/Phrase Interface - EBSCOhost Research Databases
Search Screen - Advanced Search
Database - EconLit Display
S126 TI "guaranteed minimum income" OR AB "guaranteed minimum income" Expanders - Apply equivalent subjects

Search modes - Boolean/Phrase Interface - EBSCOhost Research Databases
Search Screen - Advanced Search
Database - EconLit Display
S125 TI "guaranteed income support" OR AB "guaranteed income support" Expanders - Apply equivalent subjects
Search modes - Boolean/Phrase Interface - EBSCOhost Research Databases
Search Screen - Advanced Search
Database - EconLit Display
S124 TI flexicurity OR AB flexicurity Expanders - Apply equivalent subjects
Search modes - Boolean/Phrase Interface - EBSCOhost Research Databases
Search Screen - Advanced Search
Database - EconLit Display
S123 TI workfare OR AB workfare Expanders - Apply equivalent subjects
Search modes - Boolean/Phrase Interface - EBSCOhost Research Databases
Search Screen - Advanced Search
Database - EconLit Display
S122 TI "unemployment assistan*" OR AB "unemployment assistan*" Expanders - Apply equivalent subjects
Search modes - Boolean/Phrase Interface - EBSCOhost Research Databases
Search Screen - Advanced Search
Database - EconLit Display
S121 TI UBI OR AB UBI Expanders - Apply equivalent subjects
Search modes - Boolean/Phrase Interface - EBSCOhost Research Databases
Search Screen - Advanced Search
Database - EconLit Display
S120 TI "basic income" OR AB "basic income" Expanders - Apply equivalent subjects
Search modes - Boolean/Phrase Interface - EBSCOhost Research Databases
Search Screen - Advanced Search
Database - EconLit Display
S119 TI (disab* N2 benefit#) OR AB (disab* N2 benefit#) Expanders - Apply equivalent subjects
Search modes - Boolean/Phrase Interface - EBSCOhost Research Databases
Search Screen - Advanced Search
Database - EconLit Display
S118 TI "income security" OR AB "income security" Expanders - Apply equivalent subjects
Search modes - Boolean/Phrase Interface - EBSCOhost Research Databases
Search Screen - Advanced Search
Database - EconLit Display
S117 TI "social protection" OR AB "social protection" Expanders - Apply equivalent subjects
Search modes - Boolean/Phrase Interface - EBSCOhost Research Databases
Search Screen - Advanced Search
Database - EconLit Display
S116 TI (unemployment N2 benefit#) OR AB (unemployment N2 benefit#) Expanders - Apply equivalent subjects
Search modes - Boolean/Phrase Interface - EBSCOhost Research Databases
Search Screen - Advanced Search
Database - EconLit Display
S115 TI "health insurance" OR AB "health insurance" Expanders - Apply equivalent subjects
Search modes - Boolean/Phrase Interface - EBSCOhost Research Databases
Search Screen - Advanced Search
Database - EconLit Display

S114 TI "worker* compensation" OR AB "worker* compensation" Expanders - Apply equivalent subjects
Search modes - Boolean/Phrase Interface - EBSCOhost Research Databases
Search Screen - Advanced Search
Database - EconLit Display
S113 ((ZU "social security")) or ((ZU "workmen's compensation")) Expanders - Apply equivalent subjects
Search modes - Boolean/Phrase Interface - EBSCOhost Research Databases
Search Screen - Advanced Search
Database - EconLit Display
S112 TI "employment insurance" OR AB "employment insurance" Expanders - Apply equivalent subjects
Search modes - Boolean/Phrase Interface - EBSCOhost Research Databases
Search Screen - Advanced Search
Database - EconLit Display
S111 TI (disability N2 benefit*) OR AB (disability N2 benefit*) Expanders - Apply equivalent subjects
Search modes - Boolean/Phrase Interface - EBSCOhost Research Databases
Search Screen - Advanced Search
Database - EconLit Display
S110 TI (disability N2 support*) OR AB (disability N2 support*) Expanders - Apply equivalent subjects
Search modes - Boolean/Phrase Interface - EBSCOhost Research Databases
Search Screen - Advanced Search
Database - EconLit Display
S109 TI (income N2 support*) OR AB (income N2 support*) Expanders - Apply equivalent subjects
Search modes - Boolean/Phrase Interface - EBSCOhost Research Databases
Search Screen - Advanced Search
Database - EconLit Display
S108 S65 OR S66 OR S67 OR S68 OR S69 OR S70 OR S71 OR S72 OR S73 OR S74 OR S75 OR S76 OR S77 OR S78 OR S79 OR S80 OR S81 OR S82 OR S83 OR S84 OR S85 OR S86 OR S87 OR S88 OR S89 OR S90 OR S91 OR S92 OR S93 OR S94 OR S95 OR S96 OR S97 OR S98 OR S99 OR S100 OR S101 OR S102 OR S103 OR S104 OR S105 OR S106 OR S107 Expanders - Apply equivalent subjects
Search modes - Boolean/Phrase Interface - EBSCOhost Research Databases
Search Screen - Advanced Search
Database - EconLit Display
S107 TI "small business*" OR AB "small business*" Expanders - Apply equivalent subjects
Search modes - Boolean/Phrase Interface - EBSCOhost Research Databases
Search Screen - Advanced Search
Database - EconLit Display
S106 TI entrepreneurship OR AB entrepreneurship Expanders - Apply equivalent subjects
Search modes - Boolean/Phrase Interface - EBSCOhost Research Databases
Search Screen - Advanced Search
Database - EconLit Display
S105 TI "seed money" OR AB "seed money" Expanders - Apply equivalent subjects
Search modes - Boolean/Phrase Interface - EBSCOhost Research Databases
Search Screen - Advanced Search
Database - EconLit Display
S104 TI incubator# OR AB incubator# Expanders - Apply equivalent subjects

Search modes - Boolean/Phrase Interface - EBSCOhost Research Databases
Search Screen - Advanced Search
Database - EconLit Display
S103 TI start-up# OR AB start-up# Expanders - Apply equivalent subjects
Search modes - Boolean/Phrase Interface - EBSCOhost Research Databases
Search Screen - Advanced Search
Database - EconLit Display
S102 TI "social enterprise#" OR AB "social enterprise#" Expanders - Apply equivalent subjects
Search modes - Boolean/Phrase Interface - EBSCOhost Research Databases
Search Screen - Advanced Search
Database - EconLit Display
S101 TI self-employment OR AB self-employment Expanders - Apply equivalent subjects
Search modes - Boolean/Phrase Interface - EBSCOhost Research Databases
Search Screen - Advanced Search
Database - EconLit Display
S100 TI "job readiness" OR AB "job readiness" Expanders - Apply equivalent subjects
Search modes - Boolean/Phrase Interface - EBSCOhost Research Databases
Search Screen - Advanced Search
Database - EconLit Display
S99 TI apprenticeship# OR AB apprenticeship# Expanders - Apply equivalent subjects
Search modes - Boolean/Phrase Interface - EBSCOhost Research Databases
Search Screen - Advanced Search
Database - EconLit Display
S98 TI internship# OR AB internship# Expanders - Apply equivalent subjects
Search modes - Boolean/Phrase Interface - EBSCOhost Research Databases
Search Screen - Advanced Search
Database - EconLit Display
S97 TI practicum# OR AB practicum# Expanders - Apply equivalent subjects
Search modes - Boolean/Phrase Interface - EBSCOhost Research Databases
Search Screen - Advanced Search
Database - EconLit Display
S96 TI "job subsid*" OR AB "job subsid*" Expanders - Apply equivalent subjects
Search modes - Boolean/Phrase Interface - EBSCOhost Research Databases
Search Screen - Advanced Search
Database - EconLit Display
S95 TI "employee intensive program#" OR AB "employee intensive program#" Expanders - Apply equivalent subjects
Search modes - Boolean/Phrase Interface - EBSCOhost Research Databases
Search Screen - Advanced Search
Database - EconLit Display
S94 TI (career N2 advancement) OR AB (career N2 advancement) Expanders - Apply equivalent subjects
Search modes - Boolean/Phrase Interface - EBSCOhost Research Databases
Search Screen - Advanced Search
Database - EconLit Display
S93 TI ("labo#r market" N2 advancement) OR AB ("labo#r market" N2 advancement) Expanders - Apply equivalent subjects
Search modes - Boolean/Phrase Interface - EBSCOhost Research Databases
Search Screen - Advanced Search
Database - EconLit Display

S92 TI "labo#r market entry" OR AB "labo#r market entry" Expanders - Apply equivalent subjects
Search modes - Boolean/Phrase Interface - EBSCOhost Research Databases
Search Screen - Advanced Search
Database - EconLit Display
S91 TI "job search*" OR AB "job search*" Expanders - Apply equivalent subjects
Search modes - Boolean/Phrase Interface - EBSCOhost Research Databases
Search Screen - Advanced Search
Database - EconLit Display
S90 TI "job stability" OR AB "job stability" Expanders - Apply equivalent subjects
Search modes - Boolean/Phrase Interface - EBSCOhost Research Databases
Search Screen - Advanced Search
Database - EconLit Display
S89 TI "labo#r market engagement" OR AB "labo#r market engagement" Expanders - Apply equivalent subjects
Search modes - Boolean/Phrase Interface - EBSCOhost Research Databases
Search Screen - Advanced Search
Database - EconLit Display
S88 TI "labo#r market integration" OR AB "labo#r market integration" Expanders - Apply equivalent subjects
Search modes - Boolean/Phrase Interface - EBSCOhost Research Databases
Search Screen - Advanced Search
Database - EconLit Display
S87 TI ("work disability" N2 prevention) OR AB ("work disability" N2 prevention) Expanders - Apply equivalent subjects
Search modes - Boolean/Phrase Interface - EBSCOhost Research Databases
Search Screen - Advanced Search
Database - EconLit Display
S86 TI ("labo#r market" N2 participat*) OR AB ("labo#r market" N2 participat*) Expanders - Apply equivalent subjects
Search modes - Boolean/Phrase Interface - EBSCOhost Research Databases
Search Screen - Advanced Search
Database - EconLit Display
S85 (TI ("labo#r market" N2 (policies or policy))) OR (AB ("labo#r market" N2 (policies or policy))) Expanders - Apply equivalent subjects
Search modes - Boolean/Phrase Interface - EBSCOhost Research Databases
Search Screen - Advanced Search
Database - EconLit Display
S84 TI (employment N2 inclusion) OR AB (employment N2 inclusion) Expanders - Apply equivalent subjects
Search modes - Boolean/Phrase Interface - EBSCOhost Research Databases
Search Screen - Advanced Search
Database - EconLit Display
S83 TI (employment N2 accessibility) OR AB (employment N2 accessibility)Expanders - Apply equivalent subjects
Search modes - Boolean/Phrase Interface - EBSCOhost Research Databases
Search Screen - Advanced Search
Database - EconLit Display
S82 TI (work* N2 inclusion) OR AB (work* N2 inclusion) Expanders - Apply equivalent subjects
Search modes - Boolean/Phrase Interface - EBSCOhost Research Databases

Search Screen - Advanced Search
Database - EconLit Display
S81 TI (work* N2 accessibility) OR AB (work* N2 accessibility) Expanders - Apply equivalent subjects
Search modes - Boolean/Phrase Interface - EBSCOhost Research Databases
Search Screen - Advanced Search
Database - EconLit Display
S80 TI upskilling OR AB upskilling Expanders - Apply equivalent subjects
Search modes - Boolean/Phrase Interface - EBSCOhost Research Databases
Search Screen - Advanced Search
Database - EconLit Display
S79 TI reskilling OR AB reskilling Expanders - Apply equivalent subjects
Search modes - Boolean/Phrase Interface - EBSCOhost Research Databases
Search Screen - Advanced Search
Database - EconLit Display
S78 TI "job skilling" OR AB "job skilling" Expanders - Apply equivalent subjects
Search modes - Boolean/Phrase Interface - EBSCOhost Research Databases
Search Screen - Advanced Search
Database - EconLit Display
S77 TI ("school to work" N2 transition) OR AB ("school to work" N2 transition) Expanders - Apply equivalent subjects
Search modes - Boolean/Phrase Interface - EBSCOhost Research Databases
Search Screen - Advanced Search
Database - EconLit Display
S76 TI "vocational rehabilitation" OR AB "vocational rehabilitation" Expanders - Apply equivalent subjects
Search modes - Boolean/Phrase Interface - EBSCOhost Research Databases
Search Screen - Advanced Search
Database - EconLit Display
S75 TI "employment program#" OR AB "employment program#" Expanders - Apply equivalent subjects
Search modes - Boolean/Phrase Interface - EBSCOhost Research Databases
Search Screen - Advanced Search
Database - EconLit Display
S74 TI "employment polic*" OR AB "employment polic*" Expanders - Apply equivalent subjects
Search modes - Boolean/Phrase Interface - EBSCOhost Research Databases
Search Screen - Advanced Search
Database - EconLit Display
S73 TI (employment N3 transition*) OR AB (employment N3 transition*) Expanders - Apply equivalent subjects
Search modes - Boolean/Phrase Interface - EBSCOhost Research Databases
Search Screen - Advanced Search
Database - EconLit Display
S72 TI (employment N3 support*) OR AB (employment N3 support*) Expanders - Apply equivalent subjects
Search modes - Boolean/Phrase Interface - EBSCOhost Research Databases
Search Screen - Advanced Search
Database - EconLit Display
S71 (TI (employment N3 (ready or readiness))) OR (AB (employment N3 (ready or readiness))) Expanders - Apply equivalent subjects

Search modes - Boolean/Phrase Interface - EBSCOhost Research Databases
Search Screen - Advanced Search
Database - EconLit Display
S70 (TI (accommodate or accommodation#)) OR (AB (accommodate or accommodation#)) Expanders - Apply equivalent subjects
Search modes - Boolean/Phrase Interface - EBSCOhost Research Databases
Search Screen - Advanced Search
Database - EconLit Display
S69 TI "duty to accommodat*" OR AB "duty to accommodat*" Expanders - Apply equivalent subjects
Search modes - Boolean/Phrase Interface - EBSCOhost Research Databases
Search Screen - Advanced Search
Database - EconLit Display
S68 (TI ("co-operative education" or "cooperative education")) OR (AB ("co-operative education" or "cooperative education")) Expanders - Apply equivalent subjects
Search modes - Boolean/Phrase Interface - EBSCOhost Research Databases
Search Screen - Advanced Search
Database - EconLit Display
S67 (TI (career N2 (counseling or counselling))) OR (AB (career N2 (counseling or counselling))) Expanders - Apply equivalent subjects
Search modes - Boolean/Phrase Interface - EBSCOhost Research Databases
Search Screen - Advanced Search
Database - EconLit Display
S66 TI (career N2 advice) OR AB (career N2 advice)Expanders - Apply equivalent subjects
Search modes - Boolean/Phrase Interface - EBSCOhost Research Databases
Search Screen - Advanced Search
Database - EconLit Display
S65 TI "work-integrated learning" OR AB "work-integrated learning"Expanders - Apply equivalent subjects
Search modes - Boolean/Phrase Interface - EBSCOhost Research Databases
Search Screen - Advanced Search
Database - EconLit Display
S64 S16 AND S63 Expanders - Apply equivalent subjects
Search modes - Boolean/Phrase Interface - EBSCOhost Research Databases
Search Screen - Advanced Search
Database - EconLit Display
S63 S17 OR S18 OR S19 OR S20 OR S21 OR S22 OR S23 OR S24 OR S25 OR S26 OR S27 OR S28 OR S29 OR S30 OR S31 OR S32 OR S33 OR S34 OR S35 OR S36 OR S37 OR S38 OR S39 OR S40 OR S41 OR S42 OR S43 OR S44 OR S45 OR S46 OR S47 OR S48 OR S49 OR S50 OR S51 OR S52 OR S53 OR S54 OR S55 OR S56 OR S57 OR S58 OR S59 OR S60 OR S61 OR S62 Expanders - Apply equivalent subjects
Search modes - Boolean/Phrase Interface - EBSCOhost Research Databases
Search Screen - Advanced Search
Database - EconLit Display
S62 TI "premenstrual symptom#" OR AB "premenstrual symptom#" Expanders - Apply equivalent subjects
Search modes - Boolean/Phrase Interface - EBSCOhost Research Databases
Search Screen - Advanced Search
Database - EconLit Display

S61 (TI ((autoimmune N2 condition*) OR (autoimmune N2 disease*) OR (autoimmune N2 disorder*))) OR (AB ((autoimmune N2 condition*) OR (autoimmune N2 disease*) OR (autoimmune N2 disorder*)))Expanders - Apply equivalent subjects
Search modes - Boolean/Phrase Interface - EBSCOhost Research Databases
Search Screen - Advanced Search
Database - EconLit Display
S60 TI morphea OR AB morphea Expanders - Apply equivalent subjects
Search modes - Boolean/Phrase Interface - EBSCOhost Research Databases
Search Screen - Advanced Search
Database - EconLit Display
S59 TI scleroderma OR AB scleroderma Expanders - Apply equivalent subjects
Search modes - Boolean/Phrase Interface - EBSCOhost Research Databases
Search Screen - Advanced Search
Database - EconLit Display
S58 TI psoriasis OR AB psoriasis Expanders - Apply equivalent subjects
Search modes - Boolean/Phrase Interface - EBSCOhost Research Databases
Search Screen - Advanced Search
Database - EconLit Display
S57 TI Spondyloarthropathy OR AB Spondyloarthropathy Expanders - Apply equivalent subjects
Search modes - Boolean/Phrase Interface - EBSCOhost Research Databases
Search Screen - Advanced Search
Database - EconLit Display
S56 TI (post-Covid* N2 syndrome#) OR AB (post-Covid* N2 syndrome#) Expanders - Apply equivalent subjects
Search modes - Boolean/Phrase Interface - EBSCOhost Research Databases
Search Screen - Advanced Search
Database - EconLit Display
S55 TI (post-Covid* N2 symptom#) OR AB (post-Covid* N2 symptom#) Expanders - Apply equivalent subjects
Search modes - Boolean/Phrase Interface - EBSCOhost Research Databases
Search Screen - Advanced Search
Database - EconLit Display
S54 (TI (PTSD OR PTSS)) OR (AB (PTSD OR PTSS)) Expanders - Apply equivalent subjects
Search modes - Boolean/Phrase Interface - EBSCOhost Research Databases
Search Screen - Advanced Search
Database - EconLit Display
S53 TI "posttraumatic stress" OR AB "posttraumatic stress" Expanders - Apply equivalent subjects
Search modes - Boolean/Phrase Interface - EBSCOhost Research Databases
Search Screen - Advanced Search
Database - EconLit Display
S52 TI "post-traumatic stress" OR AB "post-traumatic stress"Expanders - Apply equivalent subjects
Search modes - Boolean/Phrase Interface - EBSCOhost Research Databases
Search Screen - Advanced Search
Database - EconLit Display
S51 TI bipolar OR AB bipolar Expanders - Apply equivalent subjects
Search modes - Boolean/Phrase Interface - EBSCOhost Research Databases
Search Screen - Advanced Search

Database - EconLit Display
S50 TI "rheumatic disease#" OR AB "rheumatic disease#" Expanders - Apply equivalent subjects
Search modes - Boolean/Phrase Interface - EBSCOhost Research Databases
Search Screen - Advanced Search
Database - EconLit Display
S49 TI "psychiatric disabilit*" OR AB "psychiatric disabilit*" Expanders - Apply equivalent subjects
Search modes - Boolean/Phrase Interface - EBSCOhost Research Databases
Search Screen - Advanced Search
Database - EconLit Display
S48 TI "progressive condition#" OR AB "progressive condition#" Expanders - Apply equivalent subjects
Search modes - Boolean/Phrase Interface - EBSCOhost Research Databases
Search Screen - Advanced Search
Database - EconLit Display
S47 TI "musculoskeletal disorder#" OR AB "musculoskeletal disorder#" Expanders - Apply equivalent subjects
Search modes - Boolean/Phrase Interface - EBSCOhost Research Databases
Search Screen - Advanced Search
Database - EconLit Display
S46 TI "multiple sclerosis" OR AB "multiple sclerosis" Expanders - Apply equivalent subjects
Search modes - Boolean/Phrase Interface - EBSCOhost Research Databases
Search Screen - Advanced Search
Database - EconLit Display
S45 TI "mood disorder#" OR AB "mood disorder#" Expanders - Apply equivalent subjects
Search modes - Boolean/Phrase Interface - EBSCOhost Research Databases
Search Screen - Advanced Search
Database - EconLit Display
S44 TI migraine# OR AB migraine# Expanders - Apply equivalent subjects
Search modes - Boolean/Phrase Interface - EBSCOhost Research Databases
Search Screen - Advanced Search
Database - EconLit Display
S43 TI "mental illness" OR AB "mental illness" Expanders - Apply equivalent subjects
Search modes - Boolean/Phrase Interface - EBSCOhost Research Databases
Search Screen - Advanced Search
Database - EconLit Display
S42 TI "mental health" OR AB "mental health" Expanders - Apply equivalent subjects
Search modes - Boolean/Phrase Interface - EBSCOhost Research Databases
Search Screen - Advanced Search
Database - EconLit Display
S41 TI "mental disorder#" OR AB "mental disorder#" Expanders - Apply equivalent subjects
Search modes - Boolean/Phrase Interface - EBSCOhost Research Databases
Search Screen - Advanced Search
Database - EconLit Display
S40 TI lupus OR AB lupus Expanders - Apply equivalent subjects
Search modes - Boolean/Phrase Interface - EBSCOhost Research Databases
Search Screen - Advanced Search
Database - EconLit Display

S39 TI HIV OR AB HIV Expanders - Apply equivalent subjects
Search modes - Boolean/Phrase Interface - EBSCOhost Research Databases
Search Screen - Advanced Search
Database - EconLit Display
S38 TI fibromyalgia OR AB fibromyalgia Expanders - Apply equivalent subjects
Search modes - Boolean/Phrase Interface - EBSCOhost Research Databases
Search Screen - Advanced Search
Database - EconLit Display
S37 TI disability OR AB disability Expanders - Apply equivalent subjects
Search modes - Boolean/Phrase Interface - EBSCOhost Research Databases
Search Screen - Advanced Search
Database - EconLit Display
S36 TI epilepsy OR AB epilepsy Expanders - Apply equivalent subjects
Search modes - Boolean/Phrase Interface - EBSCOhost Research Databases
Search Screen - Advanced Search
Database - EconLit Display
S35 TI diabetes OR AB diabetes Expanders - Apply equivalent subjects
Search modes - Boolean/Phrase Interface - EBSCOhost Research Databases
Search Screen - Advanced Search
Database - EconLit Display
S34 TI dysthymia OR AB dysthymiaExpanders - Apply equivalent subjects
Search modes - Boolean/Phrase Interface - EBSCOhost Research Databases
Search Screen - Advanced Search
Database - EconLit Display
S33 TI depression OR AB depression Expanders - Apply equivalent subjects
Search modes - Boolean/Phrase Interface - EBSCOhost Research Databases
Search Screen - Advanced Search
Database - EconLit Display
S32 TI "Crohn's disease" OR AB "Crohn's disease" Expanders - Apply equivalent subjects
Search modes - Boolean/Phrase Interface - EBSCOhost Research Databases
Search Screen - Advanced Search
Database - EconLit Display
S31 TI (colitis N2 ulcerative) OR AB (colitis N2 ulcerative) Expanders - Apply equivalent subjects
Search modes - Boolean/Phrase Interface - EBSCOhost Research Databases
Search Screen - Advanced Search
Database - EconLit Display
S30 TI "anxiety disorder#" OR AB "anxiety disorder#" Expanders - Apply equivalent subjects
Search modes - Boolean/Phrase Interface - EBSCOhost Research Databases
Search Screen - Advanced Search
Database - EconLit Display
S29 TI "chronic fatigue" OR AB "chronic fatigue" Expanders - Apply equivalent subjects
Search modes - Boolean/Phrase Interface - EBSCOhost Research Databases
Search Screen - Advanced Search
Database - EconLit Display
S28 TI "chronic disease#" OR AB "chronic disease#"Expanders - Apply equivalent subjects
Search modes - Boolean/Phrase Interface - EBSCOhost Research Databases
Search Screen - Advanced Search
Database - EconLit Display
S27 TI "chronic pain" OR AB "chronic pain"Expanders - Apply equivalent subjects

Search modes - Boolean/Phrase Interface - EBSCOhost Research Databases
Search Screen - Advanced Search
Database - EconLit Display
S26 TI arthritis OR AB arthritis Expanders - Apply equivalent subjects
Search modes - Boolean/Phrase Interface - EBSCOhost Research Databases
Search Screen - Advanced Search
Database - EconLit Display
S25 TI cancer OR AB cancer Expanders - Apply equivalent subjects
Search modes - Boolean/Phrase Interface - EBSCOhost Research Databases
Search Screen - Advanced Search
Database - EconLit Display
S24 TI "bowel disease#" OR AB "bowel disease#" Expanders - Apply equivalent subjects
Search modes - Boolean/Phrase Interface - EBSCOhost Research Databases
Search Screen - Advanced Search
Database - EconLit Display
S23 (TI (invisible N2 (disease* OR disabilit* OR symptom* OR health OR condition*))) OR (AB (invisible N2 (disease* OR disabilit* OR symptom* OR health OR condition*))) Expanders - Apply equivalent subjects
Search modes - Boolean/Phrase Interface - EBSCOhost Research Databases
Search Screen - Advanced Search
Database - EconLit Display
S22 (TI (hidden N2 (disease* OR disabilit* OR symptom* OR health OR condition*))) OR (AB (hidden N2 (disease* OR disabilit* OR symptom* OR health OR condition*))) Expanders - Apply equivalent subjects
Search modes - Boolean/Phrase Interface - EBSCOhost Research Databases
Search Screen - Advanced Search
Database - EconLit Display
S21 (TI (recurrent N2 (disease* OR disabilit* OR symptom* OR health OR condition*))) OR (AB (recurrent N2 (disease* OR disabilit* OR symptom* OR health OR condition*))) Expanders - Apply equivalent subjects
Search modes - Boolean/Phrase Interface - EBSCOhost Research Databases
Search Screen - Advanced Search
Database - EconLit Display
S20 (TI (dynamic N2 (disease* OR disabilit* OR symptom* OR health OR condition*))) OR (AB (dynamic N2 (disease* OR disabilit* OR symptom* OR health OR condition*))) Expanders - Apply equivalent subjects
Search modes - Boolean/Phrase Interface - EBSCOhost Research Databases
Search Screen - Advanced Search
Database - EconLit Display
S19 (TI (unpredictable N2 (disease* OR disabilit* OR symptom* OR health OR condition*))) OR (AB (unpredictable N2 (disease* OR disabilit* OR symptom* OR health OR condition*))) Expanders - Apply equivalent subjects
Search modes - Boolean/Phrase Interface - EBSCOhost Research Databases
Search Screen - Advanced Search
Database - EconLit Display
S18 (TI (fluctuating N2 (disease* OR disabilit* OR symptom* OR health OR condition*))) OR (AB (fluctuating N2 (disease* OR disabilit* OR symptom* OR health OR condition*))) Expanders - Apply equivalent subjects
Search modes - Boolean/Phrase Interface - EBSCOhost Research Databases
Search Screen - Advanced Search
Database - EconLit Display

S17 (TI (episodic N2 (disease* OR disabilit* OR symptom* OR health OR condition*))) OR (AB (episodic N2 (disease* OR disabilit* OR symptom* OR health OR condition*))) Expanders - Apply equivalent subjects
Search modes - Boolean/Phrase Interface - EBSCOhost Research Databases
Search Screen - Advanced Search
Database - EconLit Display
S16 S1 OR S2 OR S3 OR S4 OR S5 OR S6 OR S7 OR S8 OR S9 OR S10 OR S11 OR S12 OR S13 OR S14 OR S15 Expanders - Apply equivalent subjects
Search modes - Boolean/Phrase Interface - EBSCOhost Research Databases
Search Screen - Advanced Search
Database - EconLit Display
S15 TI youth OR AB youth Expanders - Apply equivalent subjects
Search modes - Boolean/Phrase Interface - EBSCOhost Research Databases
Search Screen - Advanced Search
Database - EconLit Display
S14 TI ("young people" OR "young person#") OR AB ("young people" OR "young person#") Expanders - Apply equivalent subjects
Search modes - Boolean/Phrase Interface - EBSCOhost Research Databases
Search Screen - Advanced Search
Database - EconLit Display
S13 TI "young adult#" OR AB "young adult# Expanders - Apply equivalent subjects
Search modes - Boolean/Phrase Interface - EBSCOhost Research Databases
Search Screen - Advanced Search
Database - EconLit Display
S12 TI (universit* N1 age#) OR AB (universit* N1 age#) Expanders - Apply equivalent subjects
Search modes - Boolean/Phrase Interface - EBSCOhost Research Databases
Search Screen - Advanced Search
Database - EconLit Display
S11 TI teenage* OR AB teenage* Expanders - Apply equivalent subjects
Search modes - Boolean/Phrase Interface - EBSCOhost Research Databases
Search Screen - Advanced Search
Database - EconLit Display
S10 TI teen# OR AB teen# Expanders - Apply equivalent subjects
Search modes - Boolean/Phrase Interface - EBSCOhost Research Databases
Search Screen - Advanced Search
Database - EconLit Display
S9 TI "new worker#" OR AB "new worker#" Expanders - Apply equivalent subjects
Search modes - Boolean/Phrase Interface - EBSCOhost Research Databases
Search Screen - Advanced Search
Database - EconLit Display
S8 TI millennial# OR AB millennial# Expanders - Apply equivalent subjects
Search modes - Boolean/Phrase Interface - EBSCOhost Research Databases
Search Screen - Advanced Search
Database - EconLit Display
S7 TI ("high school*" N1 age*) OR AB ("high school*" N1 age*) Expanders - Apply equivalent subjects
Search modes - Boolean/Phrase Interface - EBSCOhost Research Databases
Search Screen - Advanced Search
Database - EconLit Display

S6 TI ("Gen Z" or "Generation Z") OR AB ("Gen Z" or "Generation Z") Expanders - Apply equivalent subjects
Search modes - Boolean/Phrase Interface - EBSCOhost Research Databases
Search Screen - Advanced Search
Database - EconLit Display
S5 TI "generation Y" OR AB "generation Y" Expanders - Apply equivalent subjects
Search modes - Boolean/Phrase Interface - EBSCOhost Research Databases
Search Screen - Advanced Search
Database - EconLit Display
S4 TI "emerging adult*" OR AB "emerging adult*" Expanders - Apply equivalent subjects
Search modes - Boolean/Phrase Interface - EBSCOhost Research Databases
Search Screen - Advanced Search
Database - EconLit Display
S3 TI "early adult*" OR AB "early adult*" Expanders - Apply equivalent subjects
Search modes - Boolean/Phrase Interface - EBSCOhost Research Databases
Search Screen - Advanced Search
Database - EconLit Display
S2 TI (college N1 age*) OR AB (college N1 age*) Expanders - Apply equivalent subjects
Search modes - Boolean/Phrase Interface - EBSCOhost Research Databases
Search Screen - Advanced Search
Database - EconLit Display
S1 TI adolescent# OR AB adolescent# Expanders - Apply equivalent subjects
Search modes - Boolean/Phrase Interface - EBSCOhost Research Databases
Search Screen - Advanced Search
Database - EconLit Display

Database 6: ABI Inform (N=170)

S1
ti,ab(adolescent* or (college NEAR/1 age?) or "early adult*" OR "generation Y" or "generation Z" or "Gen Z" or ("high school*" NEAR/1 age*) or millennial* or "new worker*" or teen* or "young adult*" OR "young people" or "young person?" or youth) OR MAINSUBJECT.EXACT("Young adults")

S2
ti,ab(episodic NEAR/2 (disease* or symptom* or health or condition*)) or ti,ab(fluctuating NEAR/2 (disease* or symptom* or health or condition*)) or ti,ab(unpredictable NEAR/2 (disease* or symptom* or health or condition*)) or ti,ab(dynamic NEAR/2 (disease* or symptom* or health or condition*)) or ti,ab(recurrent NEAR/2 (disease* or symptom* or health or condition*)) or ti,ab(hidden NEAR/2 (disease* or symptom* or health or condition*)) or ti,ab(invisible NEAR/2 (disease* or symptom* or health or condition*)) OR ti,ab("bowel disease*" OR cancer OR arthritis OR "chronic pain" or "chronic disease*" or "chronic fatigue" or "anxiety disorder*" OR (colitis NEAR/2 ulcerative) or "Crohn's disease" OR depression or OR diabetes OR epilepsy or disability* or fibromyalgia or HIV or lupus or "mental disorder*" or "mental health" or "mental illness" or migraine or "mood disorder*" or "multiple sclerosis" or "musculoskeletal disorder*" or "progressive condition*" or "rheumatic disease*" or bipolar or "post-traumatic stress" or "posttraumatic stress" or PTSD or PTSS or psoriasis or morphea or (autoimmune NEAR/2 condition*) or (autoimmune NEAR/2 disease*) or (autoimmune NEAR/2 disorder*) or "premenstrual symptom*")

S3
MAINSUBJECT.EXACT("Inflammatory bowel disease") OR MAINSUBJECT.EXACT("Cancer") OR MAINSUBJECT.EXACT("Chronic illnesses") OR MAINSUBJECT.EXACT("Anxiety disorders")

OR MAINSUBJECT.EXACT("Chronic fatigue syndrome") OR MAINSUBJECT.EXACT("Crohns disease") OR MAINSUBJECT.EXACT("Mental depression") OR MAINSUBJECT.EXACT("Diabetes") OR MAINSUBJECT.EXACT("Epilepsy") OR MAINSUBJECT.EXACT("Fibromyalgia") OR MAINSUBJECT.EXACT("Human immunodeficiency virus--HIV") OR MAINSUBJECT.EXACT("Arthritis") OR MAINSUBJECT.EXACT("Mental disorders") OR MAINSUBJECT.EXACT("Migraine") OR MAINSUBJECT.EXACT("Multiple sclerosis") OR MAINSUBJECT.EXACT("Musculoskeletal diseases") OR MAINSUBJECT.EXACT("Rheumatic diseases") OR OR MAINSUBJECT.EXACT("Bipolar disorder") OR MAINSUBJECT.EXACT("Lupus") OR MAINSUBJECT.EXACT("Post traumatic stress disorder") OR MAINSUBJECT.EXACT("Psoriasis") OR MAINSUBJECT.EXACT("Scleroderma") OR MAINSUBJECT.EXACT("Autoimmune diseases")

S4
S2 OR S3

S5
S1 AND S4

S6
ti,ab("work-integrated learning" OR (career NEAR/2 advice) OR (career NEAR/2 counsel*) OR "cooperative education" OR accommodat* OR (employment NEAR/3 (ready or readiness)) OR (employment NEAR/3 support*) OR (employment NEAR/3 transition*) OR "employment polic*" OR "employment program?" OR "vocational rehabilitation" or ("school to work" NEAR/2 transition) OR reskilling OR upskilling OR (work* NEAR/2 accessibility) OR (work* NEAR/2 inclusion) OR (employment NEAR/2 accessibility) OR (employment NEAR/2 inclusion) OR ("labo?r market" NEAR/2 (policies or policy)) OR ("labo?r market" NEAR/2 participat*) OR ("work disability" NEAR/2 prevention) OR "labo?r market integration" OR "labo?r market engagement" OR "job stability" OR "job search*" OR "labo?r market entry" OR ("labo?r market" NEAR/2 advancement*) OR "employee intensive program*" OR "job subsid*" OR practicum? OR internship? OR apprenticeship? or "job readiness" or self-employment OR "social enterprise?" OR start-up? OR incubator? OR "seed money" OR entrepreneurship OR "small business*") OR MAINSUBJECT.EXACT("Vocational rehabilitation")

S7
ti,ab((income NEAR/2 support*) OR (disability NEAR/2 support*) OR (disability NEAR/2 benefit*) OR "employment insurance" OR "worker* compensation" OR "health insurance" OR (unemployment NEAR/2 benefit*) OR "social protection" OR "income security" OR (disab* NEAR/2 benefit*) OR "basic income" OR UBI OR "unemployment assistan*" OR workfare OR flexicurity OR "guaranteed minimum income" OR (state NEAR/1 benefit*) OR (uncompensated NEAR/1 care) OR welfare OR (disability NEAR/2 grant*) OR "disability support" OR (disability NEAR/2 "tax credit?") OR "disability savings plan" OR (accessibility NEAR/2 funding) OR MAINSUBJECT.EXACT("Social security") OR MAINSUBJECT.EXACT("Health insurance") OR MAINSUBJECT.EXACT("Workers compensation")

S8
ti,ab((training NEAR/1 skill?) OR "government plan?" OR (government NEAR/1 subsid*) OR (interview? NEAR/1 support*) OR (interview? NEAR/1 mock) OR mentoring OR (peer? NEAR/1 support*) OR "vocational guidance" OR (employment NEAR/2 placement) OR (workplace NEAR/2 placement) OR "higher education" OR "graduate education" OR "postgraduate training" OR "undergraduate education" OR (education* NEAR/1 status) OR (education* NEAR/1 attainment) OR (education* NEAR/1 level) OR (education* NEAR/1 difference?) OR (education* NEAR/1 inequality)

OR "academic achievement" OR diploma? OR "school graduation" OR dropout OR (school NEAR/1 retention) OR scholarship? OR "transition program?" OR "foundation year" OR "transition* year" OR (pathways NEAR/1 education*) OR (university NEAR/1 application*) OR "mature student?" OR "high school equivalency" OR GED OR "adult education" OR "adult learning" OR "continuing education" OR "pitch competition" OR hackathon? OR pushout?) OR MAINSUBJECT.EXACT("Continuing education")<br><br>S9<br>S6 OR S7 OR S8<br><br>S10<br>Ti,ab((health NEAR/2 difference*) OR (health NEAR/2 disparit*) or (health NEAR/2 equit*) or (self-reported NEAR/2 health) or (self-rated NEAR/2 health) or well-being OR "quality of life" or (activity near/2 limitation?) OR impairment OR (symptom* NEAR/2 severity) OR (disability NEAR/2 severity) OR "disease activity" OR "health status" OR injury or injuries OR (disease NEAR/2 progress*) OR (disease NEAR/2 impact*) OR MAINSUBJECT.EXACT("Quality of life") OR MAINSUBJECT.EXACT("Injuries")<br><br>S11<br>Ti,ab((social NEAR/2 depriv*) OR (social NEAR/2 disadvantag*) OR (social NEAR/2 equity) OR (social NEAR/2 inequity) OR (social NEAR/2 marginali?ed) OR (social NEAR/1 capital) OR (social NEAR/1 class) OR (social NEAR/2 condition*) OR (social NEAR/2 difference*) OR (socio NEAR/1 economic NEAR/1 position) OR (socio NEAR/1 economic NEAR/1 status) OR (socio NEAR/1 economic NEAR/1 variable*) OR (socioeconomic NEAR/2 attribut*) OR (socioeconomic NEAR/1 circumstance*) OR (socioeconomic NEAR/1 factor?) OR (socioeconomic NEAR/1 gradient?) OR (socioeconomic NEAR/1 health NEAR/1 difference*) OR (socioeconomic NEAR/1 position) OR (socioeconomic NEAR/1 status) OR (standard NEAR/2 living) OR "social inclusion" OR "financial status" OR "income status" OR "cultural capital" OR "cultural currency" OR "low income" OR poverty OR "social mobility" OR (social NEAR/1 accessibility)) OR MAINSUBJECT.EXACT("Socioeconomic factors") OR MAINSUBJECT.EXACT("Poverty")<br><br>S12<br>S10 OR S11<br><br>S5 AND S9 AND S12
Database 7:: ASSIA (N=633)<br><br>S1<br>ti,ab(adolescent* or (college NEAR/1 age?) or "early adult*" OR "generation Y" or "generation Z" or "Gen Z" or ("high school*" NEAR/1 age*) or millennial* or "new worker*" or teen* or "young adult*" OR "young people" or "young person?" or youth) OR MAINSUBJECT.EXACT("Young adults")<br><br>S2<br>ti,ab(episodic NEAR/2 (disease* or symptom* or health or condition*)) or ti,ab(fluctuating NEAR/2 (disease* or symptom* or health or condition*)) or ti,ab(unpredictable NEAR/2 (disease* or symptom* or health or condition*)) or ti,ab(dynamic NEAR/2 (disease* or symptom* or health or condition*)) or ti,ab(recurrent NEAR/2 (disease* or symptom* or health or condition*)) or ti,ab(hidden NEAR/2 (disease* or symptom* or health or condition*)) or ti,ab(invisible NEAR/2 (disease* or symptom* or health or condition*)) OR ti,ab("bowel disease*" OR cancer OR arthritis OR "chronic pain" or "chronic disease*" or "chronic fatigue" or "anxiety disorder*" OR (colitis NEAR/2 ulcerative) or "Crohn's

disease" OR depression or OR diabetes OR epilepsy or disability* or fibromyalgia or HIV or lupus or "mental disorder*" or "mental health" or "mental illness" or migraine or "mood disorder*" or "multiple sclerosis" or "musculoskeletal disorder*" or "progressive condition*" or "rheumatic disease*" or bipolar or "post-traumatic stress" or "posttraumatic stress" or PTSD or PTSS or psoriasis or morphea or (autoimmune NEAR/2 condition*) or (autoimmune NEAR/2 disease*) or (autoimmune NEAR/2 disorder*) or "premenstrual symptom*")

S3
MAINSUBJECT.EXACT("Inflammatory bowel diseases") OR MAINSUBJECT.EXACT("Cancer") OR MAINSUBJECT.EXACT("Chronic illnesses") OR MAINSUBJECT.EXACT("Chronic pain") OR MAINSUBJECT.EXACT("Anxiety disorders") OR MAINSUBJECT.EXACT("Chronic fatigue syndrome") OR MAINSUBJECT.EXACT("Crohn's disease") OR MAINSUBJECT.EXACT("Mental depression") OR MAINSUBJECT.EXACT("Diabetes") OR MAINSUBJECT.EXACT("Epilepsy") OR MAINSUBJECT.EXACT("Fibromyalgia") OR MAINSUBJECT.EXACT("Human immunodeficiency virus--HIV") OR MAINSUBJECT.EXACT("Arthritis") OR MAINSUBJECT.EXACT("Mental disorders") OR MAINSUBJECT.EXACT("Migraine") OR MAINSUBJECT.EXACT("Multiple sclerosis") OR MAINSUBJECT.EXACT("Musculoskeletal diseases") OR MAINSUBJECT.EXACT("Rheumatic diseases") OR OR MAINSUBJECT.EXACT("Bipolar disorder") OR MAINSUBJECT.EXACT("Lupus erythematosus") OR MAINSUBJECT.EXACT("Post traumatic stress disorder") OR MAINSUBJECT.EXACT("Psoriasis") OR MAINSUBJECT.EXACT("Autoimmune disorders")

S4
S2 OR S3

S5
S1 AND S4

S6
ti,ab("work-integrated learning" OR (career NEAR/2 advice) OR (career NEAR/2 counsel*) OR "cooperative education" OR accommodat* OR (employment NEAR/3 (ready or readiness)) OR (employment NEAR/3 support*) OR (employment NEAR/3 transition*) OR "employment polic*" OR "employment program?" OR "vocational rehabilitation" or ("school to work" NEAR/2 transition) OR reskilling OR upskilling OR (work* NEAR/2 accessibility) OR (work* NEAR/2 inclusion) OR (employment NEAR/2 accessibility) OR (employment NEAR/2 inclusion) OR ("labo?r market" NEAR/2 (policies or policy)) OR ("labo?r market" NEAR/2 participat*) OR ("work disability" NEAR/2 prevention) OR "labo?r market integration" OR "labo?r market engagement" OR "job stability" OR "job search*" OR "labo?r market entry" OR ("labo?r market" NEAR/2 advancement*) OR "employee intensive program*" OR "job subsid*" OR practicum? OR internship? OR apprenticeship? or "job readiness" or self-employment OR "social enterprise?" OR start-up? OR incubator? OR "seed money" OR entrepreneurship OR "small business*") OR MAINSUBJECT.EXACT("Vocational rehabilitation")

S7
ti,ab((income NEAR/2 support*) OR (disability NEAR/2 support*) OR (disability NEAR/2 benefit*) OR "employment insurance" OR "worker* compensation" OR "health insurance" OR (unemployment NEAR/2 benefit*) OR "social protection" OR "income security" OR (disab* NEAR/2 benefit*) OR "basic income" OR UBI OR "unemployment assistan*" OR workfare OR flexicurity OR "guaranteed minimum income" OR (state NEAR/1 benefit*) OR (uncompensated NEAR/1 care) OR welfare OR (disability NEAR/2 grant*) OR "disability support" OR (disability NEAR/2 "tax credit?") OR

"disability savings plan" OR (accessibility NEAR/2 funding) OR MAINSUBJECT.EXACT("Social security") OR MAINSUBJECT.EXACT("Health insurance")

S8
ti,ab((training NEAR/1 skill?) OR "government plan?" OR (government NEAR/1 subsid*) OR (interview? NEAR/1 support*) OR (interview? NEAR/1 mock) OR mentoring OR (peer? NEAR/1 support*) OR "vocational guidance" OR (employment NEAR/2 placement) OR (workplace NEAR/2 placement) OR "higher education" OR "graduate education" OR "postgraduate training" OR "undergraduate education" OR (education* NEAR/1 status) OR (education* NEAR/1 attainment) OR (education* NEAR/1 level) OR (education* NEAR/1 difference?) OR (education* NEAR/1 inequality) OR "academic achievement" OR diploma? OR "school graduation" OR dropout OR (school NEAR/1 retention) OR scholarship? OR "transition program?" OR "foundation year" OR "transition* year" OR (pathways NEAR/1 education*) OR (university NEAR/1 application*) OR "mature student?" OR "high school equivalency" OR GED OR "adult education" OR "adult learning" OR "continuing education" OR "pitch competition" OR hackathon? OR pushout?) OR MAINSUBJECT.EXACT("Continuing education")

S9
S6 OR S7 OR S8

S10
Ti,ab((health NEAR/2 difference*) OR (health NEAR/2 disparit*) or (health NEAR/2 equit*) or (self-reported NEAR/2 health) or (self-rated NEAR/2 health) or well-being OR "quality of life" or (activity near/2 limitation?) OR impairment OR (symptom* NEAR/2 severity) OR (disability NEAR/2 severity) OR "disease activity" OR "health status" OR injury or injuries OR (disease NEAR/2 progress*) OR (disease NEAR/2 impact*) OR MAINSUBJECT.EXACT("Quality of life") OR MAINSUBJECT.EXACT("Injuries")

S11
Ti,ab((social NEAR/2 depriv*) OR (social NEAR/2 disadvantag*) OR (social NEAR/2 equity) OR (social NEAR/2 inequity) OR (social NEAR/2 marginali?ed) OR (social NEAR/1 capital) OR (social NEAR/1 class) OR (social NEAR/2 condition*) OR (social NEAR/2 difference*) OR (socio NEAR/1 economic NEAR/1 position) OR (socio NEAR/1 economic NEAR/1 status) OR (socio NEAR/1 economic NEAR/1 variable*) OR (socioeconomic NEAR/2 attribut*) OR (socioeconomic NEAR/1 circumstance*) OR (socioeconomic NEAR/1 factor?) OR (socioeconomic NEAR/1 gradient?) OR (socioeconomic NEAR/1 health NEAR/1 difference*) OR (socioeconomic NEAR/1 position) OR (socioeconomic NEAR/1 status) OR (standard NEAR/2 living) OR "social inclusion" OR "financial status" OR "income status" OR "cultural capital" OR "cultural currency" OR "low income" OR poverty OR "social mobility" OR (social NEAR/1 accessibility)) OR MAINSUBJECT.EXACT("Socioeconomic factors") OR MAINSUBJECT.EXACT("Poverty")

S12
S10 OR S11

S5 AND S9 AND S12

Database 8: Sociological Abstracts (N=222)

S1
ti,ab(adolescent* or (college NEAR/1 age?) or "early adult*" OR "generation Y" or "generation Z" or "Gen Z" or ("high school*" NEAR/1 age*) or millennial* or "new worker*" or teen* or "young adult*" OR "young people" or "young person?" or youth) OR MAINSUBJECT.EXACT("Young adults")

S2
ti,ab(episodic NEAR/2 (disease* or symptom* or health or condition*)) or ti,ab(fluctuating NEAR/2 (disease* or symptom* or health or condition*)) or ti,ab(unpredictable NEAR/2 (disease* or symptom* or health or condition*)) or ti,ab(dynamic NEAR/2 (disease* or symptom* or health or condition*)) or ti,ab(recurrent NEAR/2 (disease* or symptom* or health or condition*)) or ti,ab(hidden NEAR/2 (disease* or symptom* or health or condition*)) or ti,ab(invisible NEAR/2 (disease* or symptom* or health or condition*)) OR ti,ab("bowel disease*" OR cancer OR arthritis OR "chronic pain" or "chronic disease*" or "chronic fatigue" or "anxiety disorder*" OR (colitis NEAR/2 ulcerative) or "Crohn's disease" OR depression or OR diabetes OR epilepsy or disability* or fibromyalgia or HIV or lupus or "mental disorder*" or "mental health" or "mental illness" or migraine or "mood disorder*" or "multiple sclerosis" or "musculoskeletal disorder*" or "progressive condition*" or "rheumatic disease*" or bipolar or "post-traumatic stress" or "posttraumatic stress" or PTSD or PTSS or psoriasis or morphea or (autoimmune NEAR/2 condition*) or (autoimmune NEAR/2 disease*) or (autoimmune NEAR/2 disorder*) or "premenstrual symptom*")

S3
MAINSUBJECT.EXACT("Cancer") OR MAINSUBJECT.EXACT("Chronic Illness") OR MAINSUBJECT.EXACT("Anxiety") OR MAINSUBJECT.EXACT("Fatigue") OR MAINSUBJECT.EXACT("Depression (Psychology)") OR MAINSUBJECT.EXACT("Diabetes") OR MAINSUBJECT.EXACT("Epilepsy") OR MAINSUBJECT.EXACT("Arthritis") OR MAINSUBJECT.EXACT("Mental Illness") OR MAINSUBJECT.EXACT("Affective Illness")

S4
S2 OR S3

S5
S1 AND S4

S6
ti,ab("work-integrated learning" OR (career NEAR/2 advice) OR (career NEAR/2 counsel*) OR "cooperative education" OR accommodat* OR (employment NEAR/3 (ready or readiness)) OR (employment NEAR/3 support*) OR (employment NEAR/3 transition*) OR "employment polic*" OR "employment program?" OR "vocational rehabilitation" or ("school to work" NEAR/2 transition) OR reskilling OR upskilling OR (work* NEAR/2 accessibility) OR (work* NEAR/2 inclusion) OR (employment NEAR/2 accessibility) OR (employment NEAR/2 inclusion) OR ("labo?r market" NEAR/2 (policies or policy)) OR ("labo?r market" NEAR/2 participat*) OR ("work disability" NEAR/2 prevention) OR "labo?r market integration" OR "labo?r market engagement" OR "job stability" OR "job search*" OR "labo?r market entry" OR ("labo?r market" NEAR/2 advancement*) OR "employee intensive program*" OR "job subsid*" OR practicum? OR internship? OR apprenticeship? or "job readiness" or self-employment OR "social enterprise?" OR start-up? OR incubator? OR "seed money" OR entrepreneurship OR "small business*") OR MAINSUBJECT.EXACT("Vocational rehabilitation")

S7
ti,ab((income NEAR/2 support*) OR (disability NEAR/2 support*) OR (disability NEAR/2 benefit*) OR "employment insurance" OR "worker* compensation" OR "health insurance" OR (unemployment NEAR/2 benefit*) OR "social protection" OR "income security" OR (disab* NEAR/2 benefit*) OR "basic income" OR UBI OR "unemployment assistan*" OR workfare OR flexicurity OR "guaranteed minimum income" OR (state NEAR/1 benefit*) OR (uncompensated NEAR/1 care) OR welfare OR (disability NEAR/2 grant*) OR "disability support" OR (disability NEAR/2 "tax credit?") OR

"disability savings plan" OR (accessibility NEAR/2 funding) OR MAINSUBJECT.EXACT("Social security") OR MAINSUBJECT.EXACT("Health insurance") OR MAINSUBJECT.EXACT("Workers Compensation Insurance")

S8
ti,ab((training NEAR/1 skill?) OR "government plan?" OR (government NEAR/1 subsid*) OR (interview? NEAR/1 support*) OR (interview? NEAR/1 mock) OR mentoring OR (peer? NEAR/1 support*) OR "vocational guidance" OR (employment NEAR/2 placement) OR (workplace NEAR/2 placement) OR "higher education" OR "graduate education" OR "postgraduate training" OR "undergraduate education" OR (education* NEAR/1 status) OR (education* NEAR/1 attainment) OR (education* NEAR/1 level) OR (education* NEAR/1 difference?) OR (education* NEAR/1 inequality) OR "academic achievement" OR diploma? OR "school graduation" OR dropout OR (school NEAR/1 retention) OR scholarship? OR "transition program?" OR "foundation year" OR "transition* year" OR (pathways NEAR/1 education*) OR (university NEAR/1 application*) OR "mature student?" OR "high school equivalency" OR GED OR "adult education" OR "adult learning" OR "continuing education" OR "pitch competition" OR hackathon? OR pushout?) OR MAINSUBJECT.EXACT("Continuing education")

S9
S6 OR S7 OR S8

S10
Ti,ab((health NEAR/2 difference*) OR (health NEAR/2 disparit*) or (health NEAR/2 equit*) or (self-reported NEAR/2 health) or (self-rated NEAR/2 health) or well-being OR "quality of life" or (activity near/2 limitation?) OR impairment OR (symptom* NEAR/2 severity) OR (disability NEAR/2 severity) OR "disease activity" OR "health status" OR injury or injuries OR (disease NEAR/2 progress*) OR (disease NEAR/2 impact*) OR MAINSUBJECT.EXACT("Quality of life") OR MAINSUBJECT.EXACT("Injuries")

S11
Ti,ab((social NEAR/2 depriv*) OR (social NEAR/2 disadvantag*) OR (social NEAR/2 equity) OR (social NEAR/2 inequity) OR (social NEAR/2 marginali?ed) OR (social NEAR/1 capital) OR (social NEAR/1 class) OR (social NEAR/2 condition*) OR (social NEAR/2 difference*) OR (socio NEAR/1 economic NEAR/1 position) OR (socio NEAR/1 economic NEAR/1 status) OR (socio NEAR/1 economic NEAR/1 variable*) OR (socioeconomic NEAR/2 attribut*) OR (socioeconomic NEAR/1 circumstance*) OR (socioeconomic NEAR/1 factor?) OR (socioeconomic NEAR/1 gradient?) OR (socioeconomic NEAR/1 health NEAR/1 difference*) OR (socioeconomic NEAR/1 position) OR (socioeconomic NEAR/1 status) OR (standard NEAR/2 living) OR "social inclusion" OR "financial status" OR "income status" OR "cultural capital" OR "cultural currency" OR "low income" OR poverty OR "social mobility" OR (social NEAR/1 accessibility)) OR MAINSUBJECT.EXACT("Socioeconomic factors") OR MAINSUBJECT.EXACT("Poverty")

S12
S10 OR S11

S5 AND S9 AND S12

Table S3: Methodological quality appraisal criteria and question weight

**Systematic review methodological quality criteria**	**Question weight**
Was there a clear statement of the aims of the research and was the research design appropriate to address the aims of the research?	2
Were sampling and recruitment methods (including inclusion/exclusion criteria) clearly described and similar for all participants?	2
Was recruitment (or participation) rate reported and adequate?	2
Were there important differences between those who participated and did not participate in the study with respect to key characteristics i.e., exposure(s) (including intervention(s)) and outcome(s))?	3
Were baseline characteristics described?	2
Was an intervention allocation method performed adequately?	3
Was the intervention process adequately described to allow for replication?	2
Was there any potential for contamination?	2
Was there any potential for co-intervention?	1
Was compliance with the intervention described and adequate?	2
Was the length of follow-up X amount of time or greater?	2
Was the loss of follow up (attrition) less than 35%?	2
Were there important differences between those who completed the study and those who withdrew with respect to key characteristics i.e., exposure(s) (including intervention(s)) and outcomes(s)?	3
Were the instruments/methods used to assess exposure(s) valid and reliable?	3
Were the instruments/methods used to assess the outcome(s) valid, reliable, and not prone to important sources of measurement bias?	3
Were the outcomes described at baseline and follow-up?	3
Was data collection with respect to exposure/outcome carried out equivalently for all participants?	3
Were all participants' outcomes analyzed by the groups to which they were originally allocated (intention-to-treat analysis)?	3
Were important covariates, confounders, or baseline differences (if necessary) accounted for in the study design and/or analysis?	2
Was there a direct between group comparison?	3

PRISMA 2020 Checklist

Section and Topic	Item #	Checklist item	Location where item is reported
**TITLE**			
Title	1	Identify the report as a systematic review.	Title page doc
**ABSTRACT**			
Abstract	2	See the PRISMA 2020 for Abstracts checklist.	Completed
**INTRODUCTION**			
Rationale	3	Describe the rationale for the review in the context of existing knowledge.	1-2
Objectives	4	Provide an explicit statement of the objective(s) or question(s) the review addresses.	1,2
**METHODS**			
Eligibility criteria	5	Specify the inclusion and exclusion criteria for the review and how studies were grouped for the syntheses.	2
Information sources	6	Specify all databases, registers, websites, organisations, reference lists and other sources searched or consulted to identify studies. Specify the date when each source was last searched or consulted.	2
Search strategy	7	Present the full search strategies for all databases, registers and websites, including any filters and limits used.	2, supplement files (Table S1)
Selection process	8	Specify the methods used to decide whether a study met the inclusion criteria of the review, including how many reviewers screened each record and each report retrieved, whether they worked independently, and if applicable, details of automation tools used in the process.	2-3
Data collection process	9	Specify the methods used to collect data from reports, including how many reviewers collected data from each report, whether they worked independently, any processes for obtaining or confirming data from study investigators, and if applicable, details of automation tools used in the process.	2
Data items	10a	List and define all outcomes for which data were sought. Specify whether all results that were compatible with each outcome domain in each study were sought (e.g. for all measures, time points, analyses), and if not, the methods used to decide which results to collect.	2-5, Table 2,3
	10b	List and define all other variables for which data were sought (e.g. participant and intervention characteristics, funding sources). Describe any assumptions made about any missing or unclear information.	N/A
Study risk of bias assessment	11	Specify the methods used to assess risk of bias in the included studies, including details of the tool(s) used, how many reviewers assessed each study and whether they worked independently, and if applicable, details of automation tools used in the process.	4, Supplement S2
Effect measures	12	Specify for each outcome the effect measure(s) (e.g. risk ratio, mean difference) used in the synthesis or presentation of results.	Table 3
Synthesis methods	13a	Describe the processes used to decide which studies were eligible for each synthesis (e.g. tabulating the study intervention characteristics and comparing against the planned groups for each synthesis (item #5)).	Supplement, texts about QA
	13b	Describe any methods required to prepare the data for presentation or synthesis, such as handling of missing	N/A

Section and Topic	Item #	Checklist item	Location where item is reported
		summary statistics, or data conversions.	
	13c	Describe any methods used to tabulate or visually display results of individual studies and syntheses.	N/A
	13d	Describe any methods used to synthesize results and provide a rationale for the choice(s). If meta-analysis was performed, describe the model(s), method(s) to identify the presence and extent of statistical heterogeneity, and software package(s) used.	N/A
	13e	Describe any methods used to explore possible causes of heterogeneity among study results (e.g. subgroup analysis, meta-regression).	N/A
	13f	Describe any sensitivity analyses conducted to assess robustness of the synthesized results.	N/A
Reporting bias assessment	14	Describe any methods used to assess risk of bias due to missing results in a synthesis (arising from reporting biases).	4, Supplement 2
Certainty assessment	15	Describe any methods used to assess certainty (or confidence) in the body of evidence for an outcome.	QA
**RESULTS**			
Study selection	16a	Describe the results of the search and selection process, from the number of records identified in the search to the number of studies included in the review, ideally using a flow diagram.	2-3
	16b	Cite studies that might appear to meet the inclusion criteria, but which were excluded, and explain why they were excluded.	Figure 1
Study characteristics	17	Cite each included study and present its characteristics.	2-3, Table 2
Risk of bias in studies	18	Present assessments of risk of bias for each included study.	Table 3
Results of individual studies	19	For all outcomes, present, for each study: (a) summary statistics for each group (where appropriate) and (b) an effect estimate and its precision (e.g. confidence/credible interval), ideally using structured tables or plots.	Table 3
Results of syntheses	20a	For each synthesis, briefly summarise the characteristics and risk of bias among contributing studies.	3-6
	20b	Present results of all statistical syntheses conducted. If meta-analysis was done, present for each the summary estimate and its precision (e.g. confidence/credible interval) and measures of statistical heterogeneity. If comparing groups, describe the direction of the effect.	N/A
	20c	Present results of all investigations of possible causes of heterogeneity among study results.	Table 3
	20d	Present results of all sensitivity analyses conducted to assess the robustness of the synthesized results.	N/A
Reporting biases	21	Present assessments of risk of bias due to missing results (arising from reporting biases) for each synthesis assessed.	N/A
Certainty of evidence	22	Present assessments of certainty (or confidence) in the body of evidence for each outcome assessed.	3-6, Table 3,
**DISCUSSION**			

Section and Topic	Item #	Checklist item	Location where item is reported
Discussion	23a	Provide a general interpretation of the results in the context of other evidence.	3-6
	23b	Discuss any limitations of the evidence included in the review.	3,6
	23c	Discuss any limitations of the review processes used.	6
	23d	Discuss implications of the results for practice, policy, and future research.	6
**OTHER INFORMATION**			
Registration and protocol	24a	Provide registration information for the review, including register name and registration number, or state that the review was not registered.	Impact of employment and income support interventions on the health of young adults with episodic disability: Findings from a systematic review (CRD42021268354)
	24b	Indicate where the review protocol can be accessed, or state that a protocol was not prepared.	Included on Prospero
	24c	Describe and explain any amendments to information provided at registration or in the protocol.	N/A
Support	25	Describe sources of financial or non-financial support for the review, and the role of the funders or sponsors in the review.	Funding statement on page 6
Competing interests	26	Declare any competing interests of review authors.	N/A (stated none declared on page 6)
Availability of data, code and other materials	27	Report which of the following are publicly available and where they can be found: template data collection forms; data extracted from included studies; data used for all analyses; analytic code; any other materials used in the review.	N/A

From: Page MJ, McKenzie JE, Bossuyt PM, Boutron I, Hoffmann TC, Mulrow CD, et al. The PRISMA 2020 statement: an updated guideline for reporting systematic reviews. BMJ 2021;372:n71. doi: 10.1136/bmj.n71

For more information, visit: http://www.prisma-statement.org/
